# β-Arrestin2-biased Drd2 agonist UNC9995 alleviates astrocyte inflammatory injury via interaction between β-arrestin2 and STAT3 in mouse model of depression

**DOI:** 10.1186/s12974-022-02597-6

**Published:** 2022-10-01

**Authors:** Yang Liu, Nanshan Song, Hang Yao, Siyuan Jiang, Yueping Wang, Ying Zheng, Yuanzhang Zhou, Jianhua Ding, Gang Hu, Ming Lu

**Affiliations:** 1 Department of Pharmacology, Nanjing University of Chinese Medicine, Nanjing, 210023 China; 2 Jiangsu Key Laboratory of Neurodegeneration, Department of Pharmacology, Nanjing Medical University, 101 Longmian Avenue, Nanjing, 211166 Jiangsu China; 3 Neuroprotective Drug Discovery Key Laboratory, Department of Pharmacology, Nanjing Medical University, Nanjing, 211166 China

**Keywords:** UNC9995, β-Arrestin2, Astrocytes, Depression, Drd2 agonist, cGAS–STING, STAT3

## Abstract

**Background:**

Major depressive disorder (MDD) is a prevalent and devastating psychiatric illness. Unfortunately, the current therapeutic practice, generally depending on the serotonergic system for drug treatment is unsatisfactory and shows intractable side effects. Multiple evidence suggests that dopamine (DA) and dopaminergic signals associated with neuroinflammation are highly involved in the pathophysiology of depression as well as in the mechanism of antidepressant drugs, which is still in the early stage of study and well worthy of investigation.

**Methods:**

We established two chronic stress models, including chronic unpredictable mild stress (CUMS), and chronic social defeat stress (CSDS), to complementarily recapitulate depression-like behaviors. Then, hippocampal tissues were used to detect inflammation-related molecules and signaling pathways. Pathological changes in depressive mouse hippocampal astrocytes were examined by RNA sequencing. After confirming the dopamine receptor 2 (Drd2)/β-arrestin2 signaling changes in the depressive mice brain, we then established the depressive mouse model using the β-arrestin2 knockout mice or administrating the β-arrestin2-biased Drd2 agonist to investigate the roles. Label-free mass spectrometry was used to identify the β-arrestin2-binding proteins as the underlying mechanisms. We modeled neuroinflammation with interleukin-6 (IL-6) and corticosterone treatment and characterized astrocytes using multiple methods including cell viability assay, flow cytometry, and confocal immunofluorescence.

**Results:**

Drd2-biased β-arrestin2 pathway is significantly changed in the progression of depression, and genetic deletion of β-arrestin2 aggravates neuroinflammation and depressive-like phenotypes. Mechanistically, astrocytic β-arrestin2 retains STAT3 in the cytoplasm by structural combination with STAT3, therefore, inhibiting the JAK–STAT3 pathway-mediated inflammatory activation. Furtherly, pharmacological activation of Drd2/β-arrestin2 pathway by UNC9995 abolishes the inflammation-induced loss of astrocytes and ameliorates depressive-like behaviors in mouse model for depression.

**Conclusions:**

Drd2/β-arrestin2 pathway is a potential therapeutic target for depression and β-arrestin2-biased Drd2 agonist UNC9995 is identified as a potential anti-depressant strategy for preventing astrocytic dysfunctions and relieving neuropathological manifestations in mouse model for depression, which provides insights for the therapy of depression.

**Supplementary Information:**

The online version contains supplementary material available at 10.1186/s12974-022-02597-6.

## Introduction

Depression has been associated with the dysfunction of neurotransmitter systems, mainly norepinephrine and serotonin [1]. Selective serotonin reuptake inhibitors (SSRIs) are the common first-line treatments for major depressive disorder (MDD) [2], although they are generally well-tolerated, SSRIs have known adverse effects, including movement problems, alopecia, somnipathy, and gastrointestinal problems [3, 4]. Other antidepressants used in clinic commonly show narrow-spectrum effects, high recurrence and inevitable adverse reactions [5, 6]. Dopamine (DA) is also proposed to play an important role in the pathophysiology of depression as well as in the mechanism of antidepressant drugs. Growing evidence implicates that decreased levels of dopamine neurotransmission are found in patients with depression [7]. Dopamine agonist pramipexole was discovered to have significant antidepressant effects in patients suffering from depression [8, 9]. Besides, the pharmacological selectivity suggested the effects of Ketamine, the newly characterized fast-acting antidepressant medication, are blocked by inhibition of dopamine signaling [10]. Therefore, dopamine and its agonists are highly involved in the progression of depression and targeting dopamine receptor might be a promising strategy for antidepressant treatments [11].

G-protein-coupled receptors (GPCRs), as the main targets for marketed drugs [12, 13], are demonstrated to be closely linked to the pathophysiology or treatment of depression [14]. As a classic GPCR, dopamine receptor 2 (Drd2) not only participates in G protein-dependent pathways by binding with Gαi protein, but also can recruit β-arrestins to activate the biased pathway [15]. Our previous study reveals that astrocytic Drd2 has anti-inflammatory effects and helps maintaining the neuroinflammatory homeostasis during ageing and age-related disease [16]. Nevertheless, whether the astrocytic Drd2 is involved in the progression of psychiatric disorders is unknown. Furthermore, we demonstrate that β-arrestin2-biased signaling pathway downstream of GPCRs is involved in the antidepressant effects of fluoxetine to promote hippocampal neurogenesis and relieve depressive-like phenotypes in depressed mice [17], implying a promising prospect to develop β-arrestin2-biased agonism as strategies for depression. UNC series drugs are discovered as unique β-arrestin-biased selective Drd2 ligands, showing a potent ability to suppress β-amphetamine and phencyclidine-induced hyperlocomotion in mice without inducing motoric side effects [18]. In consideration that biased agonists often show functionally selective binding effects with high specificity, maximal drug activity and minimized side effects are achievable [19]. With this, we have recently discovered that UNC9995 enhances interaction between β-arrestin2 and NLRP3 to suppress NLRP3 inflammasome activation and thus prevents neuronal degeneration [20]. Application of this concept furtherly inspires us to investigate the prospect of biased agonists as optimized therapeutics of MDD. Here, we exhaustively clarify the pharmacological effect of this UNC series of compound in the prevention of depressive-like behaviors by focusing on the astrocytic loss and dysfunctions.

Although multiple hypotheses have been proposed to clarify the pathogenesis of depression, the molecular mechanisms and effective treatment of depression still need to be discovered. Astrocyte dysfunctions have been highlighted as phenotypic markers including reduced packing density of glial cells, lower levels of astrocytic markers accompanied by morphologic alterations; as well as biological mechanisms of depression including astrocytic secretory molecules [21, 22]. Previous studies from our lab correspondingly show that astrocyte-mediated neuroinflammatory processes are significant pathological events promoting disease progression [23, 24]. Patients suffering from depression often display increased levels of pro-inflammatory cytokines, such as interleukin-1 (IL-1), interleukin-6 (IL-6) and tumor necrosis factor-α (TNF-α) [25, 26], as well as endogenous metabolites including nesfatin-1 and corticosterone [27]. Astrocytes can secrete and respond to these inflammatory factors to amplify the inflammatory signals and incline the immune homeostasis of the brain to neurotoxic microenvironment [28, 29]. Based on the elusive but crucial roles of astrocytes in neurological and psychiatric diseases, we sought to elucidate the genetic profiles of astrocytes with the aim to reveal the etiology of depression from the perspective of astrocytic features in the current study.

In this research, we perform chronic social defeated stress (CSDS) and chronic unpredictable mild stress (CUMS) mouse model for MDD so as to mimic its clinical presentations including anhedonia, social withdrawal, depression and despair. We then isolate astrocytes from the brain of depressive mice and employ RNA sequencing to trace the genetic profiles of astrocytes under depressive circumstances, in which we show that inflammatory genes are significantly increased especially IL-6, and inflammatory pathways including cGAS–STING signaling are highly activated. Meanwhile, we observe decreased contents of DA and its metabolite DOPAC, as well as significant changes of Drd2 and β-arrestin2 protein levels in the CUMS depression mouse model. We then investigate the involvement of Drd2/β-arrestin2 signaling in the inflammatory characteristics of astrocytes during depression, and we show that β-arrestin2 deletion aggravates depressive-like behaviors as well as astrocytic loss in the hippocampus of depressive mice. Furthermore, the biased Drd2 agonist UNC9995 improves depressive behavioral phenotypes of mice and the dysfunctions of astrocytes. Mechanistically, β-arrestin2 acts as a scaffold protein to interact with STAT3, therefore, inhibiting its phosphorylation and its subsequent nucleic translocation. UNC9995 activates the Drd2/β-arrestin2 signaling to prevent induction of inflammation-related genes transcription by JAK/STAT3. Anti-inflammatory effects of UNC9995 are also revealed in primary astrocytes by stimulated with IL-6, the foremost inflammatory cytokines of MDD, and we demonstrate that UNC9995 prevents astrocytic loss induced by high concentration of IL-6, which at biomolecular scale refines the cellular events of astrocytic loss in vivo. These findings reveal that Drd2/β-arrestin2 pathway is a potential therapeutic target for depression and β-arrestin2-biased Drd2 agonist UNC9995 provides insights for the therapy of depression.

## Methods

### Animals and treatment

All animal care and procedures were performed following national and international guidelines and were approved by the Animal Resource Centre, Nanjing Medical University. β-Arrestin2 knockout (*Arrb2*^−/−^) mice were generated by Nanjing Biomedical Research Institute of Nanjing University. *Arrb2*^−/−^ and wild-type C57BL/6 J mice (male, 3 months, 22–26 g) were used in our experiments. The number of animals was definitely indicated in the figure legend. Mice were housed in groups of 5 per cage with water and food ad libitum on a 12:12 h light cycle (lights on at 7 a.m.).

### Chronic stress models for MDD

Chronic stress, especially psychosocial stressors, is one well-known risk factor for the development of depression. Here, we established two chronic stress models, including chronic unpredictable mild stress (CUMS), and chronic social defeat stress (CSDS), to complementarily recapitulate depression-like behaviors in rodents [30]. For CSDS model, Defeated mice after CSDS modeling display a special intense aversive but do not show differences in aversion to nonsocial stimuli [31]. CUMS simulates stressors from external material environment stimuli to mimic stress-induced behavioral changes that resemble human depressive symptoms, which can truly simulate some causes and symptoms of depressive patients [32].

*Chronic social defeated stress (CSDS) model* CSDS was performed as previously described [33, 34]. All CD-1 aggressors were screened for aggressive behavior before use according to published protocols. Experimental C57BL6/J mice encountered a novel CD-1aggressor for 10 min daily over 10 consecutive days. Mice were housed opposite a perforated Plexiglas barrier between defeat sessions to enable continuous sensory contact with the aggressor. After 10 days, experimental mice were singly housed overnight and underwent social interaction testing the following day. Then, it was tested by behavior test to divide into resistant mice group and susceptible mice group. Susceptible mice were selected to be administrated with UNC9995 (2 mg/kg/d, once a day, i.p).

*Chronic unpredictable mild stress (CUMS) model* CUMS procedure was conducted, as previously reported [35]. A series of procedure-like inversion of day/night light cycle, 45° tilted cage, emptying the cage, food and water deprivation overnight, restraint, and pairing with any another, clipping for 10 min, are applied as the mild and unpredictable stress with certain duration, nature, and frequency. Three stressors were imposed randomly everyday but not repeatedly within 3 continue day for 8 weeks. 8 weeks later, 8–10 mice were selected randomly to be administrated with UNC9995 (2 mg/kg/d, once a day, i.p) for drug treatment. The CUMS procedures were carried out continuously during the whole drug treatment period.

### Behavioral procedures

Behavioral assays were performed 3 days after successful establishment of CSDS or CUMS model.

*Social interaction (SI) test* One day after the last stress session, social interaction test was conducted. It was measured using the two-part test. In the first part, C57BL/6 J mice were placed in an open field (50 cm × 50 cm × 50 cm) within an acrylic mesh cover (10 cm wide × 10 cm long × 50 cm high) which the area around was defined as interaction zone (25 × 15 cm). Time spent in the interaction zone was measured over 2.5 min during the first part in which the mesh cover was empty. Mice were then returned to home cage for 30 s. In the second part of test, an unfamiliar CD1 mouse was placed inside the mesh cover and the same metrics were measured. The social contact rate of each C57BL/6 J mouse was calculated as follows: SI ratio = target time /blank time. Animals were considered as sensitive to stress if SI value less than 1 (Susceptible group), or resilient to stress if greater than 1(Resilient group).

*Open Field Test* An open field area (50 cm × 50 cm × 50 cm) made of blue PVC was used to assess spontaneous activity. Animals were habituated to the room 2 h prior to test, Movement and locomotor activity of 4 mice was monitored simultaneously in 4 boxes over 5 min with a camera above the arenas and the TopView Behavior Analyzing System (American CleverSys Inc.) was used to measure the spontaneous activity of the animal. Total distance and the time spent in center area were analyzed.

*Forced Swimming Test (FST)* Mice were placed in 1.6 L of water (25 ± 2 °C) in 2 L cylindrical glass beaker for 6 min. The activity of 4 mice was recorded simultaneously in four different beakers, separated by a gray wall, and the immobility time was recorded automatically during the last 5 min. Mice were only considered immobile when floating in the water, without struggling. The test was recorded by a video camera and analyzed by homecage behavior system (American CleverSys Inc).

*Sucrose Preference Test (SPT)* Mice were kept in their home cages and given the choice between two washed bottles (50 ml tubes with nibbles) containing water or 1% (w/v) sucrose dissolved in water for 24 h. Bottles were switched every 12 h to exclude possible placement bias. Animals were deprived of water for 24 h the day before the experiment. Baseline consumption of water and sucrose solutions was established during 3 consecutive days. Subsequently, the effect of UNC9995 on bottle preference was assessed on 3 consecutive days. Preference for sucrose was calculated as 100% × (Weight sucrose /Total weight).

*Tail Suspension Test (TST)* Mice were suspended individually in the middle of the top of the experiment box, 30 cm above the ground and the tape was placed about 4 cm from the tip of the tail. All the animals were suspended for a total of 6 min and the duration of immobility was recorded during the last 5 min of the test. The test was recorded by a video camera and analyzed by homecage behavior system (American CleverSys Inc).

*Correlation analysis* After collecting the data of the two factors, respectively, we performed *Z* value transformation on the data set, respectively. The transformation formula is: *Z* Score = (*x* − μ)/σ (*x*: actual value μ: average value σ: standard deviation), and then we obtain the correlation formula between the two factors according to the converted values, the formula formed as dualistic formula, at last we obtain the *R* value of the corresponding formula.

### Cell culture and treatment

Primary astrocytes were dissociated from 1- to 3-day-old newborn mice, and cultured as described as previous [20], 7-day-old cultures were pre-treated with UNC9995 (10 μM) for 1 h and subsequently stimulated with IL-6 (Peprotech, USA) for 2 4 h. UNC9995 was obtained from Xiaolong Wang, professor of Nanjing University of Chinese Medicine. Then, cell supernatant was collected for ELISA, astrocytes collected for immunoblotting, qRT-PCR and flow cytometry.

### RNA sequencing

The purity of the anti-ACSA-2 microbead kit isolated astrocytes from hippocampus of susceptible mice was verified by flow cytometry. Then, cells were analyzed by smart-seq at LC Bio (Zhejiang, China). Sequencing libraries were generated using the NEB Next^®^UltraTM RNA Library Prep Kit for Illumina following the manufacturer’s recommendations, and index codes were added to attribute sequences to each sample. Clustering of the index-coded samples was performed on a cBot Cluster Generation System using the TruSeq PE Cluster Kit v4-cBot-HS (Illumina) according to the manufacturer’s instructions. After cluster generation, the libraries were sequenced on an Illumina HiSeq 2500 platform, and paired-end reads were generated. The adaptor sequences and low-quality sequence reads were removed from the data sets. Raw sequences were transformed into clean reads after data processing. These clean reads were then mapped to the reference genome sequence. Only reads with a perfect match or one mismatch were further analyzed and annotated based on the reference genome. TopHat2 tools were used to map sequences to the reference genome.

### Immunohistochemistry and 3D reconstruction

Brain hippocampal slices (20 µm) were rinsed carefully in PBS followed by 3% H_2_O_2_ for 30 min to quench the endogenous peroxidase activity then incubated with 0.3% Triton X-100 in PBS supplemented 5% BSA for 1 h. After that, the sections were incubated with anti-GFAP antibody at 4 °C overnight. After extensive washing, brain slices were incubated with secondary anti bodies for 1 h at room temperature. Finally, the slides were incubated with Diaminobenzidin (DAB) for 5 min. The number of GFAP-positive cell in the dentate gyrus (DG) was assessed using the optical fractionator. Serial images of the DG were captured using the optical fractionator. Then, it was followed by 3D reconstruction and Sholl analysis (Neurolucida 11).

### Immunofluorescence

The immunofluorescent staining method was conducted as described before [36]. The primary antibody mouse anti-GFAP (Millipore, #MAB360, 1:1000), rabbit anti-β-arrestin2 (Cell Signaling Technology, #3857, 1:200), rabbit anti-IBA-1 (abcam, #019-19741, 1:1000), mouse anti-Phospho-Stat3 (Tyr705) Ab (Cell Signaling Technology, #9131, 1:200), mouse anti-Stat3 (124H6) Ab (Cell Signaling Technology, #9139, 1:200), rabbit anti-cGAS (E5V3W) Ab (Cell Signaling Technology, #79978S, 1:200). Goat anti-Rabbit IgG (Alexa Fluor^®^ 488 conjugate, Invitrogen, #A11008, 1:1000), Goat anti-Mouse IgG (Alexa Fluor^®^ 555 conjugate, Invitrogen, #A21422, 1:1000) secondary antibody were used in present study. The images were captured using the optical fractionator.

### Propidium iodide (PI)/Hoechst staining

Hoechst 33,342 (Sigma, #B2261) stained the nuclei of both live cells and dead cells, whereas PI (Invitrogen, #P1304MP) only stained the nuclei of dead cells. After the treatment of astrocytes, the cells were washed with PBS before stained with PI (10 µM) at 37 °C for 10 min. The cells were then washed with PBS, stained with Hoechst 33,342 (5 µg/mL) for 10 min, and fixed in 4% formaldehyde for 10 min. After being washed with PBS, the images were taken by fluorescence microscopy (Olympus, Tokyo, Japan).

### Multiplex tyramide signal amplification

Brain slices were treated as previously described for immunohistochemistry [20]. After scavenging endogenous peroxidase activity (Histova, #HP3P100) and blocking non-specific antigens (Histova, #GTBB30). Antigens were detected using the following protocol. Briefly, each primary antibody was incubated above 18 h in a humidified chamber at 4 °C, followed by detection using the HRP-conjugated secondary antibody and TSA-dendron-fluorophores (Histova, NEON 4-color IHC Kit for cryosection, #NEFP450), after which the primary and secondary antibodies were thoroughly eluted in stripping solution (Histova, #ABCCC30) for 30 min at 37 °C. In a serial fashion, each antigen was labeled by distinct fluorophores. After all the antibodies were detected sequentially, the slices were finally stained with Hoechst. Multiplex TSA-stained brain slices were imaged using the confocal laser scanning microscopy platform CarlZeiss LSM710.

### Western blotting

Western blotting analysis was conducted as previously described [37]. Antibodies: rabbit anti-β-arrestin2 (C16D9) Ab (#3857, 1:1000), rabbit anti-phospho-Jak2 (Tyr1007/1008) Ab (#3771, 1:1000), rabbit anti-Jak2 (D2E12) Ab (#3230, 1:1000), mouse anti-Phospho-Stat3 (Tyr705) Ab (#9131, 1:1000), mouse anti-Stat3 (124H6) Ab (#9139, 1:1000), mouse anti-bcl2 (124) Ab (#15071S, 1:1000), rabbit anti-Bax (D2E11) Ab (#5032S, 1:1000), rabbit anti-STING (D2P2F) Ab (#13647S, 1:1000), rabbit anti-p-STING (Ser366) Ab (#50907S, 1:1000), rabbit anti-cGAS (E5V3W) Ab (#79978S, 1:1000), rabbit anti-IBA-1 (#019-19741, 1:1000) from abcam, goat anti-rabbit IgG (#074-1516, 1:800) and goat anti-mouse IgG (#074-1806, 1:800) from Cell Signaling Technology; mouse anti-GFAP Ab (#MAB360, 1:1000) from Millipore; mouse anti-β-actin Ab (#66009-1-Ig, 1:5000) from Proteintech; rabbit anti-β-actin Ab (#ab8227, 1:5000) from abcam.

### Flow cytometry

After sucking out the culture medium, cells were rinsed with PBS for three times. Then, the cells were digested with the Trypsin (ThermoFisher, #15050065) without EDTA Solution and centrifuged in 1000 g for 5 min. Apoptotic cells stained with Annexin V-FITC/PI apoptosis kit (Vazyme, #A211-01), and subsequently detected using flow cytometry.

### qRT-PCR

Extraction of total RNA from brain hippocampal tissue and cells using TRIzol reagent (Invitrogen, #15596026). qRT-PCR was measured using 2 × SYBR Green qPCR Master Mix (Vazyme, #Q341) in the StepOnePlus instrument (Applied Biosystems). The sequences of qPCR primers were listed in Additional file 1: Table S1.

### ELISA

The concentration of IL-1β, IL-6, TNF-α, and INF-β in the serum and cell culture was detected by mouse IL-1β ELISA Kit, IL-6 ELISA Kit, TNF-α ELISA Kit and INF-β ELISA Kit (ExCell) according to the manufacturer's instructions.

### Label-free mass spectrometry

The method was described as previously [37]. Anti-β-arrestin2 immunoprecipitated protein (500 μg for each sample) was digested according to the FASP procedure. Briefly, the detergent, DTT and other low-molecular-weight components were removed using 200 µl UA buffer by repeated ultrafiltration facilitated by centrifugation. The protein suspension was digested with 3 µg trypsin in 40 µl 25 mM NH_4_HCO_3_ overnight at 37 °C. After digestion, the peptides in each sample were desalted on C18 cartridges, concentrated by vacuum centrifugation and reconstituted in 40 µl of 0.1% (v/v) trifluoroacetic acid. MS experiments were performed on a Q Exactive mass spectrometer coupled to an EasynLC. Five micrograms of the peptide were loaded onto a C18-reversed-phase column in buffer A (2% acetonitrile and 0.1% formic acid) and separated with a linear gradient of buffer B (80% acetonitrile and 0.1% formic acid) at a flow rate of 250 nL.min^−1^ controlled by IntelliFlow technology over 120 min. MS data were acquired using a data-dependent top10 method, which dynamically chooses the most abundant precursor ions from the survey scan (300–1800 m/z-1) for HCD fragmentation. Determination of the target value is based on predictive automatic gain control. Survey scans were acquired at a resolution of 70,000 at m/z-1200, and the resolution for HCD spectra was set to 17,500 at m/z-1200. The instrument was run with peptide recognition mode enabled. MS experiments were performed in triplicate for each sample. MS data were analyzed using MaxQuant software version 1.3.0.5 and were searched against the UniProtKB database.

### Proximity ligation assay (PLA)

Protein interactions in astrocytes were detected using the Duolink^®^ PLA assay kit (Sigma-Aldrich, DUO92101) following the manufacturer’s protocol. After treatments, cells on slides were fixed with 4% paraformaldehyde and permeabilized with 0.3% Triton X-100 in PBS. Blocking solution was added to the slides, which were incubated at 37 °C for 1 h. Then, the slides were incubated with rabbit primary β-arrestin2 antibody (Proteintech, #10171-1-AP, 1:200) and mouse primary STAT3 antibody (Santa Cruz, #sc-8019, 1:200) at 4 °C overnight and then with PLA probe solution for 1 h at 37 °C. After being washed, the slides were incubated for 30 min at 37 °C and then incubated with the amplification solution at 37 °C for 100 min protected from light. Finally, cell nuclei were stained with DAPI (Invitrogen, D1306), and the slides were imaged using the confocal laser scanning microscopy platform Leica TCS SP8.

### Cell counting kit-8 (CCK8) analysis

Astrocytes planting in 96-well plate at 5000 cells/well were treated with cck8 (10 µl/well) after IL-6 (0, 50, 100, 200, 300, 500 ng/ml) stimulation. Then, cell viability was detected using Thermo Fisher Microplate Reader in wavelength 450 nm.

### Statistical analysis

The data were all expressed in the means ± SEM. Mean values differences were assessed using two-way or one-way ANOVA analysis. Significant differences existed at *P* < 0.05.

## Results

### CSDS induces astrocyte dysfunction and inflammatory microenvironment in the hippocampus of mice

**Fig. 1:**
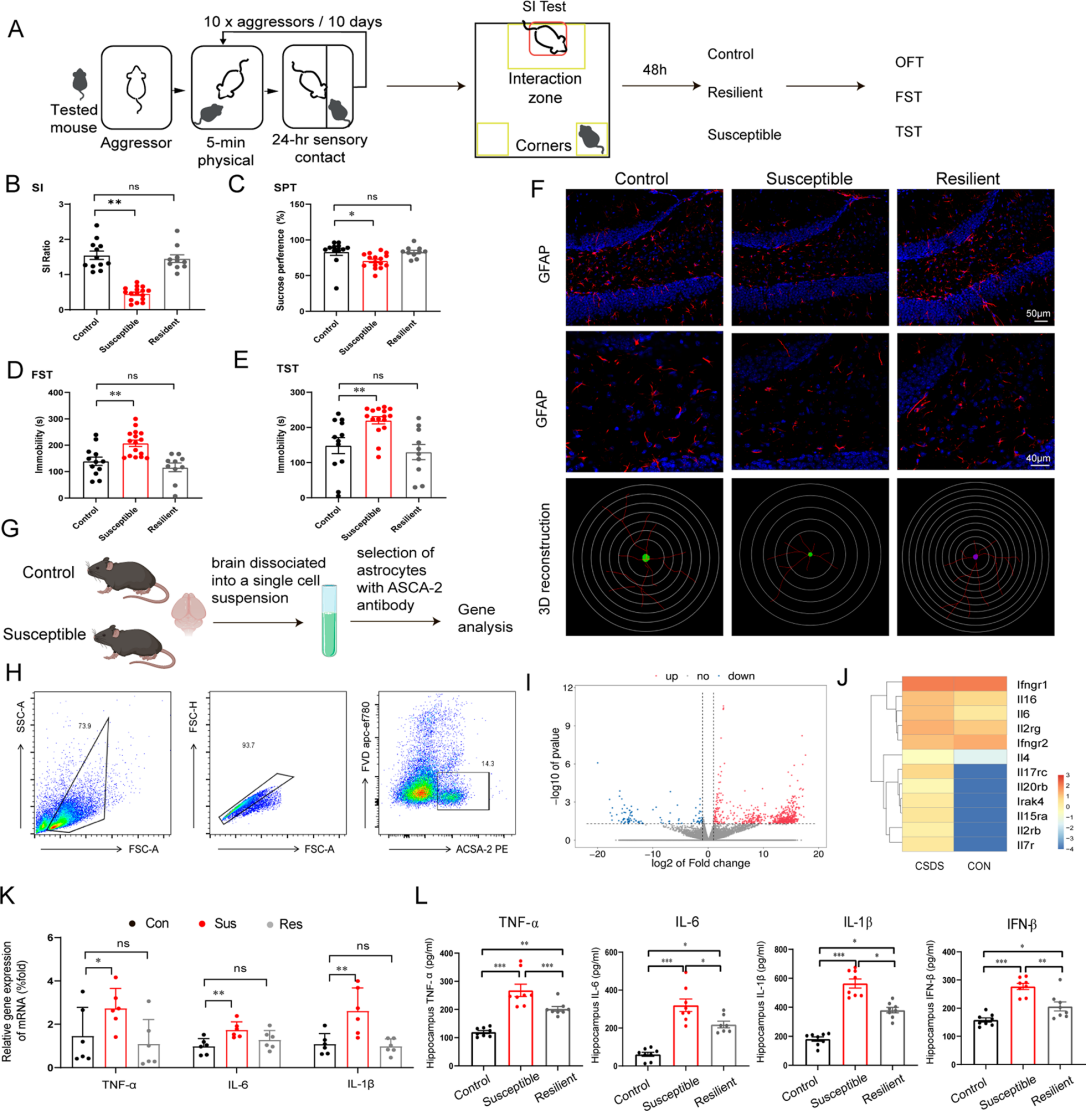
CSDS induces astrocyte dysfunction and inflammatory microenvironment in the hippocampus of mice. **A** Schematic diagram of the model of CSDS. Behavior test of SI ratio (**B**), sucrose preference test (**C**), forced swim test (**D**), and tail suspension test (**E**) were detected (Control group: *n* = 12; Susceptible group: *n* = 16; Resilient group: *n* = 10). **F** Immunofluorescence staining the marker of astrocytes (GFAP) in the hippocampus and 3D reconstruction. Red, GFAP; Blue, Hoechst. Scale bar, 50 μm. Enlarge vision, Scale bar, 40 μm. **G** Schematic diagram of isolating ACSA-2 positive astrocytes from the hippocampus of control and susceptible mice. **H** Purity of astrocytes isolated by ACSA-2 was identified with flow cytometry. **I** Heatmap of the RNA sequencing performed in WT and CSDS susceptible mice. **J** Cytokines and receptors of cytokines such as the interleukin family were listed in susceptible mice compared with controls. Levels of cytokines such as TNF-*α*, IL-6, IFN-*β*, and IL-1*β* were detected by qRT-PCR (**K**) and ELISA (**L**). **K** *n* = 6; **L** *n* = 8. Data are analyzed using one-way ANOVA, then combined with Dunnett’s to assess the differences between groups. ns, *P* > 0.05; *P < 0.05, **P < 0.01, ***P < 0.001

In this study, we first established CSDS mouse model to mimic clinical presentations of MDD. The CSDS procedures are shown in Fig. 1A. As not all mice exposed to CSDS are susceptible to these effects, we sub-divided these mice into resilient and susceptible group. Following 10-day establishment of CSDS mouse model, social interaction test was conducted to distinguish the susceptibility in CSDS exposed mice (Fig. 1B). Then, depressive-like behaviors of control, resilient and susceptible mice were evaluated by SPT, FST and TST (Fig. 1C–E). As shown in Fig. 1C, CSDS treatment decreased sucrose preference of susceptible mice compared with control group, implying the anhedonia conditions of susceptible mice. The forced swim test and tail suspension test were also performed to evaluate behavioral despair of the mice, and it was found that susceptible mice showed significantly increased time of immobility both under the forced-swimming condition (Fig. 1D) and the tail suspension stress compared with the control mice (Fig. 1E). As to the neuropathological changes, immunofluorescent staining showed decreased number of astrocytes in hippocampus of the susceptible mice (Fig. 1F), which is regarded as a characteristic feature of astrocytic pathology in MDD. Consistently, massive loss of astrocytes accompanied with activation of microglia were manifested in the hippocampus of CUMS mice, whereas the number of NeuN^+^ showed no significant change (Additional file 1: Fig. S1A–E).

To dissect the factors contributing to the astrocytic dysfunctions, we isolated astrocytes (ACSA-2^+^) from brain of the control and susceptible mice of the CSDS model (Fig. 1G) to employ RNA sequencing. The purity of the anti-ACSA-2 isolated astrocytes was verified by flow cytometry (Fig. 1H). RNA sequencing of purified astrocytes from depressive mice showed that 624 genes were up-regulated and 110 genes were down-regulated significantly (Fig. 1I and Additional file 1: Fig. S2), among which inflammatory genes and their receptors, especially the interleukin family were notably increased in astrocytes of susceptible mice compared with controls (Fig. 1J). As clinic data demonstrated a robust increase of pro-inflammatory cytokines, we additionally verified the levels of typical inflammatory genes and the corresponding inflammatory factors in the hippocampus by multiple methods. As shown in Fig. 1K, L, TNF-α, IL-6, IFN-β and IL-1β were increased significantly in the hippocampus of susceptible mice at both the mRNA levels and protein levels, among which IL-6 showed the highest fold change. Same results were obtained in the CUMS mouse model (Additional file 1: Fig. S1F). These results indicate that chronic stress induces the loss of astrocytes and increases level of inflammatory cytokines in the hippocampus of mice.

### CSDS activates inflammatory pathway including cGAS–STING pathway in mouse model

**Fig. 2:**
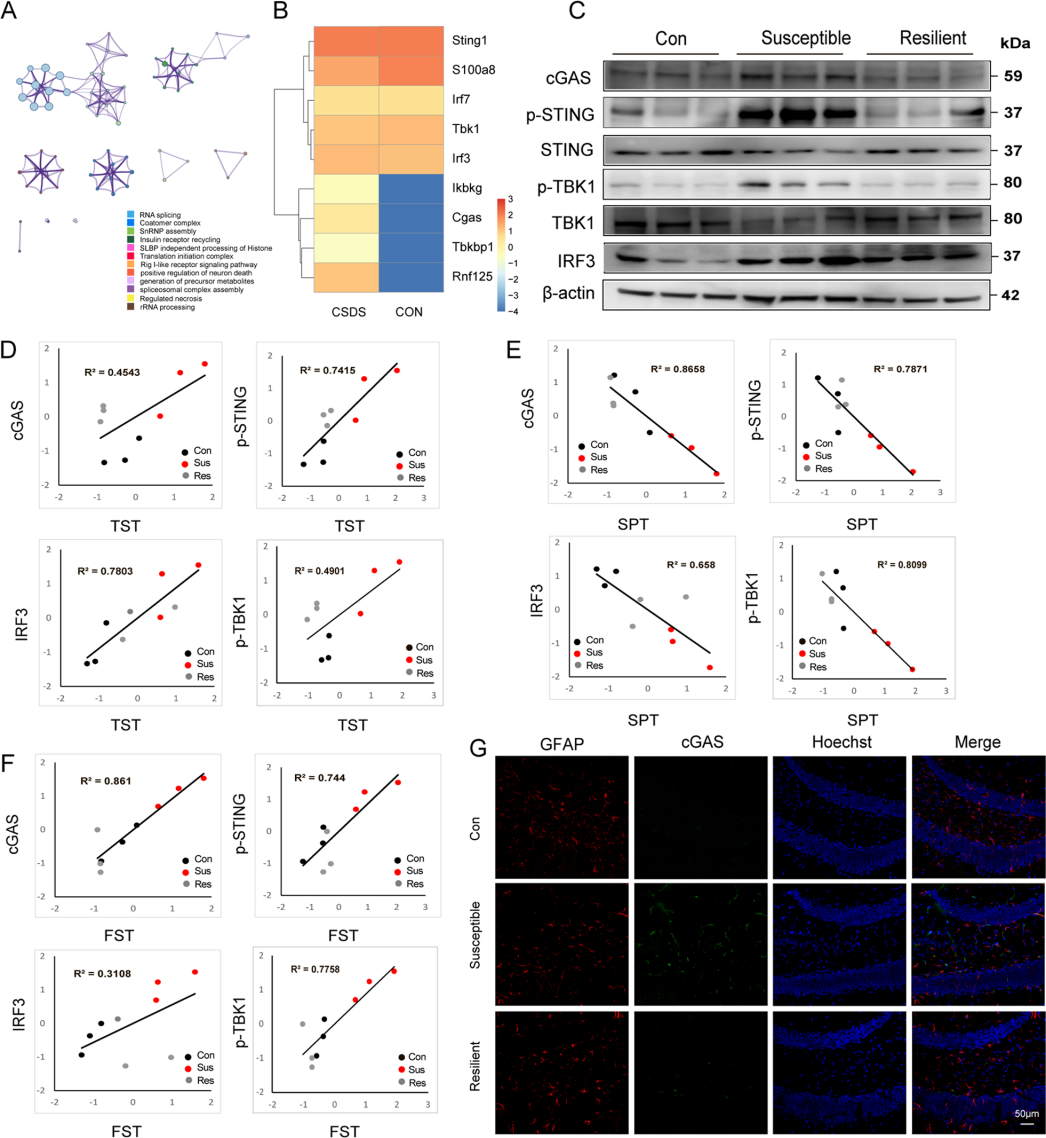
CSDS aggravates depressive-like behaviors via cGAS/STING pathway in the mouse model. **A** Enrichment GO analysis by cluster from Medscape database compared the susceptible mice with the control group. **B** Genes in the RIG-I-like receptor signaling pathway were up-regulated in CSDS susceptible group. **C** Protein level of cGAS, p-STING, TBK1 and IRF3 were detected in the control, susceptible, and resilient mice groups. Correlation analysis evaluated relevance between cGAS, p-STING, IRF3 and representations of behavior tests in tail suspension test (**D**), sucrose preference test (**E**) and forced swimming test (**F**). **G** Co-localization of GFAP/cGAS in the hippocampus of control, susceptible and resilient mice group. Red, GFAP; Green, cGAS; Blue, Hoechst. Scale bar, 50 μm

We also dive deeply to the RNA-sequencing data by conducting Enrichment GO analysis using cluster from Medscape database, in which we screened out retinoic acid-inducible gene I (RIG-I) RIG1-like signaling pathway (Fig. 2A, B). This pathway initiates the inflammatory response and induce inflammatory gene expression through RLR induction of downstream effector molecules, such as IFN-β, IL-6 and other pro-inflammatory cytokines [38, 39]. Furthermore, signaling molecules in the RIG1-like pathway, including cGAS, p-STING, p-TBK1 and IRF3 were significantly up-regulated in susceptible mice of the CSDS model (Fig. 2C and Additional file 1: Fig. S3A–F). We also conducted correlation analysis between the change of RIG1-like pathway with depressive phenotypes and we showed that increased levels of cGAS, p-STING, p-TBK1 and IRF3 were positively related to the extent of the behavioral tests (Fig. 2D, E), negatively related to the extent of sucrose preference test (Fig. 2F), which implied that RIG1-like pathway was accompanied with the extent of depressive phenotypes. As shown in Fig. 2G, the colocalization of cGAS and GFAP, the astrocytic marker, was highly increased, consistently implying the activation of RIG1-like signaling pathway in susceptible mice. These results demonstrate that depressive stressors activate cGAS–STING pathway in the hippocampal astrocytes of the CSDS-induced depressive mice.

### Drd2-biased β-arrestin2 pathway is involved in the loss of astrocytes

**Fig. 3:**
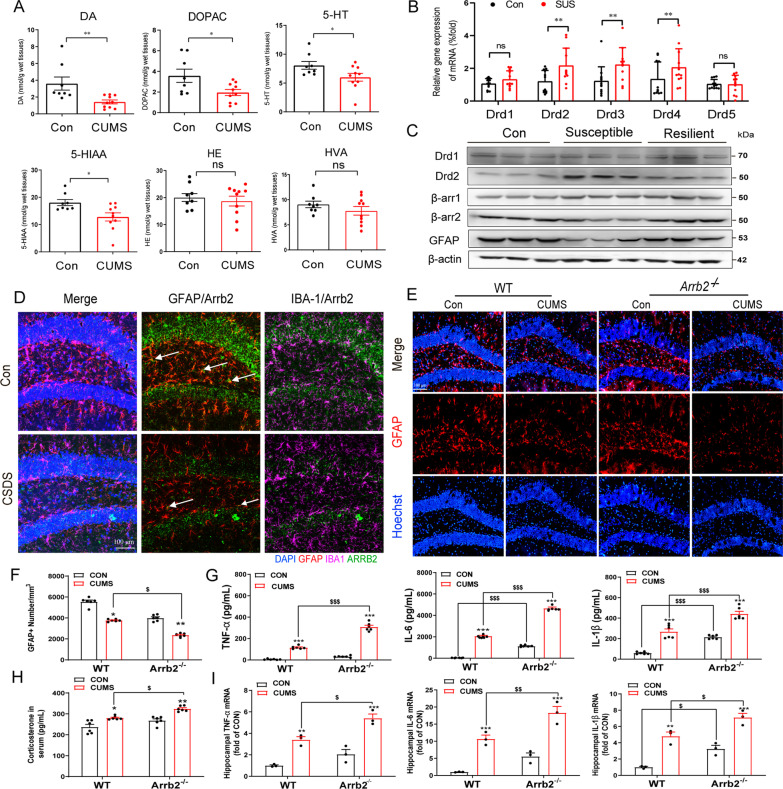
Drd2-biased β-arrestin2 pathway was involved in the loss of astrocytes. **A** Concentrations of DA, DOPAC, 5-HT, 5-HIAA, HE, and HVA in the brain were detected by HPLC. (Control group: *n* = 8; CUMS group: *n* = 10). **B** mRNA levels of the dopamine receptor family (Drd1, Drd2, Drd3, Drd4, and Drd5) in the hippocampus were detected by qRT-PCR. (Control group: *n* = 12; Susceptible group: *n* = 12). **C** Protein level of Drd1, Drd2, β-arrb2, β-arrb2 and GFAP were detected in the control, susceptible, and resilient mice groups. **D** Colocalization of GFAP (the marker of astrocytes) with β-arrestin2 and colocalization of IBA-1 (the marker of microglia) with β-arrestin2 by immunofluorescent staining in the hippocampus of Control and CSDS groups. Red, GFAP; Pink, IBA-1; Green, β-arrestin2; Blue, DAPI. Scale bar, 100 μm. Arrow: GFAP^+^ astrocytes co-localize with β-arrestin2 in the hippocampus. **E** Immunofluorescence of GFAP in the hippocampus of WT and *Arrb2*^*−/−*^ CUMS mouse model. Red, GFAP; Blue, Hoechst. Scale bar, 100 μm. **F** Counting GFAP^+^ astrocytes in the hippocampus of WT and *Arrb2*^*−/−*^ CUMS mouse models. **G** Levels of cytokines such as TNF-*α*, IL-6, and IL-1*β* were detected by RT-PCR in the hippocampus of WT and CUMS mouse models (*n* = 6). Corticosterone (**H**) and levels of cytokines (**I**) such as IL-1*β*, IL-6, and TNF-*α* were detected by ELISA in the WT and CUMS mouse model plasma (**H** *n* = 6; **I** *n* = 3). **A**, **B** Bars and error flags represent the means ± SEM statistically significant by Student *t* test; ns, *P* > 0.05, *P < 0.05, **P < 0.01. **F**, **I** Data are analyzed using two-way ANOVA, then combined with Tukey to assess the differences between groups. *P < 0.05, **P < 0.01, ***P < 0.001 VS WT Control group. ^$^*P* < 0.05, ^$$^*P* < 0.01, ^$$$^*P* < 0.001

MDD usually occurs with the dysfunction of dopaminergic neurons [40]. We observed in the current study that decreased concentration of DA and its metabolite 3,4-Dihydroxybenzeneacetic acid (DOPAC) were shown in hippocampal brain regions of CUMS depression mouse model, in addition to serotonin-related neurotransmitters 5-HT and 5-hydroxyindoleacetic acid (5-HIAA) (Fig. 3A). However, it has not been fully explained how dopamine concentration levels affect progression of MDD. To further explore the effects of DA on the progression of depression, we analyzed levels of dopamine signaling molecules after establishing depressive mouse model. Results of qRT-PCR confirmed our hypothesis that Drd2 type receptors (Drd2, Drd3 and Drd4) were remarkably up-regulated, while Drd1 type receptors (Drd1 and Drd5) had no significant change (Fig. 3B). Furtherly, only protein level of Drd2 was up-regulated but not Drd1 (Fig. 3C), accompanied with the down-regulated GFAP and decreased β-arrestin2 in the hippocampus of depression-susceptible mice (Additional file 1: Fig. S3G–K). Interestingly, the decreased β-arrestin2 was found to mainly co-localize with GFAP^+^ astrocytes, rather than the Iba-1^+^ microglia by immunofluorescent staining (Fig. 3D). Therefore, we proposed that Drd2 and β-arrestin2 may preferentially participate in the pathogenesis of MDD and the mechanism may lie in regulation of astrocytic functions. We next probed the involvement of Drd2/β-arrestin2 axis in MDD and conducted CUMS model using WT and *Arrb2*^*−/−*^ mice. Neuropathological analysis showed that astrocytic immunofluorescent signaling marked by GFAP was decreased; meanwhile, the shape of astrocytes (GFAP^+^ area) was also shrunken prominently in DG region of hippocampus of WT CUMS mice, implying significant astrocytic loss. *Arrb2*^*−/−*^ aggravated this manifestation of the CUMS-induced depressive mice (Fig. 3E, F and Additional file 1: Fig. S4). Furtherly, deletion of β-arrestin2 also increased secretion of corticosterone (CORT) (Fig. 3H) and cytokines, such as IL-1*β*, IL-6 and TNF-*α* in the hippocampus and plasma of CUMS-induced depressive mice (Fig. 3G, I). These results indicate Drd2 and *β*-arrestin2 participate in the pathogenesis of MDD and genetic deletion of *β*-arrestin2 enlarges the neuroinflammatory signs and astrocytic loss induced by CUMS mouse model.

### β-Arrestin2 knockout aggravates the apoptosis of astrocytes and JAK–STAT3 pathway activation

**Fig. 4:**
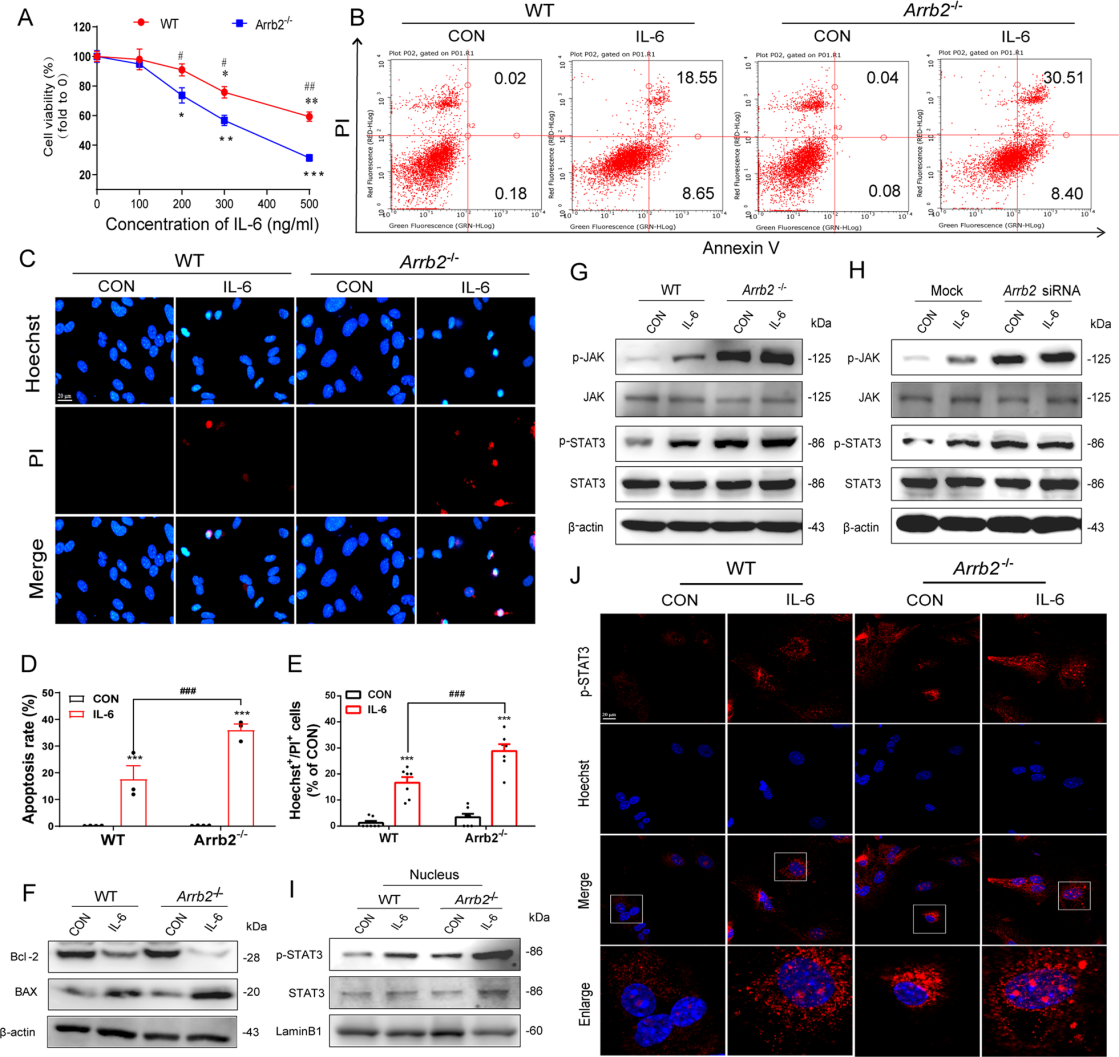
Depletion of β-arrestin2 aggravates apoptosis of astrocytes stimulated by IL-6. **A** Cell viability was detected treated with different concentration of IL-6 (0, 100, 200, 300, 400, 500 ng/mL) for primary astrocytes. **B**, **D** Apoptosis rate of WT and *Arrb2*^*−/−*^ astrocytes were detected by flow cytometry (*n* = 3). **C**, **E** PI/Hoechst staining to observe cell apoptosis stimulated by IL-6 (300 ng/ml). Scale bar: 20 μm *n* = 7. **F** Protein levels of Bcl-2 and BAX were detected in WT and *Arrb2*^*−/−*^ astrocytes. **G**, **H** Protein levels of p-JAK/JAK and p-STAT3/STAT3 were detected in WT and *Arrb2*^*−/−*^ astrocytes or transfected with Arrb2 siRNA. **I** After extraction of nucleus protein from astrocytes, the levels of p-STAT3/STAT3 were detected. **J** Immunofluorescence staining p-STAT3 in WT and *Arrb2*^*−/−*^ astrocytes. p-STAT3: Red; Hoechst: Blue; Scale bar: 20 μm. **A** Bars and error flags represent the means ± SEM of at least three independent experiments; statistically significant by Student *t* test; *P < 0.05, **P < 0.01, ***P < 0.001 VS WT Control group or *Arrb2*^*−/−*^ untreated group. ^#^*P* < 0.05, ^##^*P* < 0.01 VS corresponding IL-6 stimulated group. **D**, **E** Data were denoted as mean ± SEM using two-way ANOVA, then combined with Tukey to assess the differences between groups. ***P < 0.001 VS WT Control group; ^###^*P* < 0.001

Multiple researches have highlighted IL-6 as the most predominant cytokine inducible upon stress and it plays a cardinal role in psychiatric disorders [26, 41]. We observed in our study that IL-6 was significantly increased in the plasma of CUMS mouse model. Therefore, we stimulated the primary astrocytes with IL-6 to mimic the inflammatory microenvironment in brain of depressive mice. Different concentrations of IL-6 (0, 100, 200, 300, 500 ng/mL) were used to treat the astrocytes and we demonstrated that cell viability of astrocytes from WT mice was significantly decreased by 24.20 ± 5.362% and 40.77 ± 4.693% stimulated with IL-6 at 300 and 500 ng/Ml, respectively, while astrocytes from *Arrb2*^*−/−*^ mice showed statistically significant decrease by 26.33 ± 6.449%, 46.59 ± 6.991% and 68.63 ± 4.568% at 200, 300 and 500 ng/mL, respectively (Fig. 4A). As IL-6 with 300 ng/mL induced significant decrease of cell viability in astrocytes from both genotypes, we then selected this concentration for further study. After IL-6 stimulation, the apoptotic rate of WT astrocytes increased by 17.71 ± 6.96%, while β-arrestin2-deficient astrocytes increased by 36.10 ± 3.10% by flow cytometry analysis (Fig. 4B, D). PI/Hoechst staining was used to label dying cells out of total cells, in which β-arrestin2 deletion not only decreased the total cell numbers (Hoechst^+^ cells) but also promoted apoptotic astrocytes (PI^+^ cells) induced by IL-6 (Fig. 4C, E). The expression of apoptosis-related proteins was also detected and we observed increased pro-apoptotic protein Bax and decreased anti-apoptotic protein Bcl-2 after IL-6 treatment (Fig. 4F). This phenomenon was accentuated by β-arrestin2 knockout (Fig. 4F).

CORT, resulting from a dysregulated hypothalamic–pituitary–adrenal (HPA) axis, is associated dysfunctional neuropathological phenotypes including neuroinflammation and behavioral changes of depression [42, 43]. It was shown significantly increased levels of corticosterone in CUMS model in our research as well. Therefore, we used corticosterone-induced inflammation model to verify the effect of β-arrestin2 on astrocytic dysfunctions. Cell viability assay indicated that CORT at 1000 and 1200 μM induced loss of cell activity by 9.562 ± 3.93% and 22.59 ± 8.384%, respectively, while *Arrb2*^*−/−*^ astrocytes were significantly decreased by 16.52 ± 4.950%, 21.08 ± 4.468%, 27.31 ± 4.335%, 43.49 ± 6.315% and 81.10 ± 7.846% in the concentration of 200, 500, 800, 1000 and 1200 μM, respectively (Additional file 1: Fig. S5A). CORT also elevated mRNA levels of TNF-α, IL-6 in WT astrocytes, while β-arrestin2 deficiency aggravated this trend (Additional file 1: Fig. S5B–F). Consistently, levels of the inflammatory cytokines including TNF-α, IL-6, IL-10, IL-18 and IL-1β in the cell supernatant also increased significantly in the *Arrb2*^*−/−*^ astrocytes compared with that of WT astrocytes (Additional file 1: Fig. S5G–K). All these results indicated β-arrestin2 deficiency exacerbated inflammation and loss of astrocytes induced by both IL-6 and CORT.

As is recognized before, IL-6 directly targets the JAK/STAT3 signals and activates the expression of STAT3-targeted genes [44], which process is reported to highly depends on the cGAS–STING pathway [45], that we observed an activated state in isolated astrocytes from susceptible mice of depression model from our results. We, therefore, speculated that regulatory roles of β-arrestin2 in IL-6-induced astrocytic dysfunctions may be involved in JAK/STAT3 signaling pathways. To probe this, we detected molecules in JAK/STAT3 signaling pathways. It was shown that β-arrestin2 knockdown (by siRNA) and β-arrestin2 knockout aggravated the increased phosphorylation of JAK and STAT3 induced by IL-6 (Fig. 4G, H). Phosphorylated JAK transduced the cellular signals to STAT3 and induce the phosphorylation of STAT3 and subsequent nucleic translocation to activate the inflammatory gene transcription [46]. Thus, the nuclei of astrocytes were separated to investigate p-STAT3 transport from cytoplasm to nucleus. β-Arrestin2 deletion aggravated phosphorylation of STAT3 in the nucleus stimulated by IL-6 (Fig. 4I). Immunofluorescence result also verified this conclusion (Fig. 4J). These results indicate that β-arrestin2 knockout aggravates the apoptosis of astrocytes and activation of JAK–STAT3 pathway.

### UNC9995 enhances the interaction of β-arrestin2 and STAT3

**Fig. 5:**
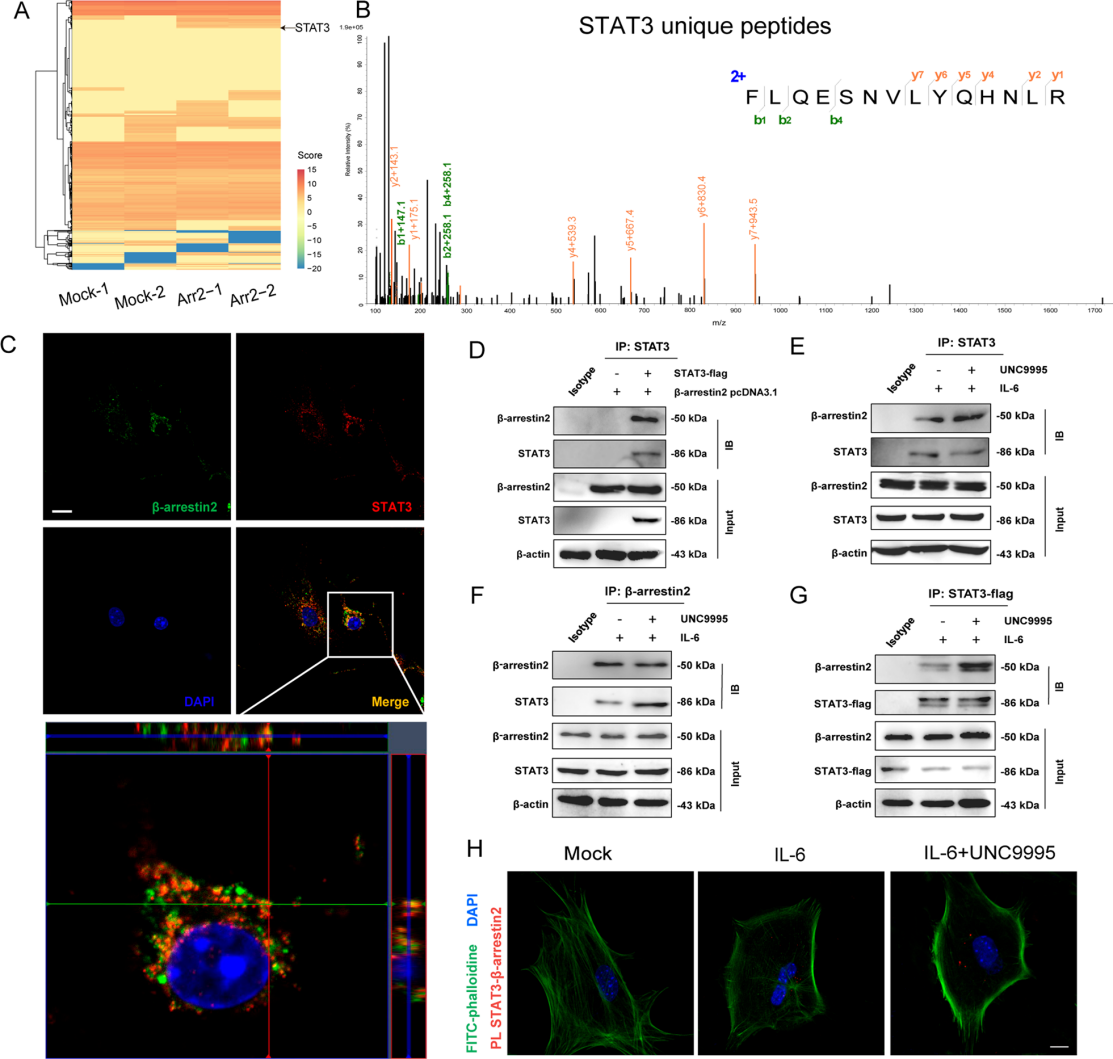
Effect of Drd2 activation on the interaction of β-arrestin2 and NLRP3. **A** Heatmap of 4D label-free mass spectrometry-quantified proteins that bound β-arrestin2 (*n* = 2). **B** Representative fragmentation spectrum of FLQESNVLYQHNLR in STAT3. Flag-tagged STAT3 construct and β-arrestin2 pcDNA3.1 construct was co-transfected in HEK293T cells. **C** Immunofluorescent staining for β-arrestin2, DAPI, and STAT3 in HEK293T cells. Green: β-arrestin2; Red: STAT3; Blue: DAPI. Scale bar: 20 μm. **D** Cell lysates were immunoprecipitated with anti-Flag antibody and then the samples were analyzed by immunoblotting. **E** Cell lysates of primary astrocytes treated with IL-6 (300 ng/mL) and UNC9995 or not were immunoprecipitated with anti-STAT3 antibody, and then the samples were analyzed by immunoblotting. **F** Cell lysates of primary astrocytes treated with IL-6 (300 ng/mL) and UNC9995 or not were immunoprecipitated with anti-β-arrestin2 antibody, and then the samples were analyzed by immunoblotting. **G** Flag-tagged STAT3 construct was transfected in primary astrocytes. Cell lysates of primary astrocytes treated with IL-6 (300 ng/mL) and UNC9995 or not were immunoprecipitated with anti-flag antibody, and then the samples were analyzed by immunoblotting. **H** Primary astrocytes were pretreated with UNC9995 for 1 h and then stimulated with IL-6 (300 ng/mL) for 24 h. β-Arrestin2 and STAT3 proximity ligation signals. Green: FITC–phalloidin; Red: STAT3–β-arrestin2; Blue: DAPI. Scale bar: 20 μm

As a scaffold protein, β-arrestin2 binds to multiple proteins to transduce the cellular signals [47]. It was also shown in our previous study that β-arrestin2 interacts with inflammasome component to suppress its activation [48]. We are inspired to expand β-arrestin2’s function as a scaffold protein in the current research. We, therefore, performed immunoprecipitation using β-arrestin2 antibody and analyzed the proteins bound to β-arrestin2 by mass spectrometry (Additional file 1: Fig. S6A, B). Results from label-free mass spectrometry showed that STAT3 was the direct binding target of β-arrestin2 in the JAK/STAT3 pathway (Fig. 5A, B and Additional file 1: Fig. S6C). We further verified this combination by multiple methods including immunofluorescent staining and co-immunoprecipitation. As shown in Fig. 5C, D, after the plasmid co-transfection, STAT3 exhibited colocalization (Fig. 5C) and was immunoprecipitated with β-arrestin2 (Fig. 5D) within HEK293T cells. These results indicated that β-arrestin2 can combine with STAT3. As we imply previously that UNC9995 is a β-arrestin2-biased agonist and it can recruit the cytosolic β-arrestin2 to boost its scaffolding functions [20], we then used UNC9995 to detect whether recruitment of β-arrestin2 would promote the combination of STAT3 and β-arrestin2. It was observed that UNC9995 (10 μM) promoted the interaction of STAT3 and β-arrestin2 in primary astrocytes by co-immunoprecipitation (Fig. 5E–G). Similarly, results from PLA indicated that UNC9995 treatment facilitated β-arrestin2–STAT3 combination (Fig. 5H). These data suggest that β-arrestin2 interacts with STAT3, which is promoted by β-arrestin2-biased agonist UNC9995.

### UNC9995 abolishes the apoptosis and inflammation of astrocytes in vitro

**Fig. 6:**
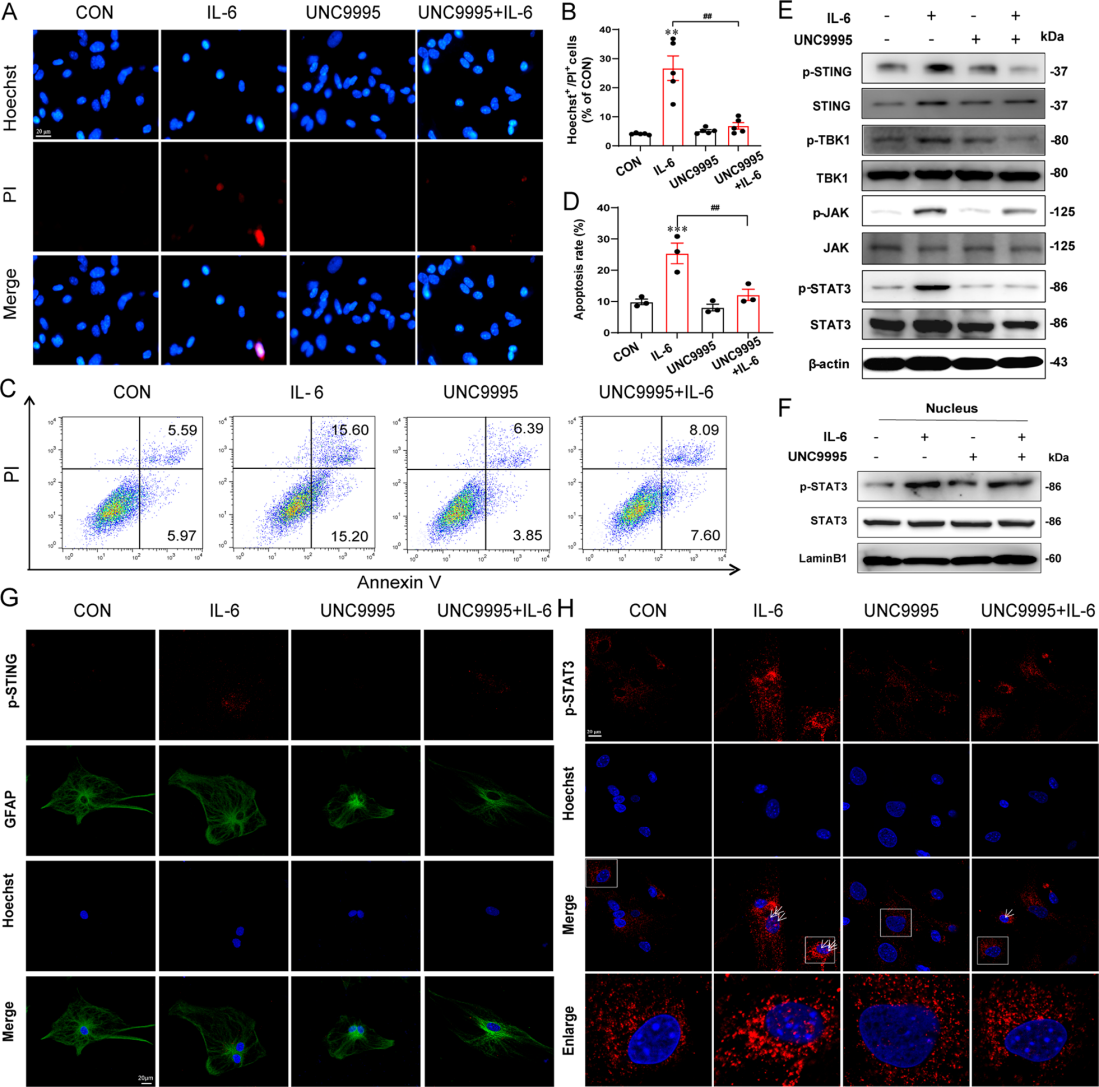
UNC9995 abolishes the apoptosis and inflammation of astrocytes in vitro. **A**, **B** PI/Hoechst staining to observe cell apoptosis pretreated with UNC9995 for 1 h and then stimulated with IL-6 (300 ng/mL) for 24 h. *n* = 5; Scale bar: 20 μm. **C**, **D** Apoptosis rate of astrocytes was detected by flow cytometry which was pretreated with UNC9995 for 1 h and then stimulated with IL-6 (300 ng/mL) for 24 h. *n* = 5. **E** Protein levels of p-STING, p-TBK1, p-JAK, and p-STAT3 were detected in astrocytes stimulated by IL-6 (300 ng/ml) and UNC9995 (10 μM) presence or not. **F** Protein levels of p-STAT3 and STAT3 were detected in the nucleus of astrocytes stimulated by IL-6 (300 ng/ml) and UNC9995 (10 μM) presence or not. **G** Immunofluorescence staining p-STING in astrocytes. p-STING: Red; GFAP: Green; Hoechst: Blue; Scale bar: 20 μm. **H** Immunofluorescence staining p-STAT3 in astrocytes. p-STAT3: Red; Hoechst: Blue; Scale bar: 20 μm. Data are analyzed using one-way ANOVA, then combined with Dunnett’s to assess the differences between groups. ***P < 0.001 VS Control group; ^##^*P* < 0.01, ^###^*P* < 0.001

As we confirmed the significant role of β-arrestin2 in both astrocytic loss and the underlying JAK/STAT3 signaling pathway, combining the promotional effect of UNC9995 on β-arrestin2–STAT3 interaction, we, therefore, probed the potential roles of UNC9995 in astrocytic loss. In the presence of UNC9995, decreased viability of astrocytes induced by IL-6 was increased in a concentration-dependent way, especially above 10 μM (Additional file 1: Fig. S7A). PI/Hoechst staining also showed that the number of apoptotic cells significantly reduced after pretreatment of UNC9995 compared with IL-6 group (Fig. 6A, B). Similarly, After IL-6 stimulation, the apoptotic rate of astrocytes increased by 25.38 ± 4.68% while decreased by 12.11 ± 2.54% in UNC9995 plus IL-6 group indicated by flow cytometry analysis (Fig. 6C, D). In addition, activation of cGAS–STING and JAK/STAT3 pathway stimulated by IL-6 can be abolished by UNC9995, which was reflected by its significantly reversal effects on p-STING, p-TBK1, p-JAK2 and p-STAT3 levels induced by IL-6 (Fig. 6E). Immunofluorescent analysis also showed that UNC9995 abolished the increased level of p-STING in the astrocytes treated with IL-6 (Fig. 6G). Furtherly, we detected the translocation of p-STAT3 to the nucleus by both western blotting and immunofluorescent analysis, which we consistently indicated UNC9995 significantly decreased p-STAT3 levels in the nucleus (Fig. 6F, H). Taken together, UNC9995 abolishes the apoptosis and inflammation of astrocytes, and this effect is accompanied with inactivation of JAK/STAT3 pathway.

### UNC9995 ameliorated depressive-like behaviors and improved the loss of astrocytes in the hippocampus in vivo

**Fig. 7:**
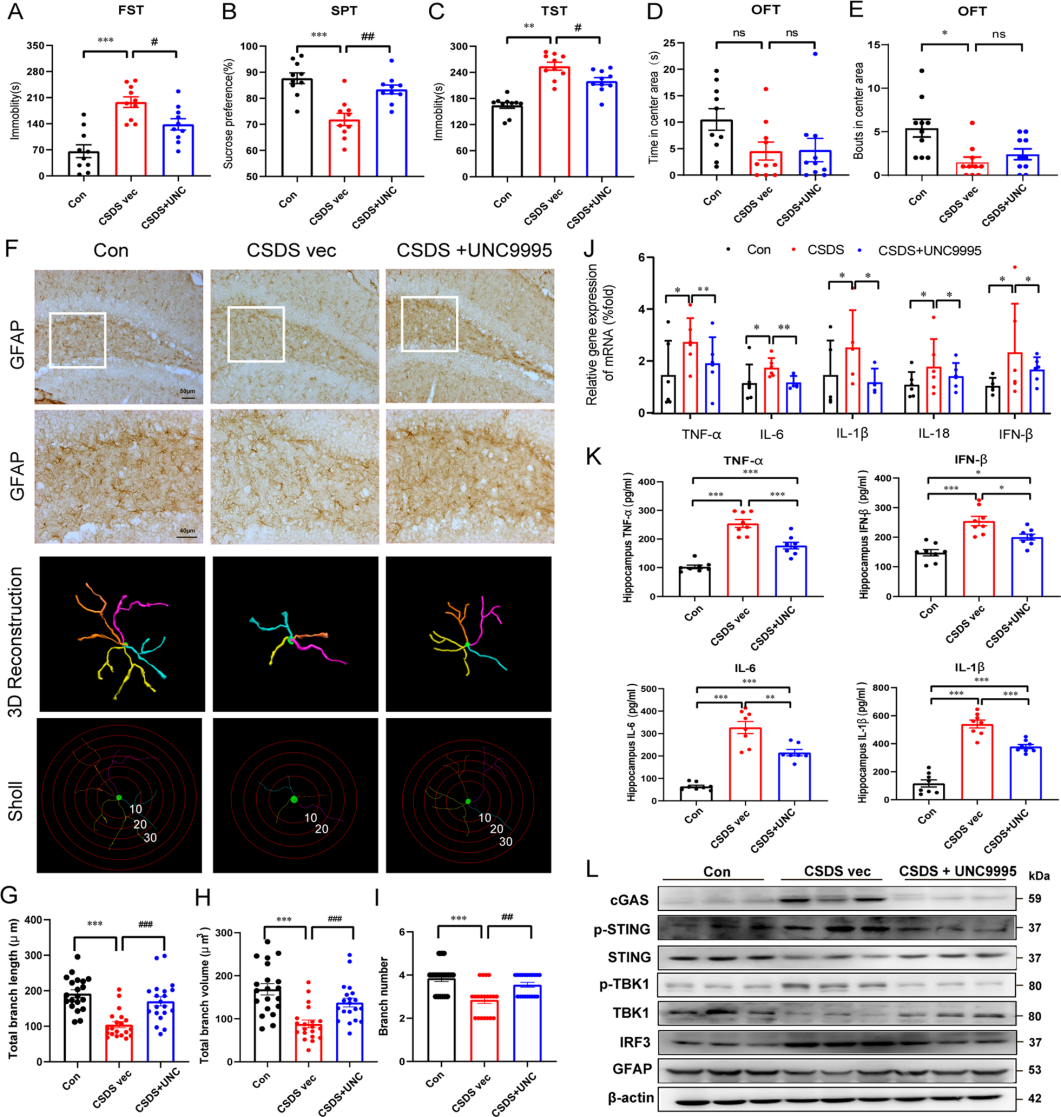
UNC9995 alleviated depressive-like behaviors and improved the loss of astrocytes in the hippocampus. The CSDS model was performed for 2 weeks. Then, susceptible mice were injected with saline or UNC9995 (2 mg/kg/day) for another 10 days. Behavior tests such as FST (**A**), SPT (**B**), TST (**C**), and OFT (**D**, **E**) were conducted. (Control group: ***n = ***10; CSDS group: *n* = 10; UNC9995 group: *n* = 10). **F** Representative images of astrocyte immunostaining for GFAP in the mouse hippocampus, followed by 3D reconstruction and Sholl analysis. Scale bar: 50 mm. Enlarged vision: scale bar: 40 mm. Quantification of average branch length (**G**), total branch volume (**H**), and total branch number (**I**). *n* = 4–6 mice/group, 20 cells/group. The mRNA levels of cytokines such as TNF-*α*, IFN-*β*, IL-6, and IL-1*β* were detected by qRT-PCR (**J**) and ELISA (**K**). **J** *n* = 6; **K** *n* = 8. **L** Protein levels of cGAS, p-STING/STING, p-TBK1/TBK1, IRF3, and GFAP were detected in Control, CSDS, and UNC9995 group. Data are analyzed using one-way ANOVA, then combined with Dunnett’s to assess the differences between groups. ns, *P* > 0.05; ***P < 0.001; ^##^*P* < 0.01, ^###^*P* < 0.001

After confirming the protective roles of UNC9995 in inflammation-induced astrocytic dysfunctions in vivo, we established depressive mouse model to determine its effects in vivo. As shown in Fig. 7A–E, UNC9995 administration decreased the immobility time of mice in the FST and TST tests, increased the sucrose preference in the SPT of CSDS susceptible model mice except for the time in center area and bouts in center area, indicating that UNC9995 partly ameliorated depressive-like behaviors induced by CSDS. From astrocytic pathology, immunohistochemical staining of GFAP, as well as the representative 3D rendering for GFAP^+^ astrocytes (Fig. 7F–I) indicated a significant decrease of total branch length, branch volumes and branch numbers in CSDS-induced depressive mice hippocampus, implying a reduced packing density and decreased ramification of astrocytes, and UNC9995 administration notably rescued this astrocytic atrophy. Meanwhile, inflammatory genes and the cytokines (IL-6, IL-1β, IFN-β and TNF-α) in the hippocampus were detected by qRT-PCR and ELISA. Results indicated that these cytokines were all markedly increased in CSDS model and UNC9995 effectively rescued these cytokines (Fig. 7J, K). As shown in Fig. 7L and Additional file 1: Fig. S7B–H, protein levels of cGAS–STING pathway (cGAS, p-STING, p-TBK1, IRF-3and p-TBK1) were up-regulated significantly in CSDS model, while UNC9995 abolished this increase. These results suggest UNC9995 alleviates depressive-like behaviors and improved the astrocytic atrophy and neuroinflammation in hippocampus in vivo.

### β-Arrestin2 deletion abolishes the antidepressant effects of UNC9995

**Fig. 8:**
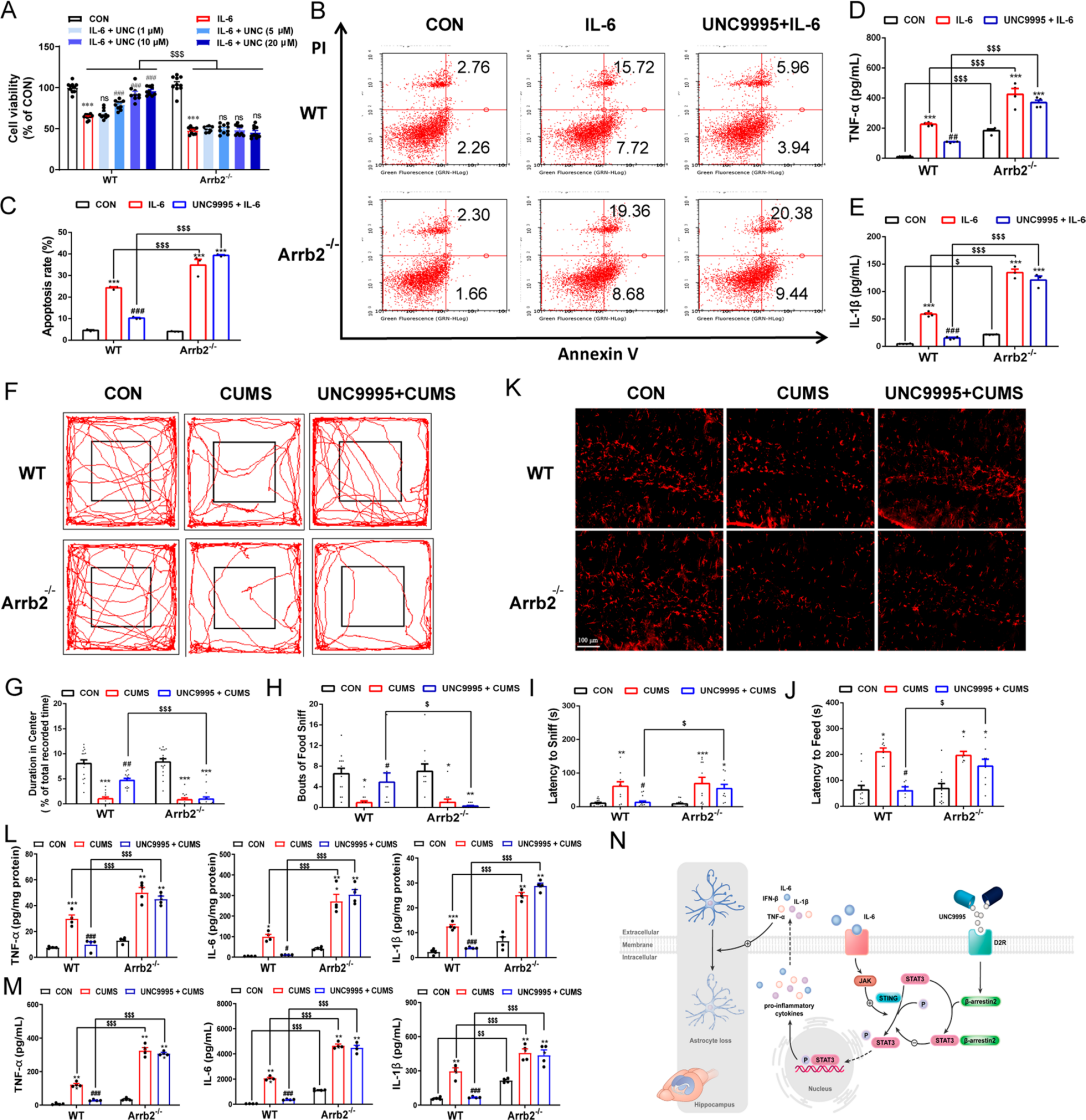
UNC9995 attenuates apoptosis and inflammation of astrocytes and can be canceled by depletion of β-arrestin2. **A** Cell viability of astrocytes pretreated with UNC9995 (1, 5, 10, and 20 μM) for 1 h and then stimulated with IL-6 (300 ng/mL) for 24 h in WT and *Arrb2*^*−/−*^ astrocytes. **B**, **C** Apoptosis rate was detected by flow cytometry which was pretreated with UNC9995 for 1 h and then stimulated with IL-6 (300 ng/mL) for 24 h in WT and *Arrb2*^*−/−*^ astrocytes. Levels of cytokines TNF-*α* (**D**) and IL-1*β* (**E**) were detected by ELISA. WT and *Arrb2*^*−/−*^ mice were made CUMS models. The time in the center area (**F**, **G**), bouts of food sniff (**H**), latency to sniff (**I**), and latency to feed (**J**) in CUMS model mice were detected. **K** Immunofluorescence staining GFAP in the hippocampus of WT and *Arrb2*^*−/−*^ mice. **L**, **M** Cytokines of IL-1*β*, TNF-*α,* and IFN-*β* in the hippocampus and plasma were detected. **N** Schmeichel model of activation of β-arrestin2-biased signaling by UNC9995 in the depression mouse model. Data are analyzed using two-way ANOVA, then combined with Tukey to assess the differences between groups. ns, *P* > 0.05. *P < 0.05, **P < 0.01, ***P < 0.001 VS WT Control group. ^#^*P* < 0.05, ^##^*P* < 0.01, ^###^*P* < 0.001 VS WT IL-6 group or WT CUMS group. ^$^*P* < 0.05, ^$$^*P* < 0.01, ^$$$^*P* < 0.001

We next investigated whether UNC9995 alleviated depressive-like behaviors via a β-arrestin2-dependent pathway. Thus, we detected the effect of UNC9995 in β-arrestin2 deficiency astrocytes. As shown in Fig. 8A, UNC9995 recovered viability of astrocytes induced by IL-6 in a concentration-dependent way, and depletion of β-arrestin2 abolished its effects. In support of this results, flow cytometry of AV/PI showed apoptosis of astrocytes induced by IL-6 in both WT and *Arrb2*^*−/−*^ cells, and pretreatment of UNC9995 rescued the astrocytic apoptosis in WT cells but not *Arrb2*^*−/−*^ cells (Fig. 8B, C). Furthermore, depletion of β-arrestin2 weakened the reversal effects of UNC9995 on IL-1β and TNF-α increase induced by IL-6 (Fig. 8D, E). Moreover, these data were reproduced in corticosterone-induced astrocytic cultures, in which the inhibitory roles of UNC9995 in release of inflammatory cytokines were totally cancelled by β-arrestin2 deletion (Additional file 1: Fig. S8A–E).

In the CUMS-induced depressive mouse model, the administration of UNC9995 improve depressive-like symptoms of CUMS mouse model in WT mice but not *Arrb2*^*−/−*^ mice, reflected by increased time in center area in the OFT (Fig. 8F, G), more bouts of food sniff (Fig. 8H) and decreased latency to sniff and feed in the novel feeding test (Fig. 8I, J). Consistently, significant loss of astrocytes in the hippocampus of CUMS mice was recovered by UNC9995 administration, while these effects were abolished by depletion of β-arrestin2 (Fig. 8K). For the inflammatory cytokines including IL-1β, TNF-α and IL-6 in the hippocampus (Fig. 8L) and plasma (Fig. 8M) also confirmed that β-arrestin2 deletion cancelled the anti-inflammatory effects of UNC9995. Taken together, these results suggest depletion of β-arrestin2 cancels the anti-depressive effects of UNC9995.

## Discussion

Chronic stress induced depressive-like behaviors has been well-established over the past 10 years [49, 50]. During this pathological process, the hippocampus of patients and rodent models showed different degrees of atrophy and reduced expression of astrocytic marker GFAP [51, 52]. Multiple studies have highlighted the pathological changes of astrocytes in the hippocampal DG area as important phenotypic markers as well as biological mechanisms of depressive progression and pharmacotherapy [53, 54]. However, the evidence to abundantly understand the pathological changes of astrocytes is still lacking. In this study, we isolate astrocytes from the brain of depressive mice and conduct RNA sequencing to trace the genetic profiles of astrocytes. We demonstrate that inflammatory genes are increased and inflammatory pathways are activated in astrocytes, accompanied with the hippocampal changes of dopamine D2 receptor and β-arrestin2. As the decreased β-arrestin2 coincidentally appears in atrophic astrocytes, we, therefore, propose that β-arrestin2 is involved in the neuroinflammation and astrocytic dysfunctions in MDD. From the follow-up verification, β-arrestin2 deletion aggravates depressive symptoms, neuroinflammatory responses and astrocytic atrophy. As to the mechanism, in vitro studies show that astrocytic β-arrestin2 combines with STAT3 in the cytosol, which inhibits its phosphorylation and the subsequent translocation to the nucleus. This process is essential for the STAT3-targeted inflammatory activation. With this, we used β-arrestin2-biased Drd2 agonist UNC9995 and find that it promotes the combination of β-arrestin2 and STAT3. Consequently, UNC9995 protects from the inflammation-induced loss of astrocytes and ameliorates depressive-like behaviors in mouse model for depression. Our work shows β-arrestin2 is a potent target of MDD and activation of β-arrestin2-biased signaling by UNC9995 provides prominent effects for treatment strategy of MDD (Fig. 8N).

Dopamine and its receptors serve as crucial factors in neurophysiology and pathology [55], conventionally the Parkinson’s disease [56]. Emerging evidence suggests that disruptions in dopamine system may underlie the pathophysiology of neuropsychiatric disorders, including depression [7]. Indeed, many of the symptoms seen in depression, such as anhedonia and amotivation, have been consistently associated with depression in the dopamine system [7]. Dopamine not only regulates behavior, movement and endocrine functions as a neurotransmitter, but also functions as an important molecule bridging the nervous and immune systems by acting through its receptors on the cytomembrane [57]. Previous results have confirmed the deficiency of DA is tightly associated with CNS inflammation in the neurodegenerative progression [58]. Consist with this, we demonstrate that dopamine receptor knockout mice show remarkable inflammatory response in the brain [16], suggesting that DA deficiency and blockade of dopaminergic downstream signaling disturbs neuroinflammatory homeostasis. In line with this notion, dopamine and dopamine receptor agonist show strong therapeutic efficacy on inflammatory diseases [48, 59]. In this article, after confirming the dysfunctions of dopaminergic systems in depressive mouse model, typically the Drd2/β-arrestin2 pathway, we find that blockade of this pathway by genetic deletion of β-arrestin2 aggravates the inflammatory signals and thus exacerbates depressive-like behaviors and astrocytic loss, while activation of Drd2 using β-arrestin2-biased agonist UNC9995 ameliorates all these manifestations. β-Arrestin2 as one of the arrestins, was initially identified for their role in homologous desensitization and internalization of GPCRs. It is also a scaffolding protein and can also transduce signaling by interacting with other signaling molecules independent of its role of mediating GPCR desensitization [60, 61]. Our previous study has verified the involvement of UNC9995 in G protein- and β-arrestin2-dependent pathways using a Gαi protein inhibitor to raise levels of cyclic AMP, in which we suggest that UNC9995 has no activation in G protein-dependent pathway [20]. We extend the GPCR-independent effects of β-arrestin2 in the progression of MDD in the current research and demonstrate for the first time that Drd2/β-arrestin2-biased signal take a role in the pathogenesis of MDD. Moreover, one of the unprecedented β-arrestin2-biased series chemicals UNC9995 represents a valuable chemical probe for further antidepressant therapy, in which inflammation serve as a bridge. As to the another arrestins family member β-arrestin1, it is reported to show a clear functional divergence with β-arrestin2 due to the differences of these two molecules in protein sequence, conformation as well as cellular localization [62]. Indeed, we have observed the opposing functions of β-arrestin1 and 2 in regulating neuroinflammation of Parkinson’s disease [36]. Here, we showed decreased β-arrestin2 protein level but no significant change of β-arrestin1 after depressive mouse modeling, which also prove the functional specialization for the two isoforms.

Inflammatory responses are so ubiquitous to involve in physiological and pathological processes of the whole body, even the brain that is used to be supposed as an immune exempt organ is no exception [63]. Inflammatory pathways active in peripheral diseases also dominate the nervous system. cGAS is an aberrant DNA sensor, which resists exogenous virus infection via the cGAS/STING signaling cascade by producing a battery of immune and inflammatory mediators, including type I and type III interferons [64]. Although no definite experimental evidence shows the involvement of this signaling pathway in the occurrence and development of MDD, some hints suggests this correlation. Clinical data point out that infection with specific viruses can increase the risk of depression [65]. And the application of interferon in the antiviral treatment also induces clinical symptoms of depression in patients [66]. Besides, mounting evidence has demonstrated that the pathological relevance of cGAS/STING extends far beyond traditional antimicrobial immunity by affecting various non-infectious cellular stress response and stress adaptation programs [64]. With all this basis, we observe increased levels of IFN-β in the depressive mice brain and then discover the pathological activation of cGAS in the hippocampal astrocytes of depressed mice, which is the first experimental evidence to prove the correlation. As to the therapeutic effects of UNC9995, we observe that it recovers the activated cGAS/STING signals. A key question is how the reversal effects of UNC9995 takes? It is worth mentioning that an existing literature indicated that activation of β-arrestin2 downstream of β1-adrenergic receptors can directly bind cGAS and promote the activation of this signaling pathway [67], while our results pointed out the role of astrocytic Drd2/β-arrestin2 in negatively regulating cGAS. This inconsistency may be caused by different β-arrestin2-biased G-protein receptors. The focus of this paper does not fully clarify these two issues, and we still need to invest in further research.

Inflammation represents cellular defense against stress, which is closely related to the stability of the nervous system. It turns out that chronic low-level inflammation exists in the development of nerve cells and cause imperceptible but accumulatively devastating changes, while acute high-level inflammatory stress directly induces nerve cell dysfunctions or death [68]. Combining with our previous findings, we put forward a notion in this article that β-arrestin2-biased Drd2 signaling in astrocytes suppress the inflammatory damage caused by cGAS activation due to depressive stress, and UNC9995 treatment can significantly moderate astrocytes inflammatory process and ameliorate depressive behavior (Additional file 2).

## Conclusions

In this article, we suggest an innate immune signaling-cGAS/STING can be activated by depressive stress, and its activation contributes to hippocampus astrocytes impairment during disease process. UNC9995 serve as Drd2/β-arrestin2-biased agonist, and it suppresses cGAS/STING activation by interacting with JAK/STAT3 signaling and relieves depressive behavior in CSDS model. Thus, these findings demonstrate Drd2/β-arrestin2 signaling pathway is a potential therapeutic target for depression and UNC9995 provides a new insight for the therapy of depression.

## Supplementary Information

**Additional file 1: Fig. S1.** CUMS depression model induces astrocyte dysfunction in the hippocampus of mice. (**A**) Astrocyte dysfunction induced by CUMS. Representative images of astrocyte immunostaining for GFAP in mice hippocampus, Scale bar: 200 μm (upper panel); Scale bar: 40 μm (lower panel). (**B**) Immunofluorescence staining of NeuN in mice hippocampus. NeuN: Red; DAPI: Blue; Scale bar: 100 μm. (**C**) Microglial activation induced in CUMS model. Representative images of microglial immunostaining for Iba-1 in mice hippocampus, Scale bar: 100 μm. (**D**) The protein level of GFAP was detected in the hippocampus of mice in CUMS model. (**E**) Immunoblot analysis and quantification of GFAP (n = 3). (**F**) Relative mRNA expression of IL-1α, IL-1β, IL-6, and TNF-α in the hippocampus of mice in CUMS model (n = 3). Data were presented as mean ± SEM; statistically significant by Student t-test; *P < 0.05, **P < 0.01, ***P < 0.001. **Fig. S2.** RNA sequencing performed in the astrocytes isolated from the hippocampus of WT and CSDS susceptible mice. (**A**) The density of genes compared with WT and CSDS susceptible group. (**B**) Differentially up-regulated and down-regulated expressed genes compared with WT and CSDS susceptible group. (**C**) Statistics of AS events in WT and CSDS susceptible group. (**D**) Pearson correlation analysis between WT and CSDS susceptible group. (**E**) PCA plot analysis. (**F**) The exon, intro and intergenic between WT and CSDS susceptible group. **Fig. S3.** Immunoblot quantification of protein level in the hippocampus of control, susceptible and resilient mice. Immunoblot quantification analysis of cGAS (**A**), STING (**B**), p-STING (**C**), TBK1(**D**), p-TBK1 (**E**), IRF3 (**F**), Drd1(**G**), Drd2 (**H**), β-arrestin1 (**I**), β-arrestin2 (**J**) and GFAP (**K**) in the control, susceptible and resilient mice group. Data are analyzed using one-way ANOVA, then combined with Dunnett's to assess the differences between groups. ns, *P* > 0.05, *P < 0.05, **P < 0.01, ***P < 0.001 VS Control group; ns, *P* > 0.05, *P < 0.05, **P < 0.01, ***P < 0.001 VS CSDS Susceptible group. **Fig. S4.** CUMS induces loss of astrocyte and downregulation of β-arrestin2. (**A**) Immunofluorescence staining of S100β and β-arrestin2 in mice hippocampus by CUMS. β-arrestin2: Red; S100β: Green; DAPI: Blue; Scale bar: 100 μm. (**B**–**C**) Immunoblot analysis and quantification of β-arrestin2 in WT and CUMS group (n = 3). Data were presented as mean ± SEM; statistically significant by Student t-test; *P < 0.05. **Fig. S5.** β-arrestin2 deficiency exacerbated corticosterone-induced astrocytes inflammation. (**A**) Cell viability was detected while treated with different concentrations of corticosterone (0, 200, 400, 600, 800, 1000, and 1200 μM) in primary WT and Arrb2−/−astrocytes. Bars and error flags represent the means ± SEM of at least three independent experiments; statistically significant by Student t-test; *P < 0.05, **P < 0.01, ***P < 0.001 VS WT Control group or Arrb2−/− untreated group. #*P* < 0.05, ##*P* < 0.01VS corresponding CORT stimulated group. (**B**–**E**) The mRNA level of TNF-α, IL-6, IL-10, IL-1β, and IL-18 in primary WT and Arrb2−/− astrocytes induced by corticosterone (n = 6). (**G**–**K**) The Cytokines of TNF-α, IL-6, IL-10, IL-1β, and IL-18 in the supernatant from primary WT and Arrb2−/− astrocytes while treated with corticosterone (n = 6). Data are analyzed using two-way ANOVA, then combined with Tukey to assess the differences between groups. ns, *P* >0.05, *P < 0.05, **P < 0.01, ***P < 0.001 VS WT Control group. ns, *P* >0.05, #*P* < 0.05, ##*P* <0.01, ###*P* < 0.001 VS WT CORT group. ns, *P* > 0.05, $*P* < 0.05, $$*P* < 0.01, $$$ *P* < 0.001 VS Arrb2−/− group. **Fig. S6.** Mass spectrometry quantification of proteins that bind to β-arrestin2. (**A**) Coomassie dye after SDS-PAGE. (**B**) Schematic summarizing of mass spectrometry quantification. (**C**) The base peak diagram of the tested samples (Mock-1, Mock-2, Arrb2-1, Arrb2-2). **Fig. S7.** Immunoblot quantification of protein level in the hippocampus of Control, CSDS and CSDS mice treatment with UNC9995. (**A**) Cell viability of astrocytes pretreated with UNC9995 (1, 5, 10, 20 μM) for 1h and then stimulated with IL-6 (300 ng/mL) for 24 h in astrocytes. n = 3. Immunoblot quantification analysis of cGAS (**B**), STING (**C**), p-STING (**D**), TBK1 (**E**), p-TBK1 (**F**), IFN-β (**G**) and GFAP (**H**) in the hippocampus of control, CSDS vehicle, and CSDS mice treatment with UNC9995. Data were presented as mean ± SEM; statistically significant by Student t-test; (**A**) **P < 0.01 VS control, #*P* <0.01, ###*P* < 0.001 VS IL-6 group. (**B**–**H**) Data are analyzed using one-way ANOVA, then combined with Dunnett's to assess the differences between groups. ns, *P* > 0.05, *P < 0.05, **P < 0.01, ***P < 0.001 VS Control group; ns, *P* > 0.05, *P < 0.05, **P < 0.01, ***P < 0.001 VS CSDS Susceptible group.**Fig. S8.** UNC9995 abolished the corticosterone-induced astrocytes inflammation which can be exacerbated by β-arrestin2 knockout. (**A**–**E**) The Cytokines of TNF-α, IL-6, IL-10, IL-1β, and IL-18 were detected in the supernatant from primary WT and Arrb2−/− astrocytes induced by corticosterone pretreatment with UNC9995 (10 μM). n = 6. Data are analyzed using two-way ANOVA, then combined with Tukey to assess the differences between groups. ns, *P* > 0.05, *P < 0.05, **P < 0.01, ***P < 0.001 VS WT Control group. ns, *P* > 0.05, #*P* < 0.05, ###*P* < 0.001 VS WT CORT group. ns, *P* > 0.05, $*P* < 0.05, $$$*P* < 0.001 VS WT CORT+UNC9995 group. **Supplementary Table 1.** Primers used in the study.

**Additional file 2:** The original data of Western blot in the study.

## Data Availability

All data generated or analyzed during this study are included in this published article and its Additional file 1: Figs. S1–S8 and Table S1.

## Abbreviations

SSRIs: Selective serotonin reuptake inhibitors
CSDS: Chronic social defeated stress
CUMS: Chronic unpredictable mild stress
MDD: Major depressive disorder
GPCRs: G-protein-coupled receptors
IL-1β: Interleukin-1β
IL-6: Interleukin-6
TNF-α: Tumor necrosis factor-α
DG: Dentate gyrus
FST: Forced swim test
SPT: Sucrose preference test
TST: Tail suspension test
