# Supplementary material for: β-Arrestin2-biased Drd2 agonist UNC9995 alleviates astrocyte inflammatory injury via interaction between β-arrestin2 and STAT3 in mouse model of depression
Source: J Neuroinflammation. 2022 Oct 1;19:240. doi: 10.1186/s12974-022-02597-6 (PMC9526944; doi:10.1186/s12974-022-02597-6)
Supplement: Supplementary file 1 — Additional file 1: Fig. S1. CUMS depression model induces astrocyte dysfunction in the hippocampus of mice. (A) Astrocyte dysfunction induced by CUMS. Representative images of astrocyte immunostaining for GFAP in mice hippocampus, Scale bar: 200 μm (upper panel); Scale bar: 40 μm (lower panel). (B) Immunofluorescence staining of NeuN in mice hippocampus. NeuN: Red; DAPI: Blue; Scale bar: 100 μm. (C) Microglial activation induced in CUMS model. Representative images of microglial immunostaining for Iba-1 in mice hippocampus, Scale bar: 100 μm. (D) The protein level of GFAP was detected in the hippocampus of mice in CUMS model. (E) Immunoblot analysis and quantification of GFAP (n = 3). (F) Relative mRNA expression of IL-1α, IL-1β, IL-6, and TNF-α in the hippocampus of mice in CUMS model (n = 3). Data were presented as mean ± SEM; statistically significant by Student t-test; *P < 0.05, **P < 0.01, ***P < 0.001. Fig. S2. RNA sequencing performed in the astrocytes isolated from the hippocampus of WT and CSDS susceptible mice. (A) The density of genes compared with WT and CSDS susceptible group. (B) Differentially up-regulated and down-regulated expressed genes compared with WT and CSDS susceptible group. (C) Statistics of AS events in WT and CSDS susceptible group. (D) Pearson correlation analysis between WT and CSDS susceptible group. (E) PCA plot analysis. (F) The exon, intro and intergenic between WT and CSDS susceptible group. Fig. S3. Immunoblot quantification of protein level in the hippocampus of control, susceptible and resilient mice. Immunoblot quantification analysis of cGAS (A), STING (B), p-STING (C), TBK1(D), p-TBK1 (E), IRF3 (F), Drd1(G), Drd2 (H), β-arrestin1 (I), β-arrestin2 (J) and GFAP (K) in the control, susceptible and resilient mice group. Data are analyzed using one-way ANOVA, then combined with Dunnett's to assess the differences between groups. ns, P > 0.05, *P < 0.05, **P < 0.01, ***P < 0.001 VS Control group; ns, P > 0.05, *P < 0.05, **P < 0.0 [file 12974_2022_2597_MOESM1_ESM.docx]

**Supplementary Materials**

**β-arrestin2-biased Drd2 agonist UNC9995 alleviates astrocyte inflammatory injury via interaction between β-arrestin2 and STAT3 in mouse model of depression**

Yang Liu^a#^, Nanshan Song^a#^, Hang Yao^b #^, Siyuan Jiang^b^, Yueping Wang^b^, Ying Zheng^a^, Yuanzhang Zhou^b^, Jianhua Ding^b^, Gang Hu ^a, b *^, Ming Lu^b,c *^

*^a^Department of Pharmacology, Nanjing University of Chinese Medicine, Nanjing 210023, China*

*^b^Jiangsu Key Laboratory of Neurodegeneration, Department of Pharmacology, Nanjing Medical University, Nanjing 211166, China*

*^c^Neuroprotective Drug Discovery Key Laboratory, Department of Pharmacology, Nanjing Medical University, Nanjing 211166, China*

**Supplementary figures**


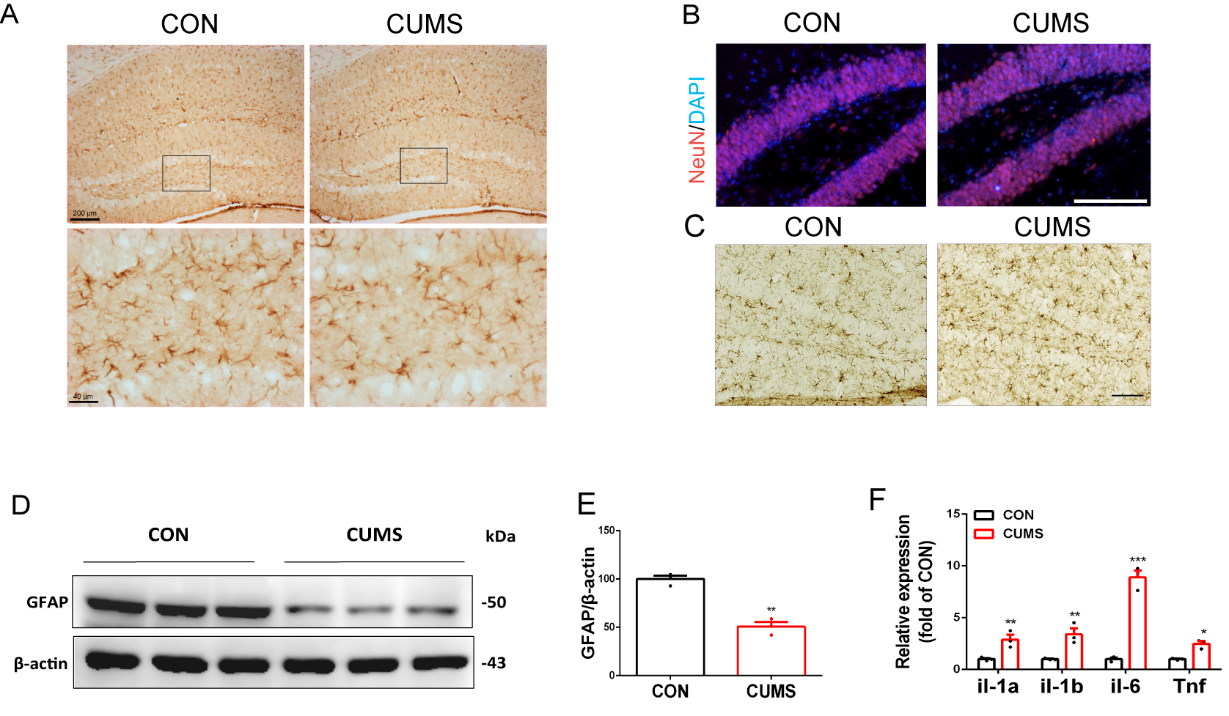


**Fig. S1. CUMS depression model induces astrocyte dysfunction in the hippocampus of mice.** (A) Astrocyte dysfunction induced by CUMS. Representative images of astrocyte immunostaining for GFAP in mice hippocampus, Scale bar: 200 μm (upper panel); Scale bar: 40 μm (lower panel). (B) Immunofluorescence staining of NeuN in mice hippocampus. NeuN: Red; DAPI: Blue; Scale bar: 100 μm. (C) Microglial activation induced in CUMS model. Representative images of microglial immunostaining for Iba-1 in mice hippocampus, Scale bar: 100 μm. (D) The protein level of GFAP was detected in the hippocampus of mice in CUMS model. (E) Immunoblot analysis and quantification of GFAP (n=3). (F) Relative mRNA expression of IL-1*α*, IL-1*β*, IL-6, and TNF-*α* in the hippocampus of mice in CUMS model (n=3). Data were presented as mean ± SEM; statistically significant by Student t-test; **P* < 0.05, ***P* < 0.01, ****P* < 0.001.


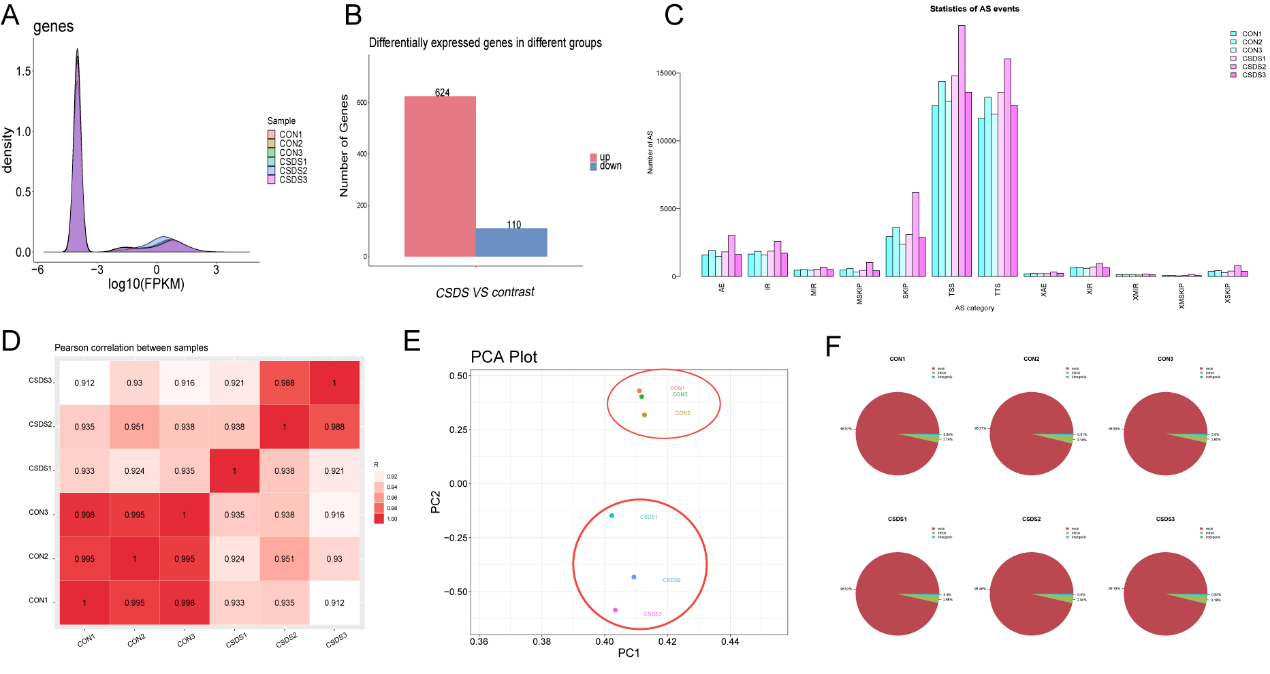


**Fig. S2. RNA sequencing performed in the astrocytes isolated from the hippocampus of WT and CSDS susceptible mice.** (A) The density of genes compared with WT and CSDS susceptible group. (B) Differentially up-regulated and down-regulated expressed genes compared with WT and CSDS susceptible group. (C) Statistics of AS events in WT and CSDS susceptible group. (D) Pearson correlation analysis between WT and CSDS susceptible group. (E) PCA plot analysis. (F) The exon, intro and intergenic between WT and CSDS susceptible group.


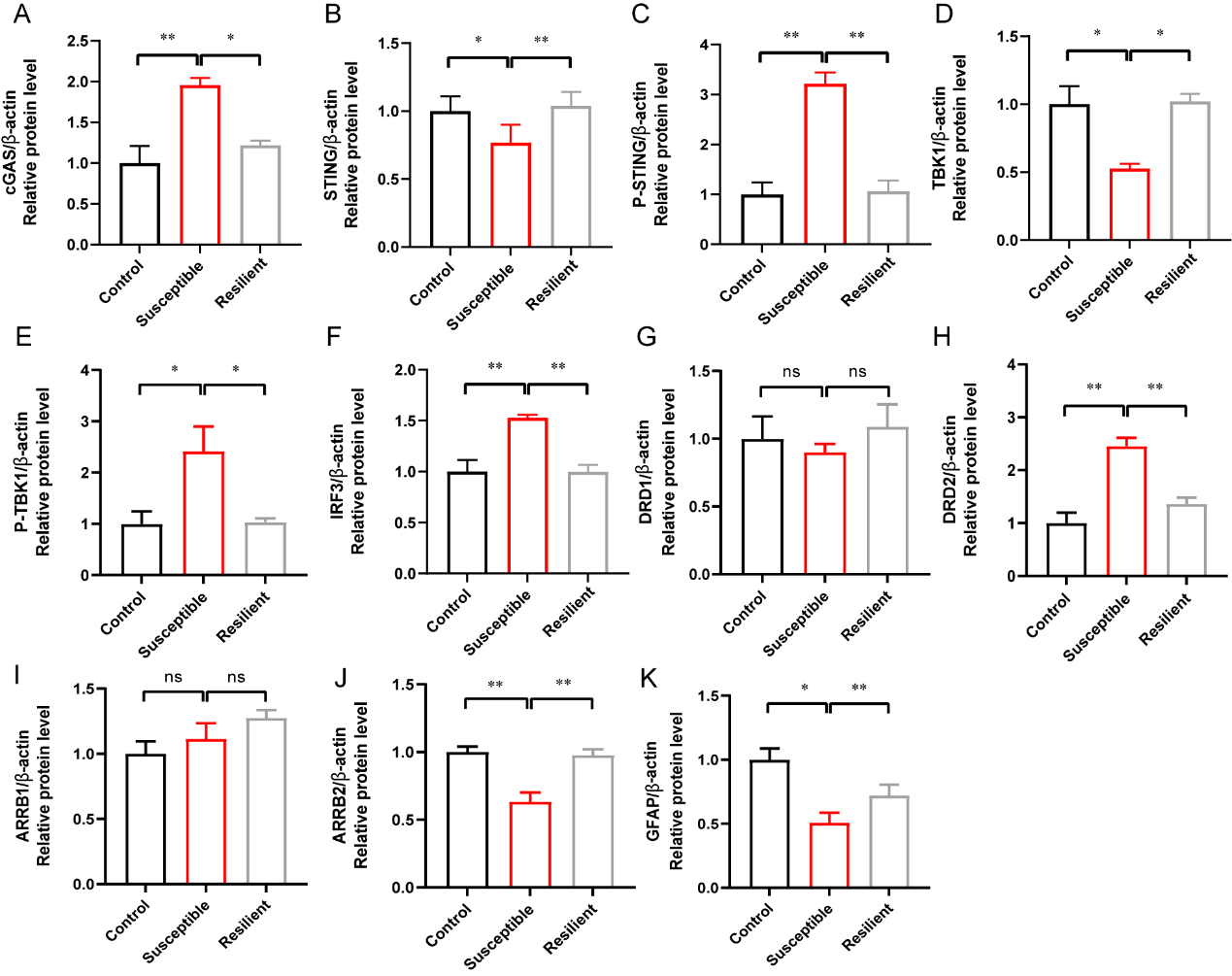


**Fig. S3. Immunoblot quantification of protein level in the hippocampus of control, susceptible and resilient mice.** Immunoblot quantification analysis of cGAS (A), STING (B), p-STING (C), TBK1(D), p-TBK1 (E), IRF3 (F), Drd1(G), Drd2 (H), *β*-arrestin1 (I), *β*-arrestin2 (J) and GFAP (K) in the control, susceptible and resilient mice group. Data are analyzed using one-way ANOVA, then combined with Dunnett's to assess the differences between groups. ns, *P*>0.05, **P*<0.05, ***P*<0.01, ****P*<0.001 VS Control group; ns, *P*>0.05, **P*<0.05, ***P*<0.01, ****P*<0.001 VS CSDS Susceptible group.


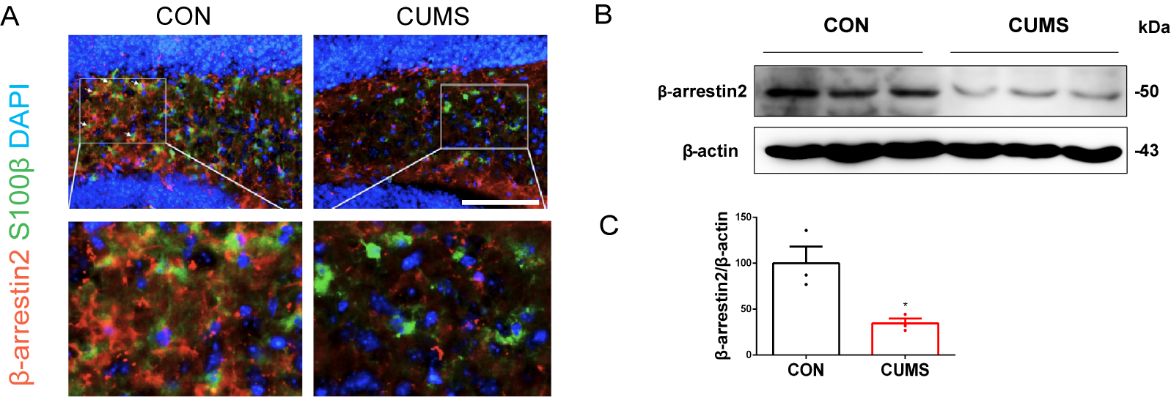


**Fig. S4. CUMS induces loss of astrocyte and downregulation of β-arrestin2.** (A) Immunofluorescence staining of S100*β* and *β*-arrestin2 in mice hippocampus by CUMS. *β*-arrestin2: Red; S100*β*: Green; DAPI: Blue; Scale bar: 100 μm. (B-C) Immunoblot analysis and quantification of *β*-arrestin2 in WT and CUMS group (n=3). Data were presented as mean ± SEM; statistically significant by Student t-test; **P* < 0.05.


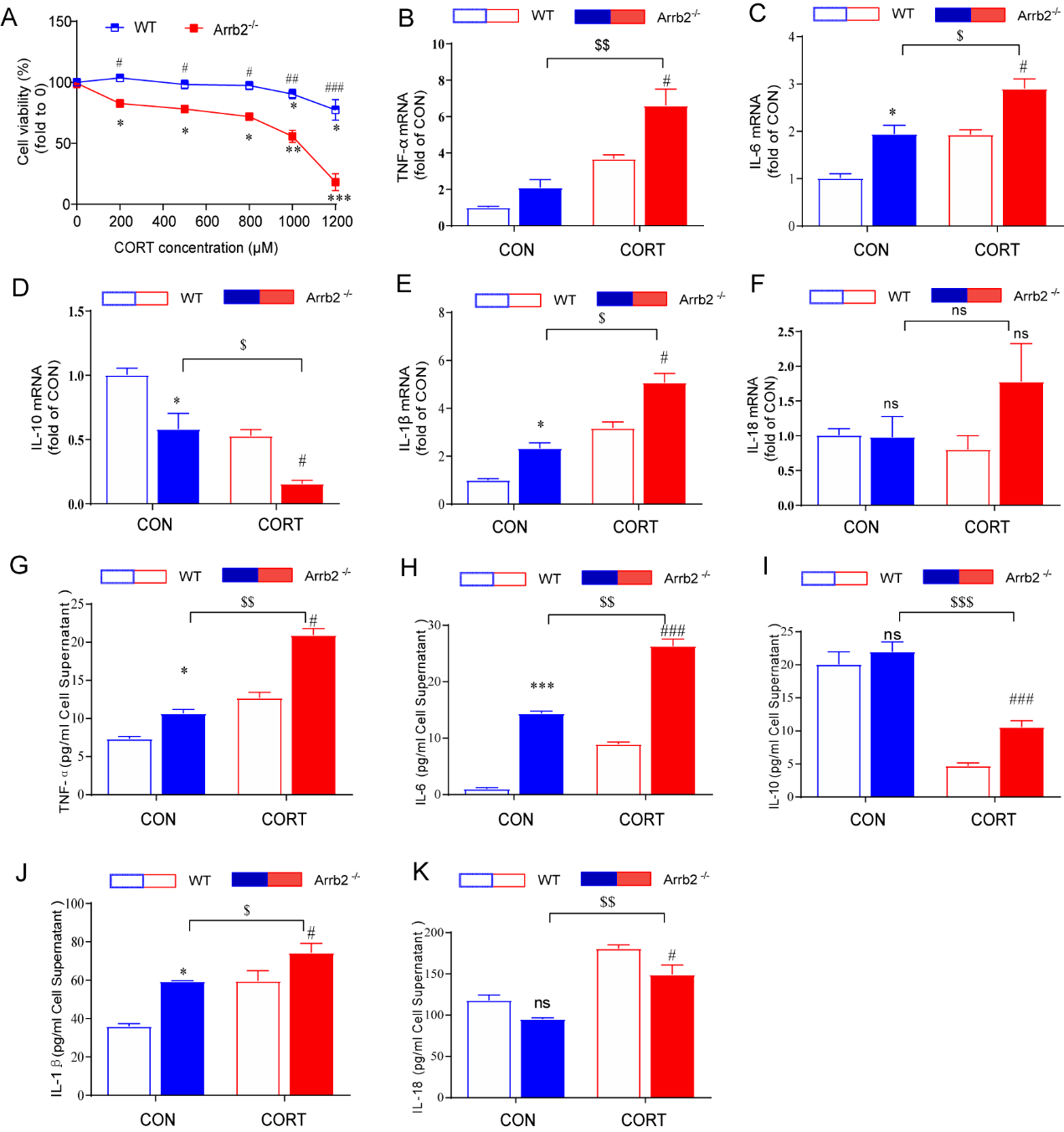


**Fig. S5.** **β-arrestin2 deficiency exacerbated corticosterone-induced astrocytes inflammation.** (A) Cell viability was detected while treated with different concentrations of corticosterone (0, 200, 400, 600, 800, 1000, and 1200 μM) in primary WT and *Arrb2^-/-^*astrocytes. Bars and error flags represent the means ± SEM of at least three independent experiments; statistically significant by Student t-test; **P*<0.05, ***P*<0.01, ****P*<0.001 VS WT Control group or *Arrb2^-/-^* untreated group. ^#^*P*<0.05, ^##^*P*<0.01VS corresponding CORT stimulated group. (B-E) The mRNA level of TNF-*α*, IL-6, IL-10, IL-1*β*, and IL-18 in primary WT and *Arrb2^-/-^* astrocytes induced by corticosterone (n=6). (G-K) The Cytokines of TNF-*α*, IL-6, IL-10, IL-1*β*, and IL-18 in the supernatant from primary WT and *Arrb2^-/-^* astrocytes while treated with corticosterone (n=6). Data are analyzed using two-way ANOVA, then combined with Tukey to assess the differences between groups. ns, *P* >0.05, **P* < 0.05, ***P* < 0.01, ****P* < 0.001 VS WT Control group. ns, *P* >0.05, ^#^ *P* < 0.05, ^##^*P* <0.01, ^###^*P* < 0.001 VS WT CORT group. ns, *P* >0.05, ^$^ *P* < 0.05, ^$ $^ *P* <0.01, ^$ $ $^ *P* < 0.001 VS *Arrb2^-/-^* group.


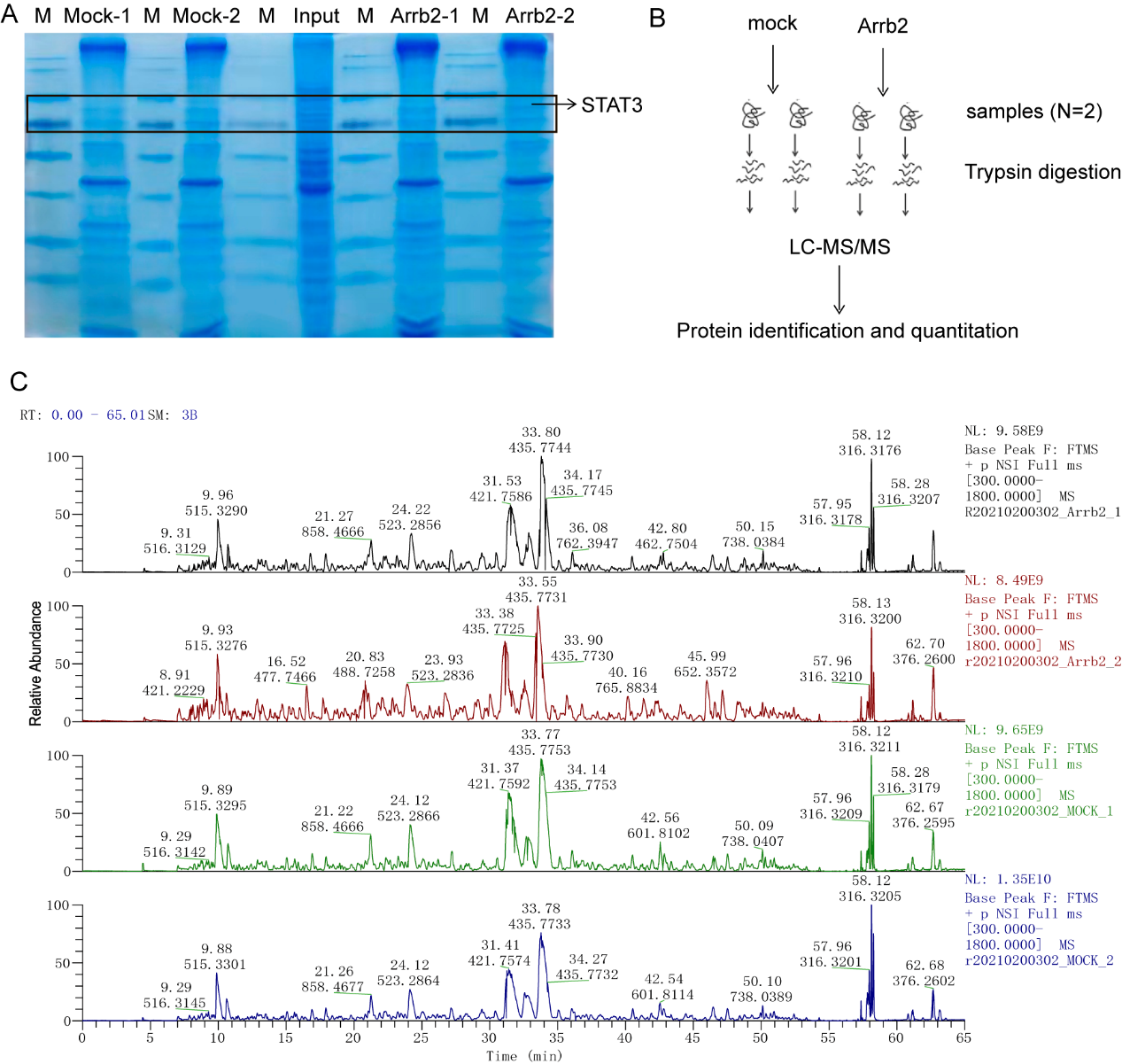


**Fig. S6.** **Mass spectrometry quantification of proteins that bind to β-arrestin2.** (A) Coomassie dye after SDS-PAGE. (B) Schematic summarizing of mass spectrometry quantification. (C) The base peak diagram of the tested samples (Mock-1, Mock-2, Arrb2-1, Arrb2-2).


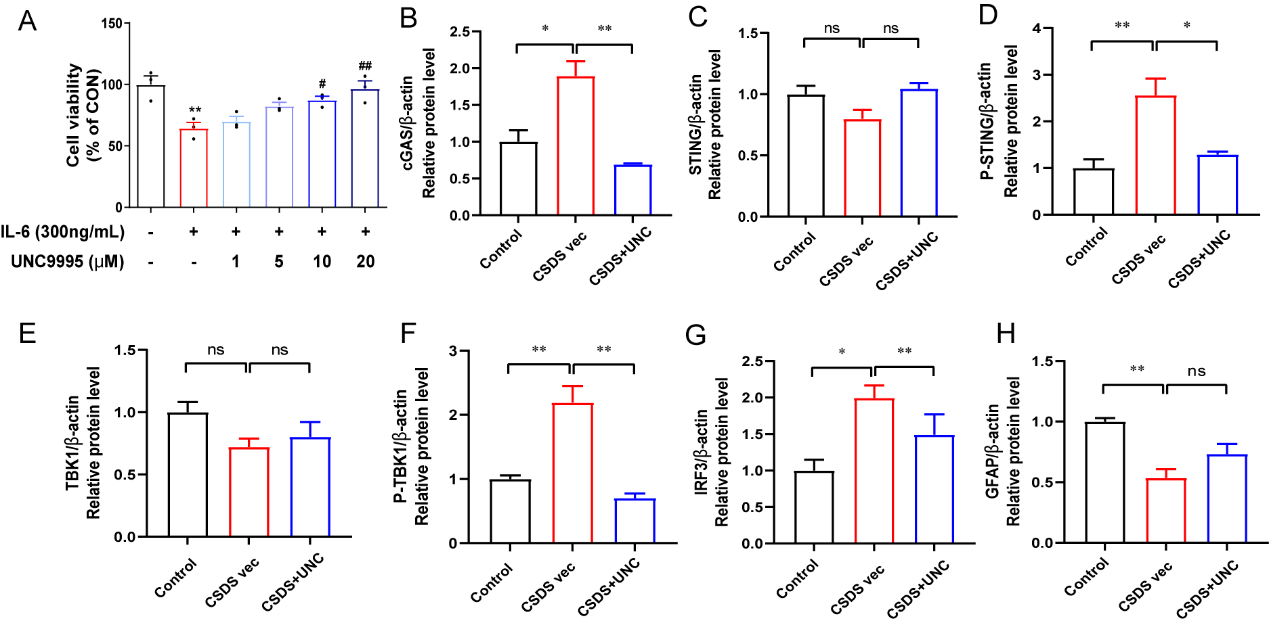


**Fig. S7.** **Immunoblot quantification of protein level in the hippocampus of Control, CSDS and CSDS mice treatment with UNC9995.** (A) Cell viability of astrocytes pretreated with UNC9995 (1, 5, 10, 20 μM) for 1h and then stimulated with IL-6 (300 ng/mL) for 24 h in astrocytes. n=3. Immunoblot quantification analysis of cGAS (B), STING (C), p-STING (D), TBK1(E), p-TBK1(F), IFN-*β* (G) and GFAP (H) in the hippocampus of control, CSDS vehicle, and CSDS mice treatment with UNC9995. Data were presented as mean ± SEM; statistically significant by Student t-test; (A) ***P* < 0.01 VS control, ^#^*P* <0.01, ^###^*P* < 0.001 VS IL-6 group. (B-H) Data are analyzed using one-way ANOVA, then combined with Dunnett's to assess the differences between groups. ns, *P*>0.05, **P*<0.05, ***P*<0.01, ****P*<0.001 VS Control group; ns, *P*>0.05, **P*<0.05, ***P*<0.01, ****P*<0.001 VS CSDS Susceptible group.


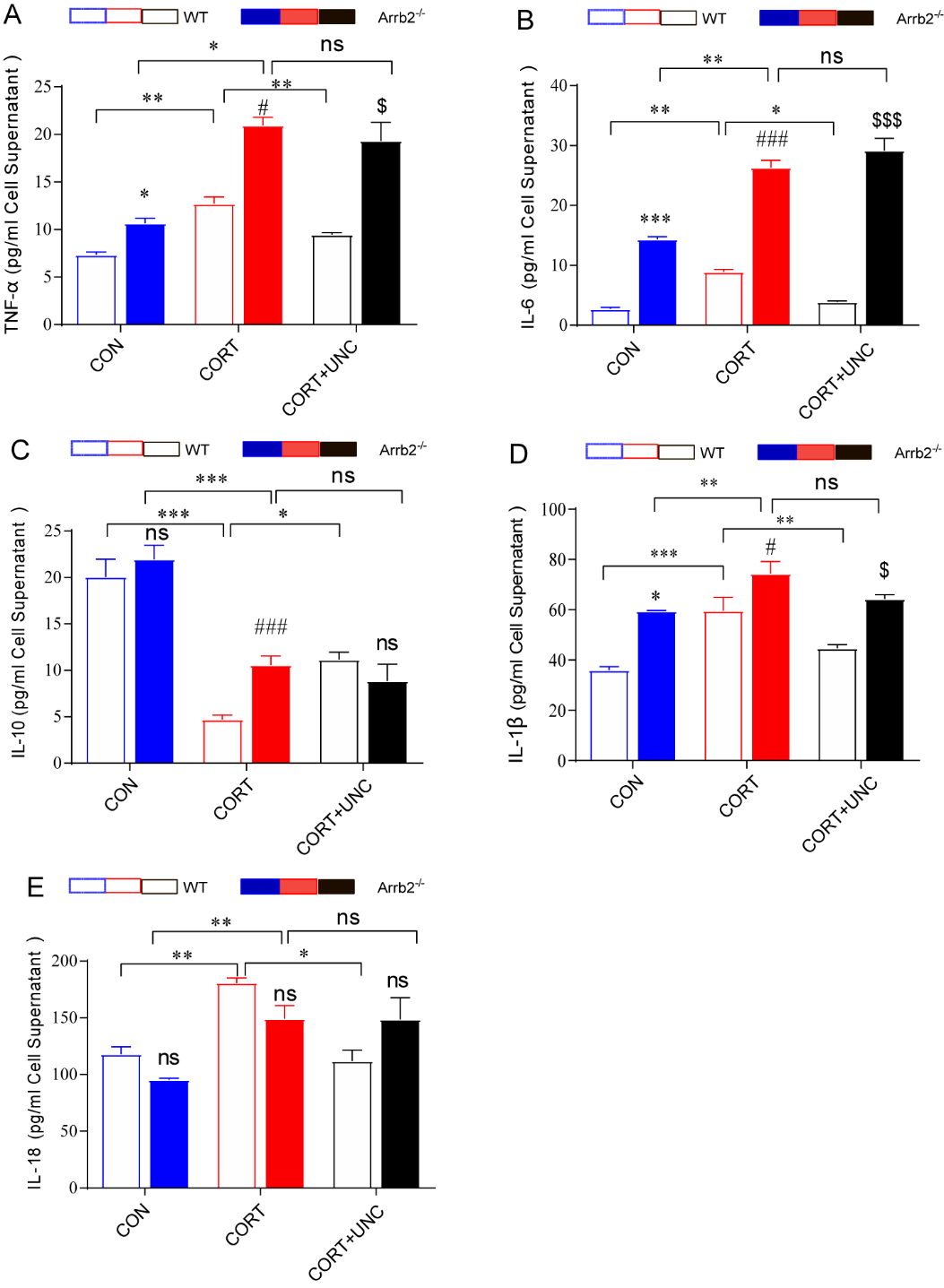


**Fig. S8. UNC9995 abolished the corticosterone-induced astrocytes inflammation which can be exacerbated by *β*-arrestin2 knockout.** (A-E) The Cytokines of TNF-*α*, IL-6, IL-10, IL-1*β,* and IL-18 were detected in the supernatant from primary WT and *Arrb2^-/-^* astrocytes induced by corticosterone pretreatment with UNC9995 (10 μM). n=6. Data are analyzed using two-way ANOVA, then combined with Tukey to assess the differences between groups. ns, *P* >0.05, **P* < 0.05, ***P* < 0.01, ****P* < 0.001 VS WT Control group. ns, *P* >0.05, ^#^*P* < 0.05, ^###^*P* < 0.001 VS WT CORT group. ns, *P* >0.05, ^$^*P* < 0.05, ^$$$^*P* < 0.001 VS WT CORT+UNC9995 group.

**Supplementary Table 1 Primers used in the study.**

| Primer name | Sequence |
| --- | --- |
| *m-Gapdh* | Forward: AACGACCCCTTCATTGAC |
|  | Reverse: TCCACGACATACTCAGCAC |
| *m-Tnf-α* | Forward: CCCTCCTTCAGACACCCT |
|  | Reverse: GGTTGCCAGCACTTCACT |
| *m-Il-1β* | Forward: TTGAGTCTGCCCAGTTCC |
|  | Reverse: TTTCTGCTTGAGAGGTGCT |
| *m-Il-6* | Forward: CAATAACCACCCCTGACC |
|  | Reverse: GCGCAGAATGAGATGAGTT |
| *m-Il-18* | Forward: GACAGCCTGTGTTCGAGGATATG |
|  | Reverse: TGTTCTTACAGGAGAGGGTAGAC |
| *m-cGAS* | Forward: TGAAACAGGTGCTTTCTATCTTGTG |
|  | Reverse: GTGTTACAGCAGGGCTTCCT |
| *m-Sting* | Forward: AGGCGTCTGTATCCTGGAGT |
|  | Reverse: CAAGTGTCCGGCAGAAGAGT |
| *m-IFN-β* | Forward: CCCTATGGAGATGACGGAGA |
|  | Reverse: CCCAGTGCTGGAGAAATTGT |
| *m-IRF-3* | Forward: CTACGGCAGGACGCACAGAT |
|  | Reverse: TCAGCAGCTAACCGCAACAC |
| *m-IRF-7* | Forward: CAGCACAGGGCGTTTTATCTT |
|  | Reverse: TCTTCCCTATTTTCCGTGGC |
| *m-Drd1* | Forward: AGATGACTCCGAAGGCAGCCTT |
|  | Reverse: GCCATGTAGGTTTTGCCTTGTGC |
| *m-Drd2* | Forward: CCTGTCCTTCACCATCTCTTGC |
|  | Reverse: TAGACCAGCAGGGTGACGATGA |
| *m-Drd3* | Forward: GCCAGTGTATCAGCATCAGACC |
|  | Reverse: GTGGCATCTGACAGAAACCTGG |
| *m-Drd4* | Forward: CTGCCTCTCTTTGTCTACTCCG |
|  | Reverse: ACAGGTTGAAGATGGAGGCGGT |
| *m-Drd5* | Forward: TTGCTCGGCAACGTCCTAGTGT |
|  | Reverse: ACCAGCAATGCCACGAAGAGGT |
| *m-β-arr2* siRNA | Forward: GCUUGUGGAGUAGACUUUGTT |
|  | Reverse: CAAAGUCUACUCCACAAGCTT |
| *m-β-arr2* negative control | Forward: UUCUCCGAACGUGUCACGUTT |
|  | Reverse: ACGUGACACGUUCGGAGAATT |
