# Supplementary material for: β-Arrestin2-biased Drd2 agonist UNC9995 alleviates astrocyte inflammatory injury via interaction between β-arrestin2 and STAT3 in mouse model of depression
Source: J Neuroinflammation. 2022 Oct 1;19:240. doi: 10.1186/s12974-022-02597-6 (PMC9526944; doi:10.1186/s12974-022-02597-6)

Fig.2C

csds-cGAS/actin

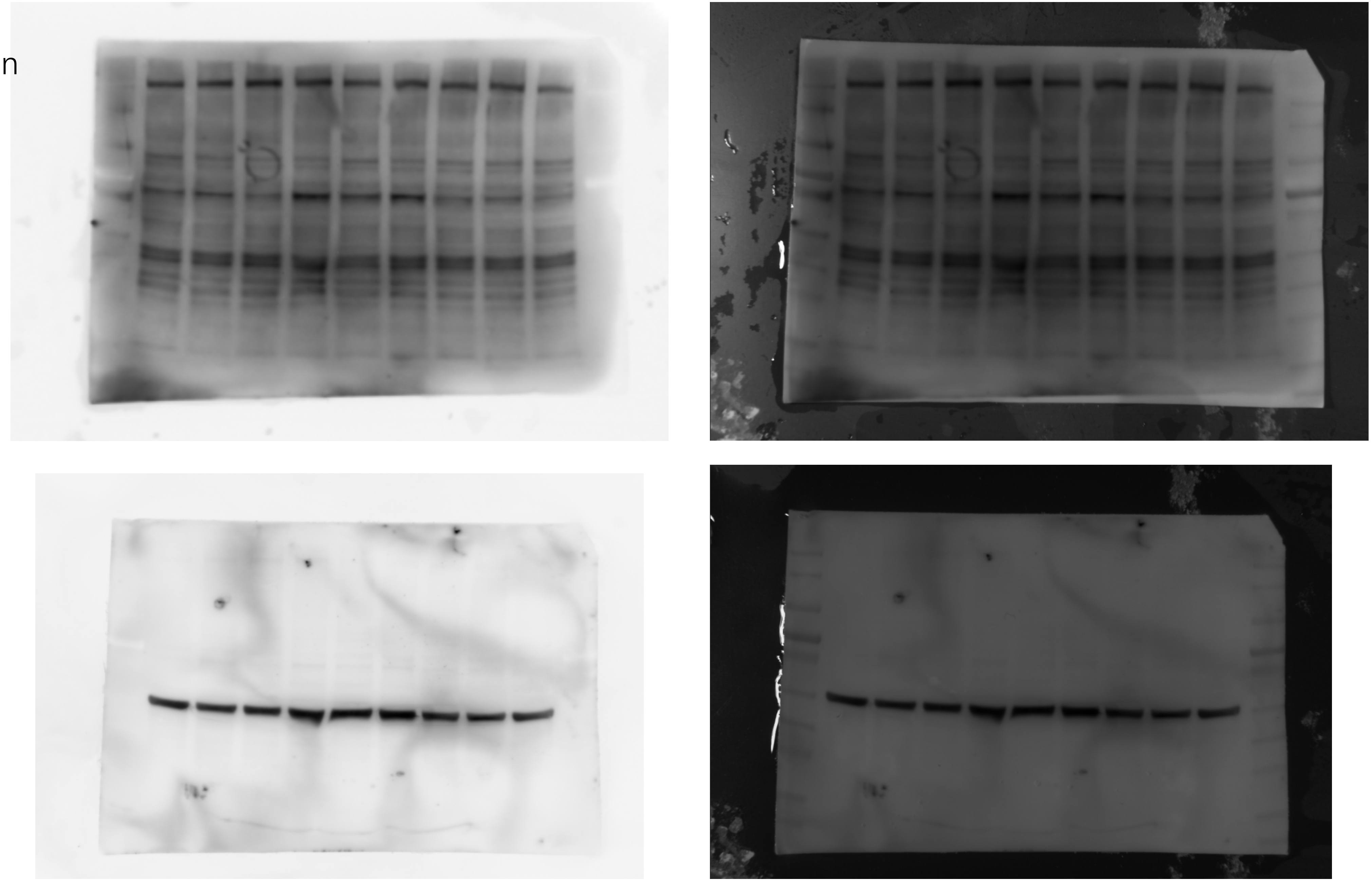

Fig.2C

CSDS-p-TBK1

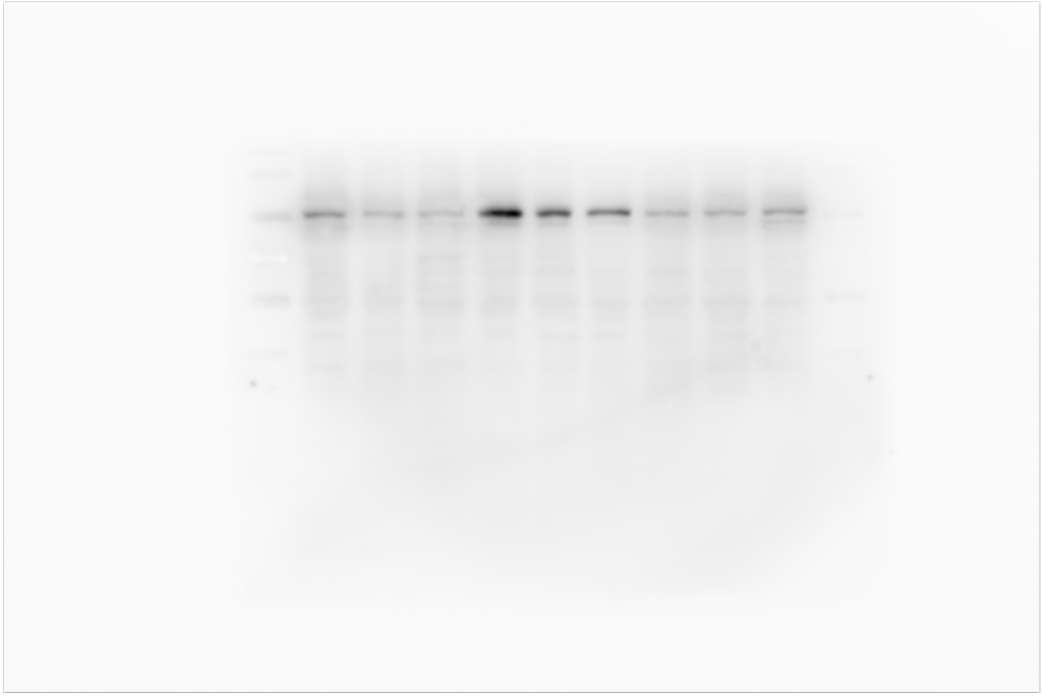

Fig.2C

csds-TBK1

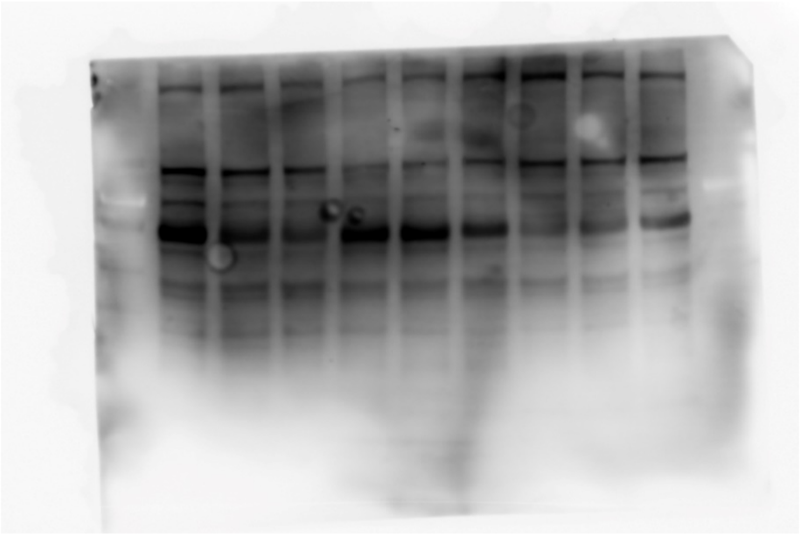

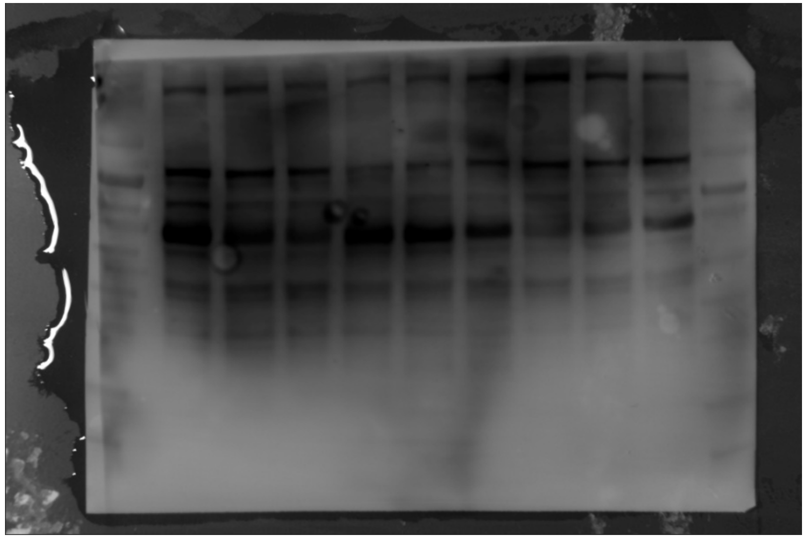

Fig.2C

csds-p-sting/actin

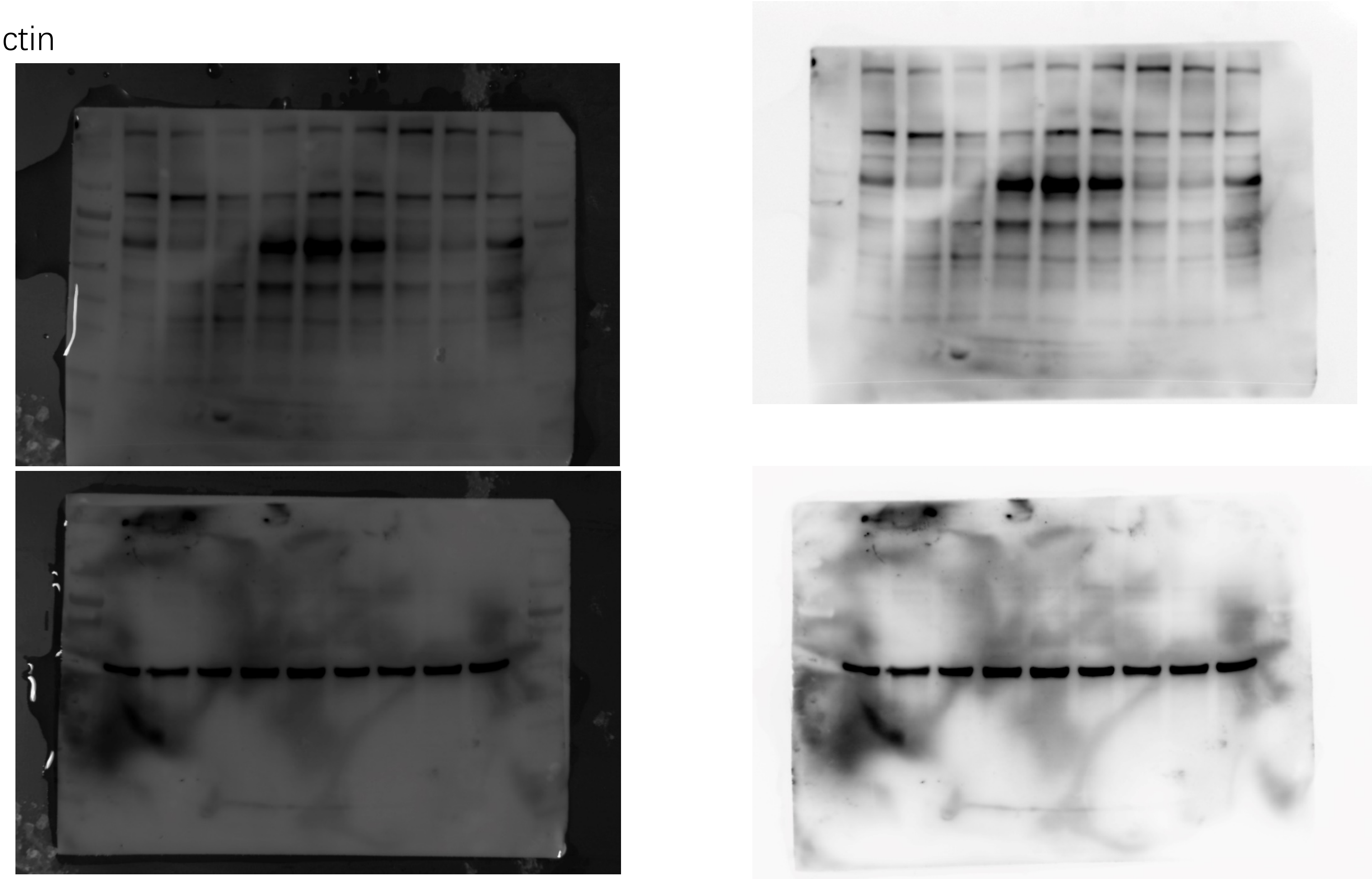

Fig.2C
csds-STING

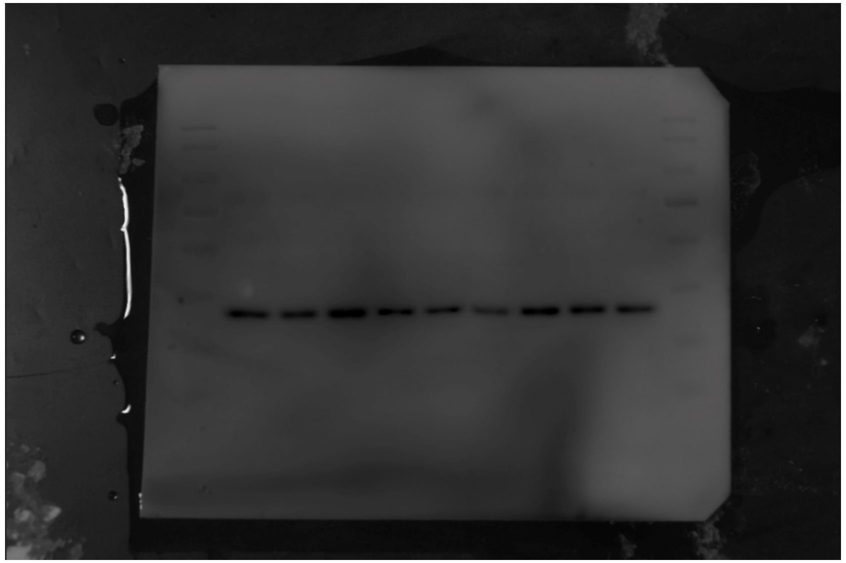

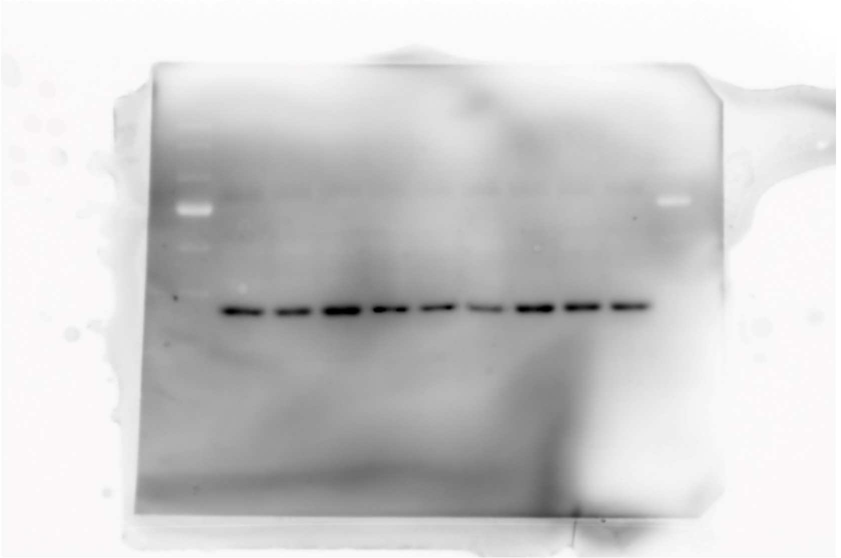

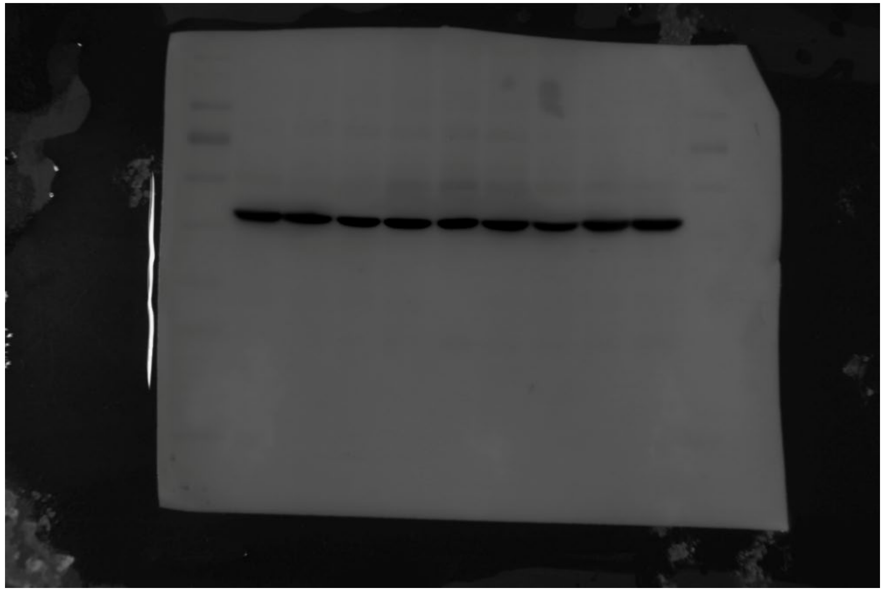

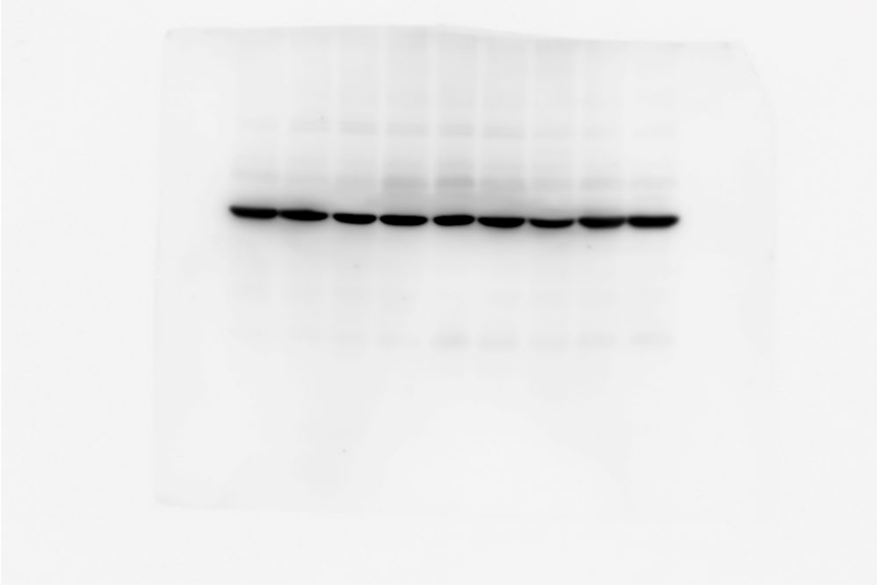

Fig.2C

csds-irf3/
gapdh

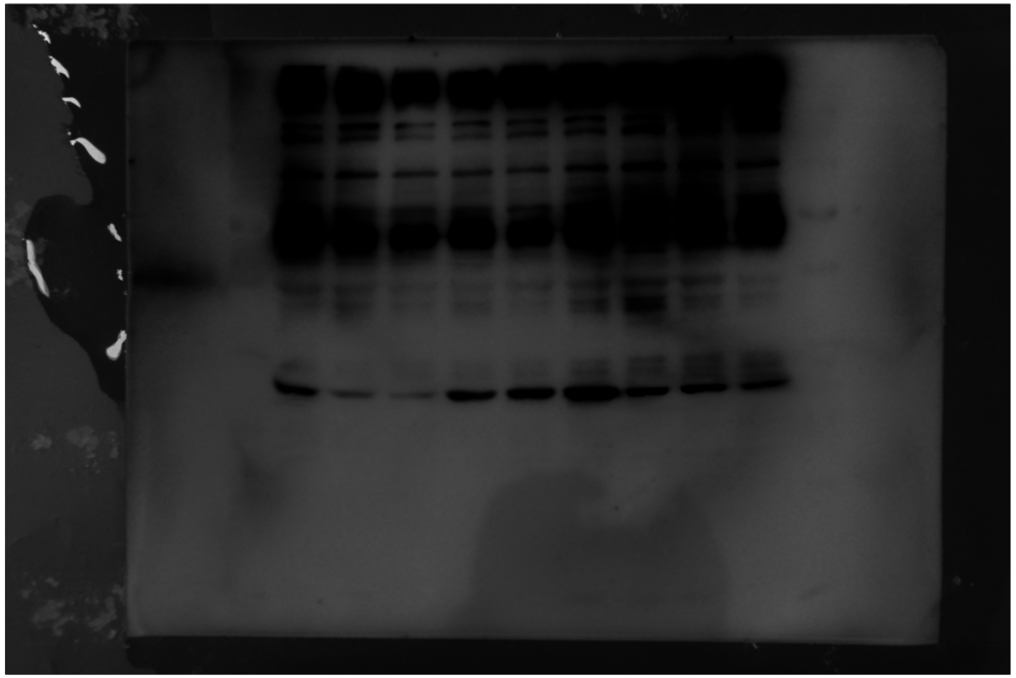

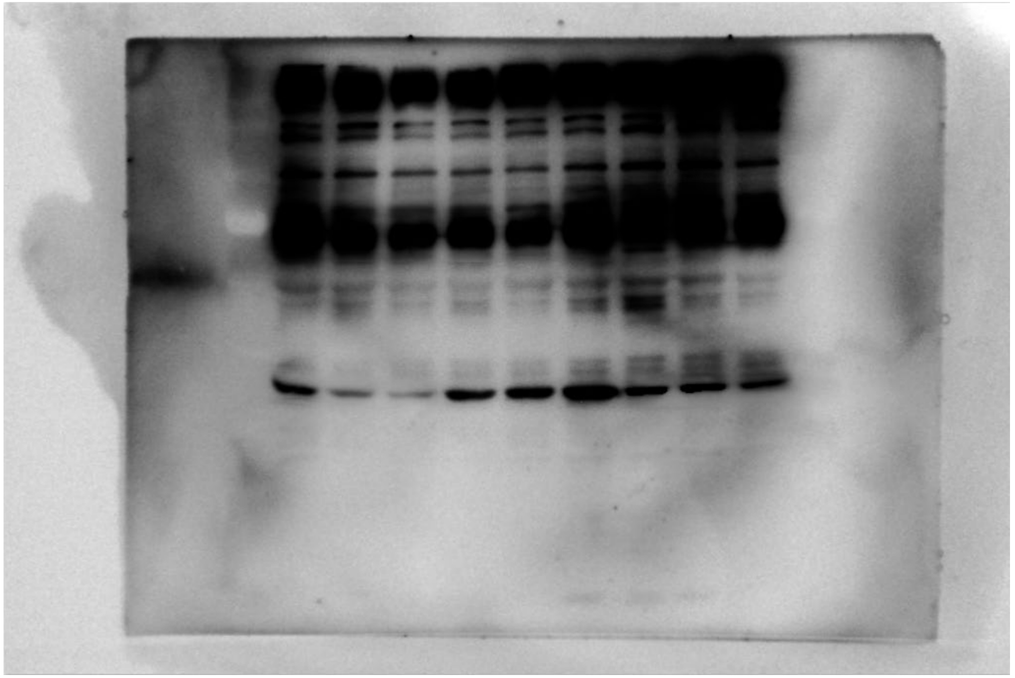

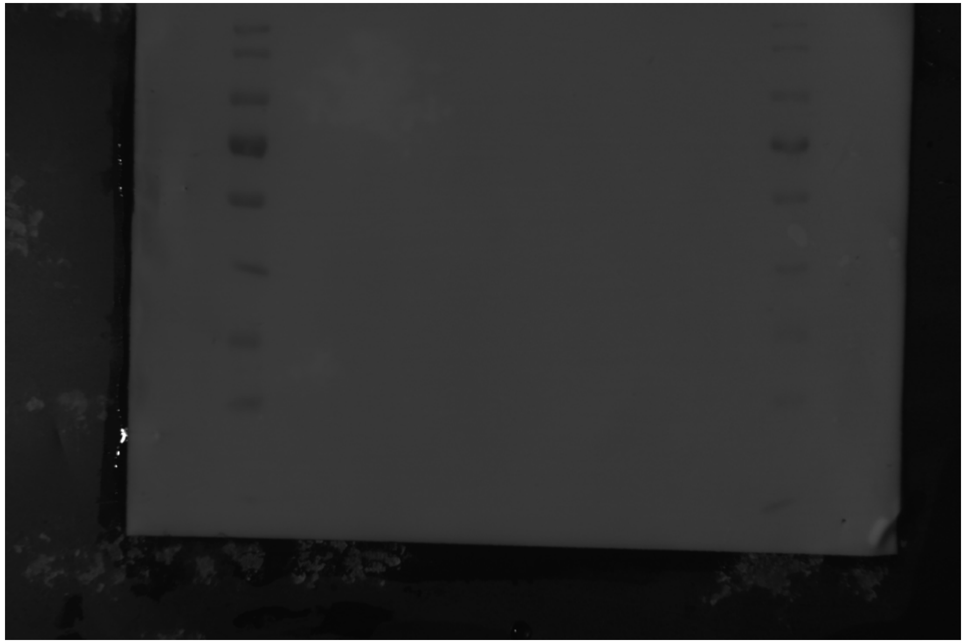

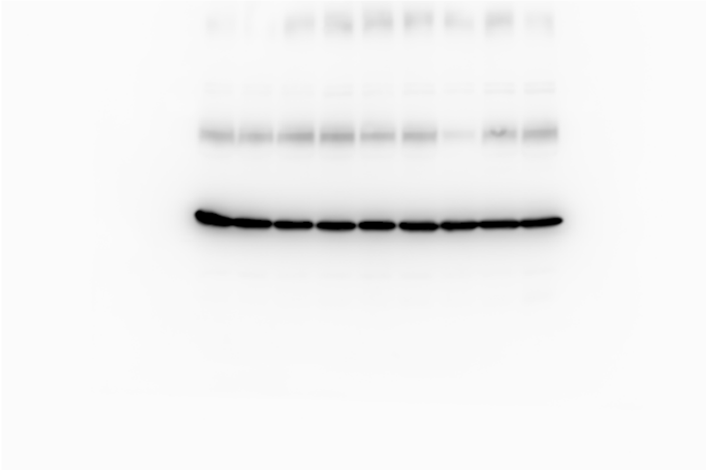

Fig.3B

Csds-DRD1

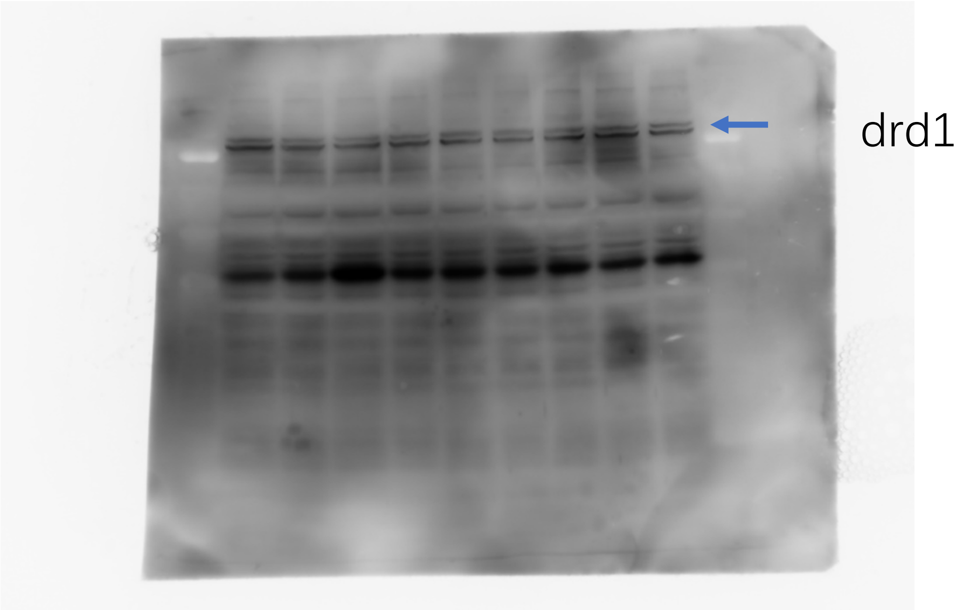

actin

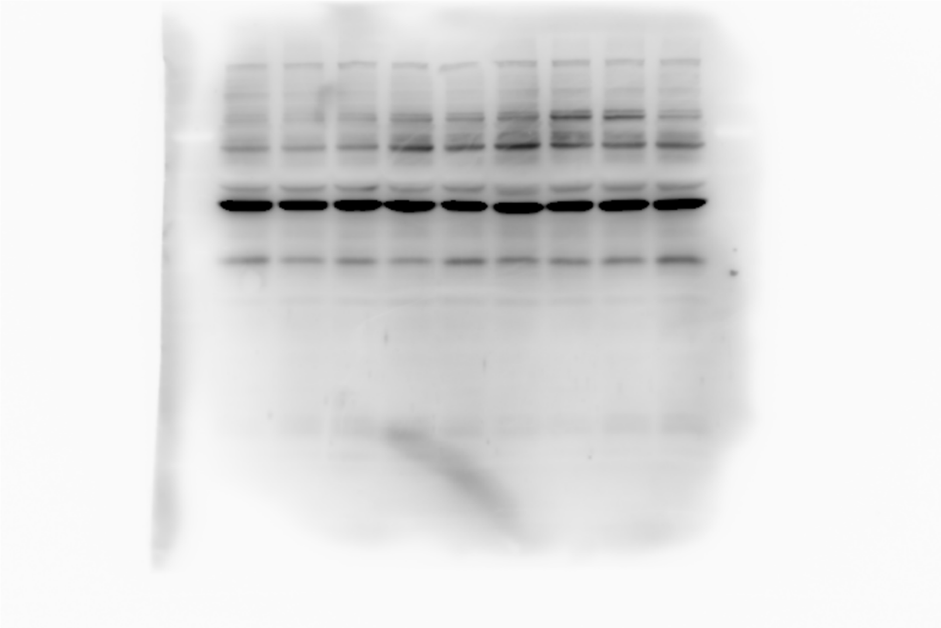

Fig.3B

Csds-DRD2

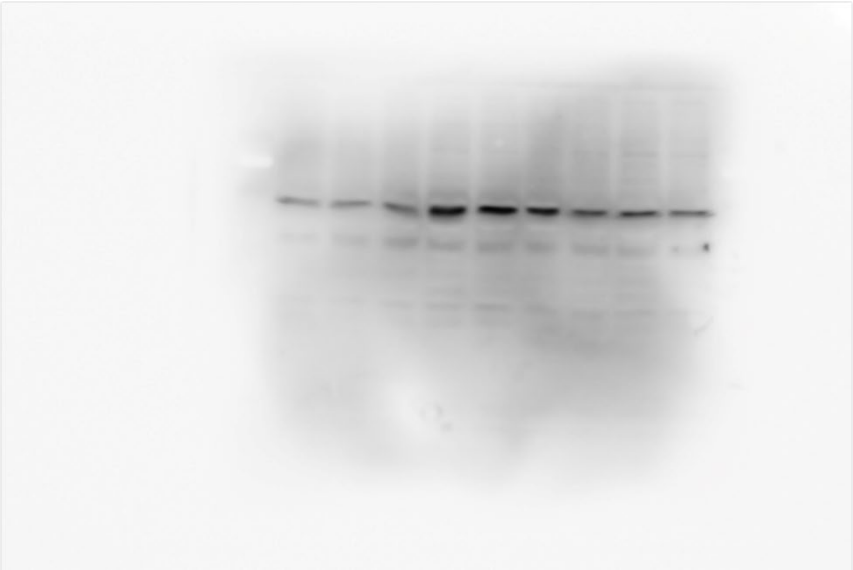

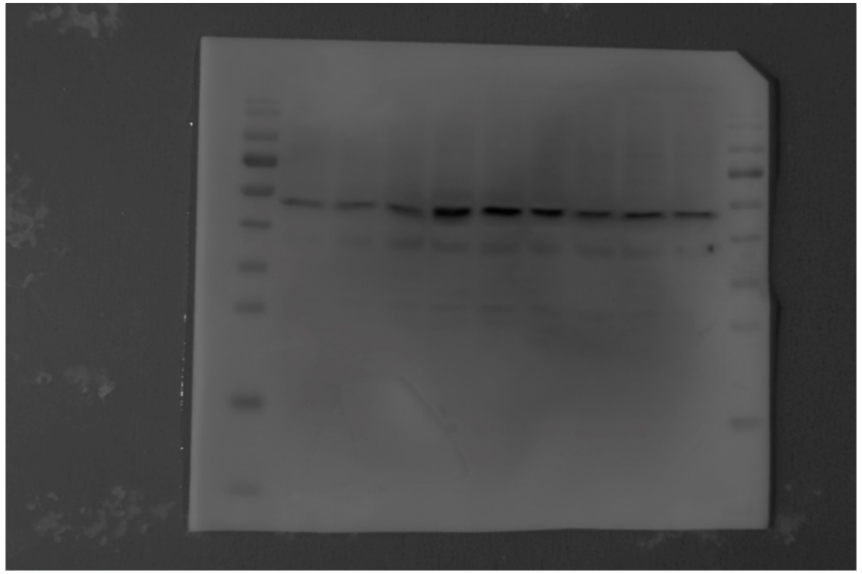

actin

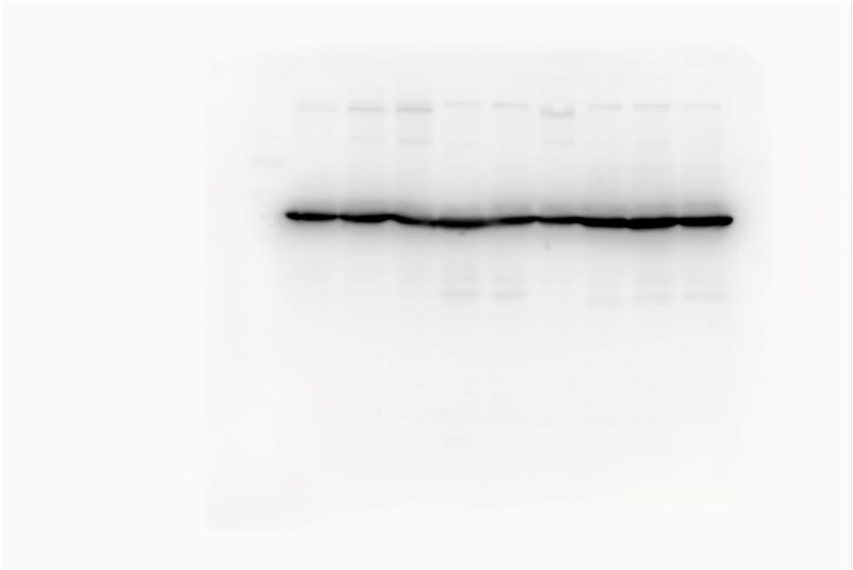

Fig.3B

CSDS-ARRB1

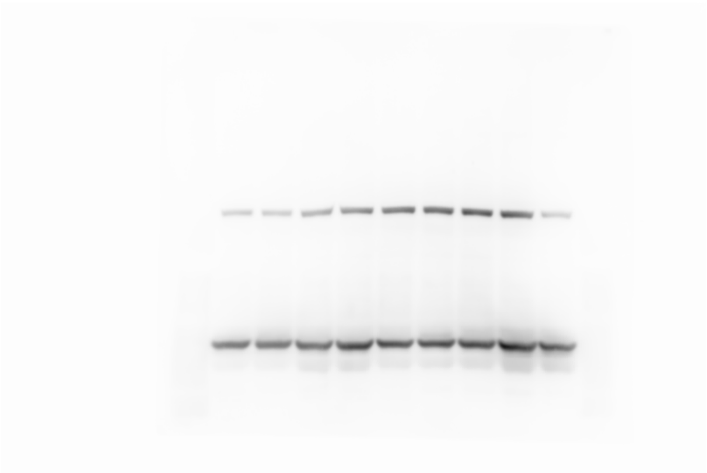

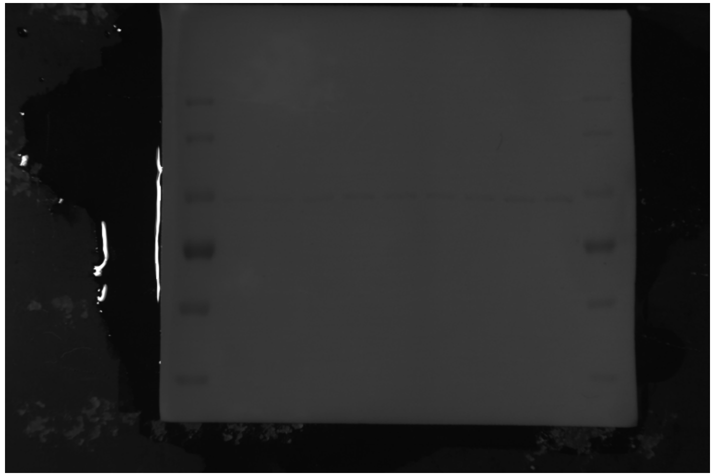

Actin

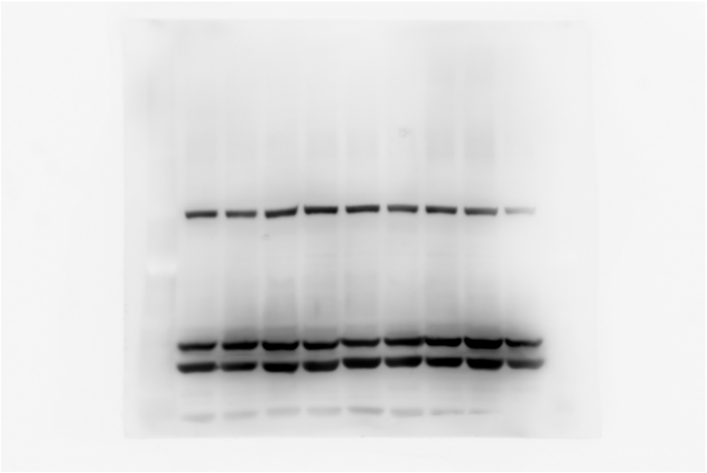

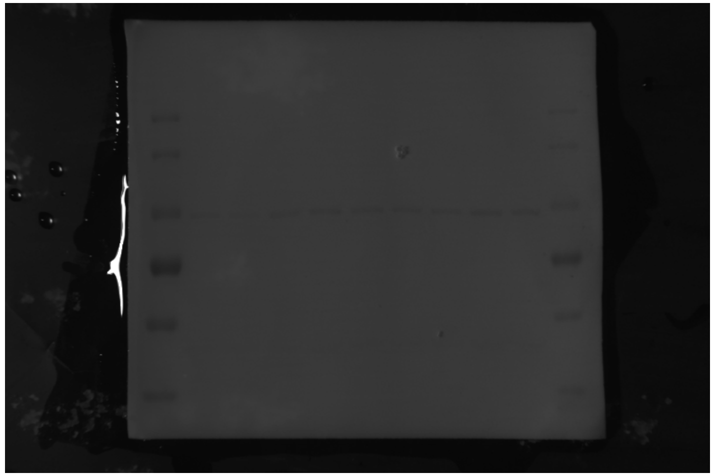

Fig.3B

CSDS-ARRB2

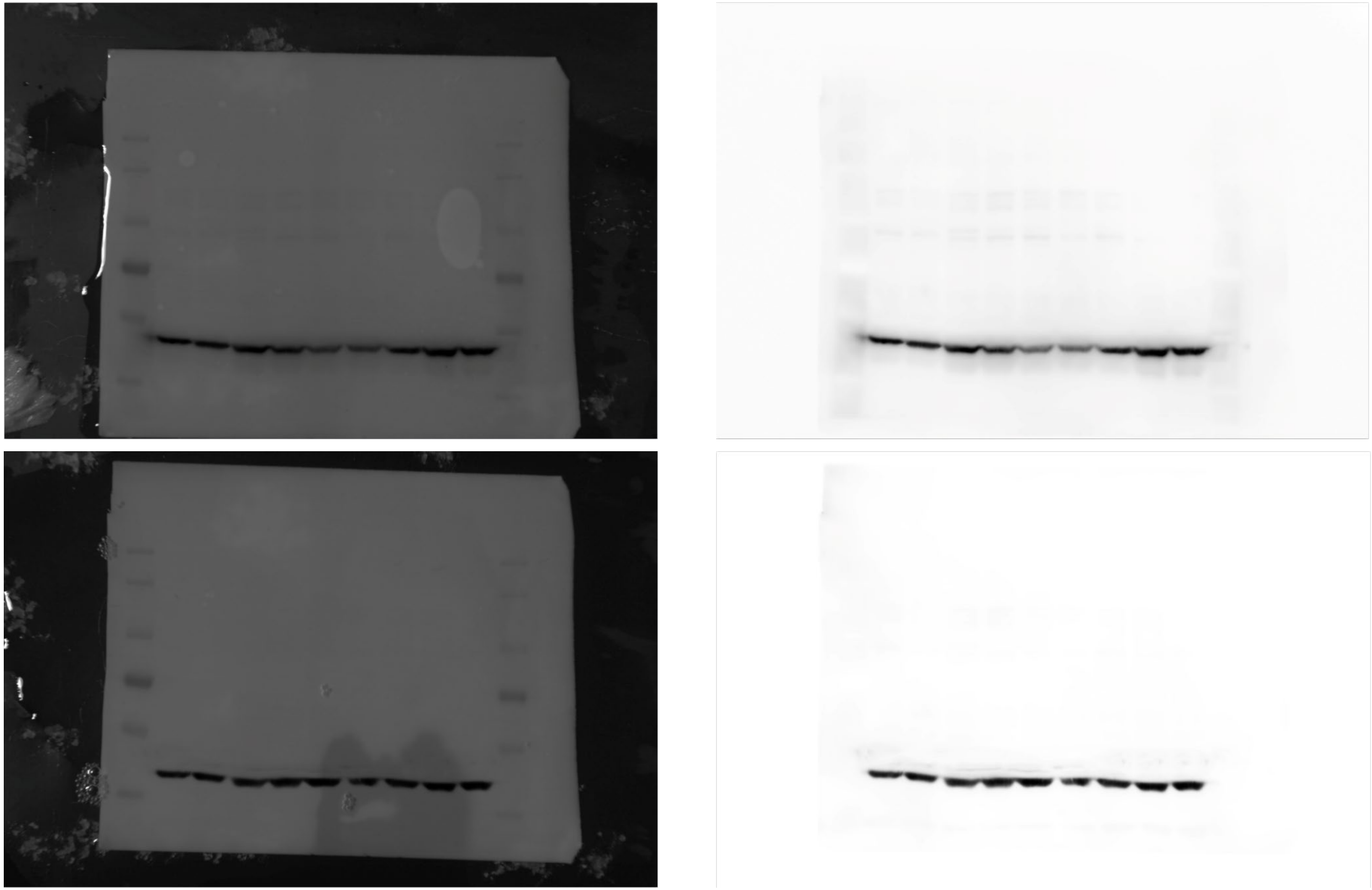

Fig.3B

CSDS-gfap

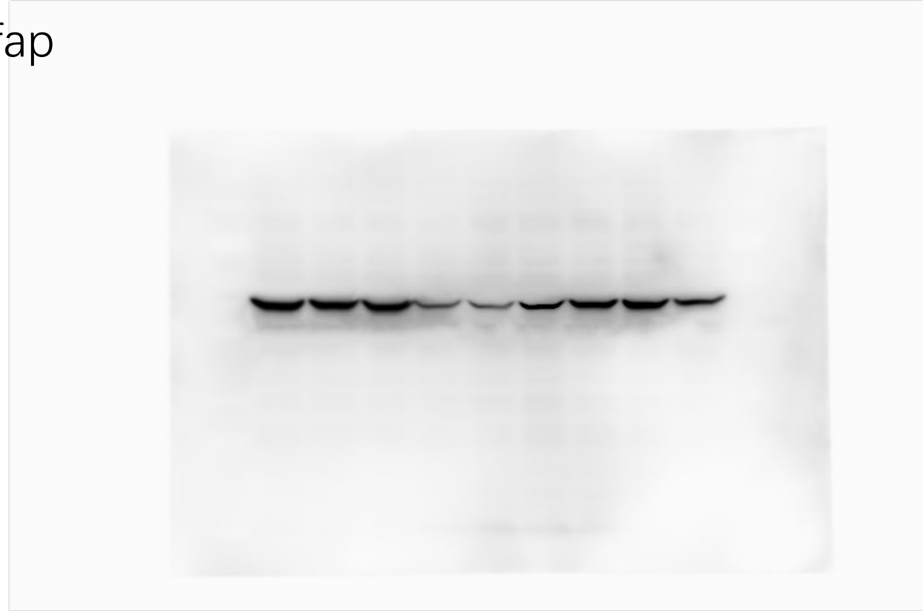

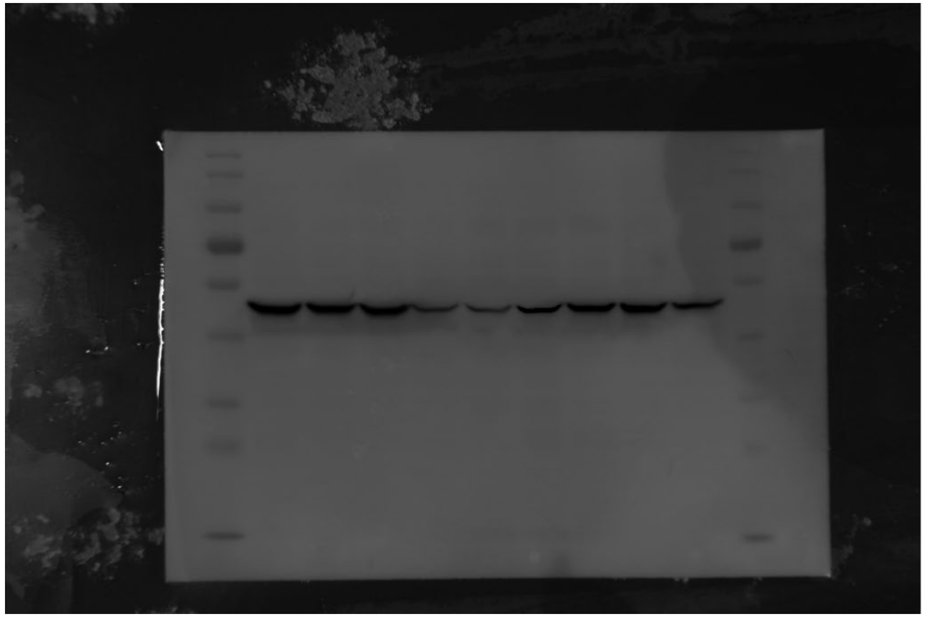

Actin（下面一条）

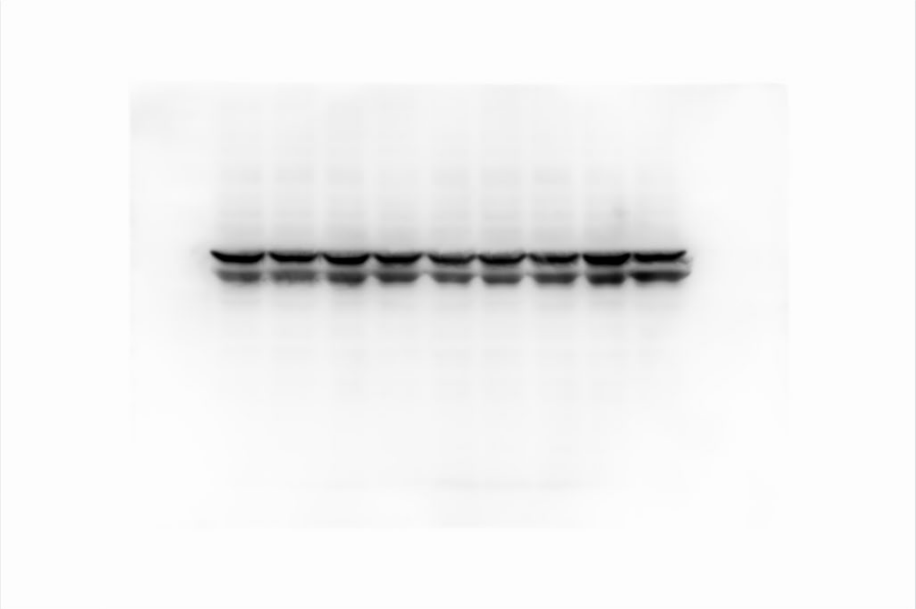

Fig.4F

WT&Arrb2$^{-/-}$-As
IL-6 stimulation

BAX

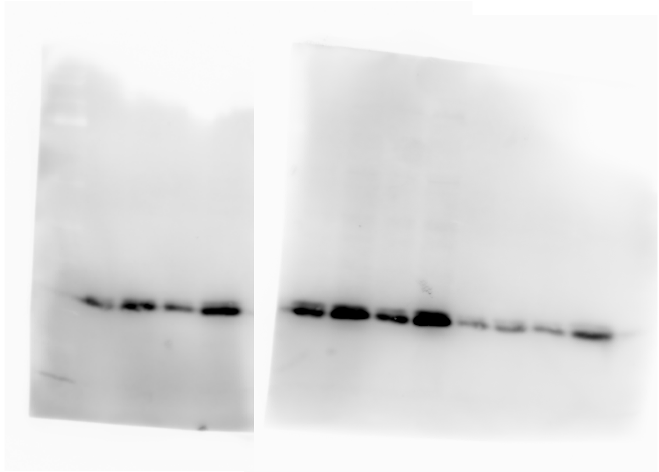

Actin

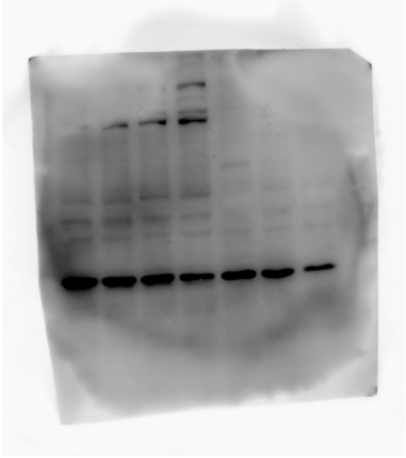

WT&Arrb2$^{-/-}$-As
IL-6 stimulation

Bcl-2

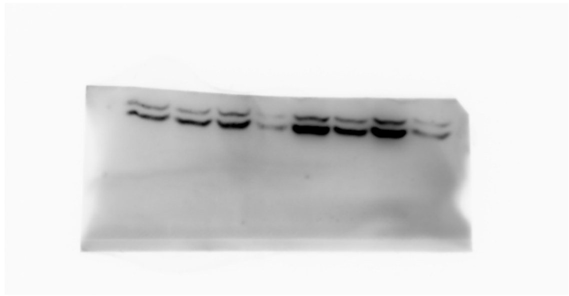

Actin

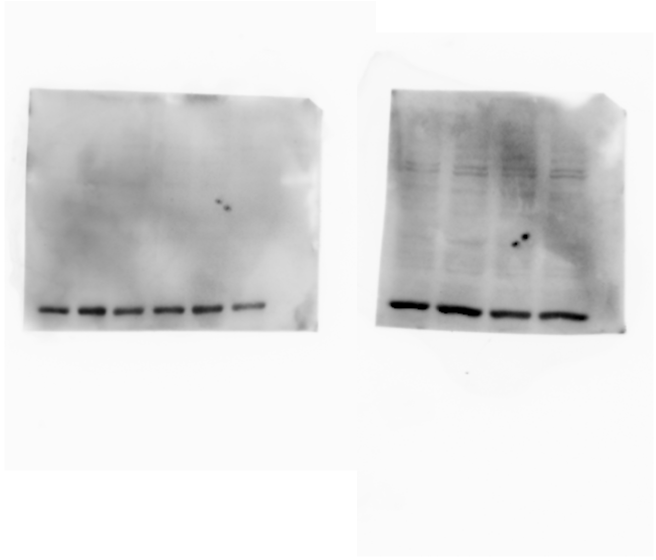

Fig.4I

WT&Arrb2$^{-/-}$-As
IL-6 stimulation-Nucleus

P-STAT3

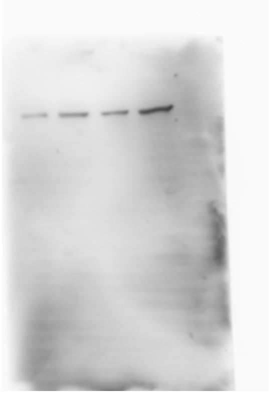

STAT3

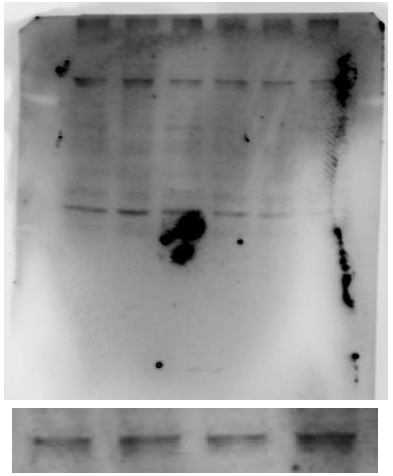

laminB1

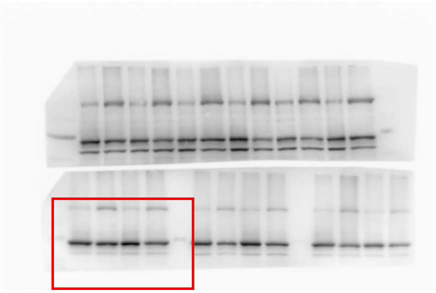

Fig.4G

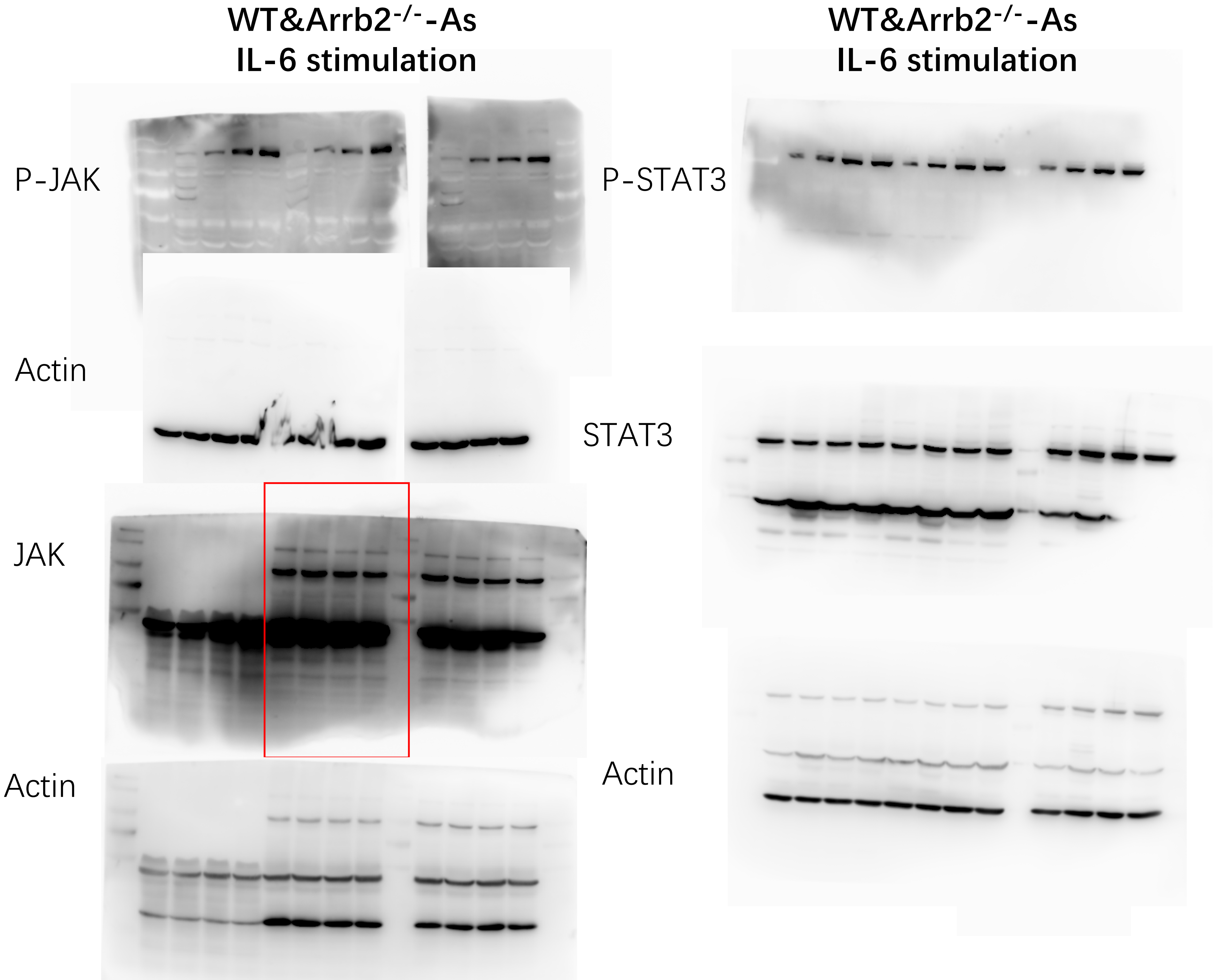
WT&Arrb2-/--As
IL-6 stimulation
P-JAK
Actin
JAK
Actin
WT&Arrb2-/--As
IL-6 stimulation
P-STAT3
STAT3
Actin

Fig.4H

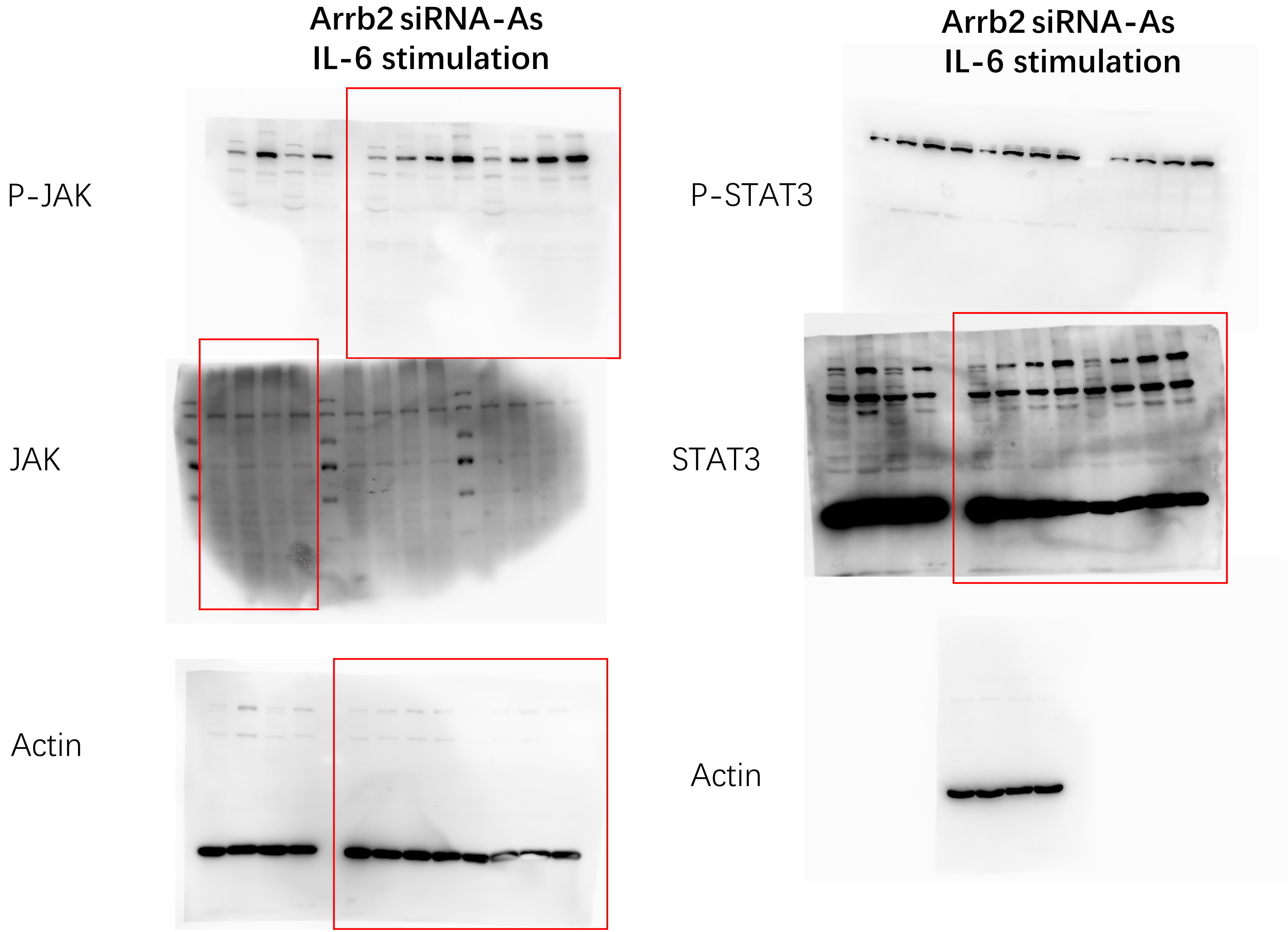
Arrb2 siRNA-As
IL-6 stimulation
P-JAK
JAK
Actin
Arrb2 siRNA-As
IL-6 stimulation
P-STAT3
STAT3
Actin

Fig.5F

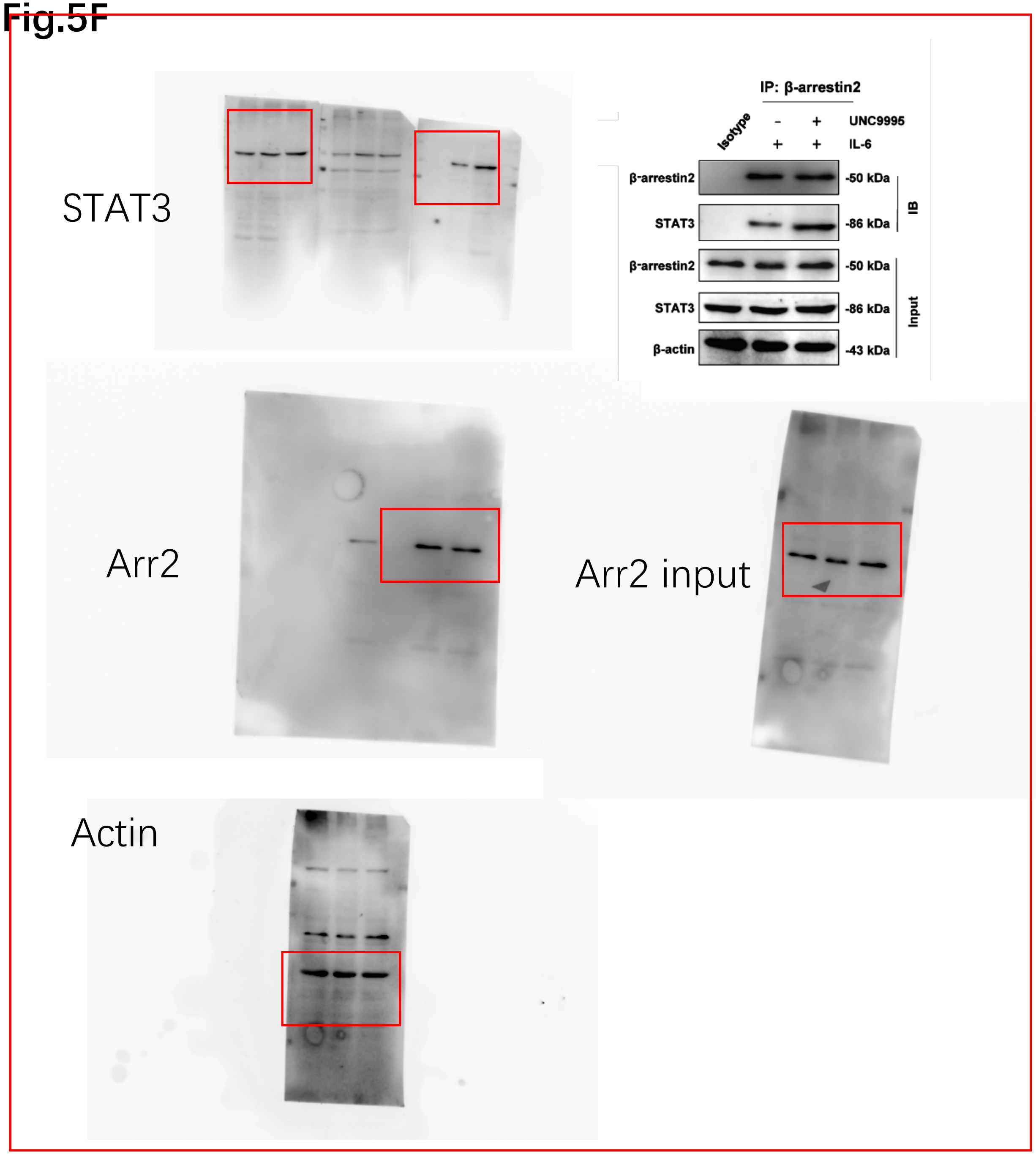
STAT3
Arr2
Arr2 input
Actin
IP: β-arrestin2
Isotype
- + UNC9995
+ + IL-6
β-arrestin2 -50 kDa
STAT3 -86 kDa
IB
β-arrestin2 -50 kDa
STAT3 -86 kDa
β-actin -43 kDa
Input

Fig.5D

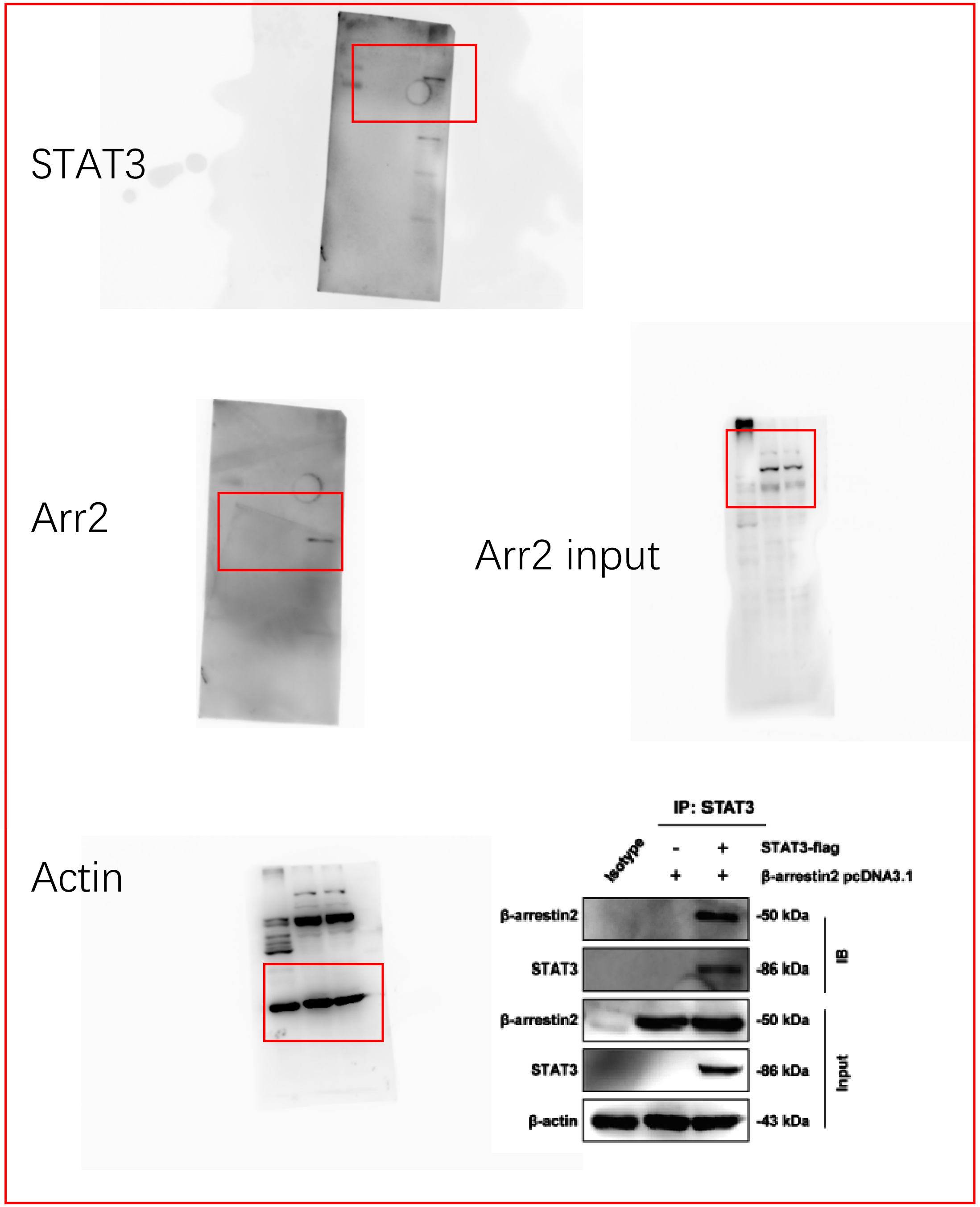
STAT3
Arr2
Arr2 input
Actin
IP: STAT3
Isotype
- + STAT3-flag
+ + β-arrestin2 pcDNA3.1
β-arrestin2 -50 kDa
STAT3 -86 kDa
IB
β-arrestin2 -50 kDa
STAT3 -86 kDa
β-actin -43 kDa
Input

Fig.5E

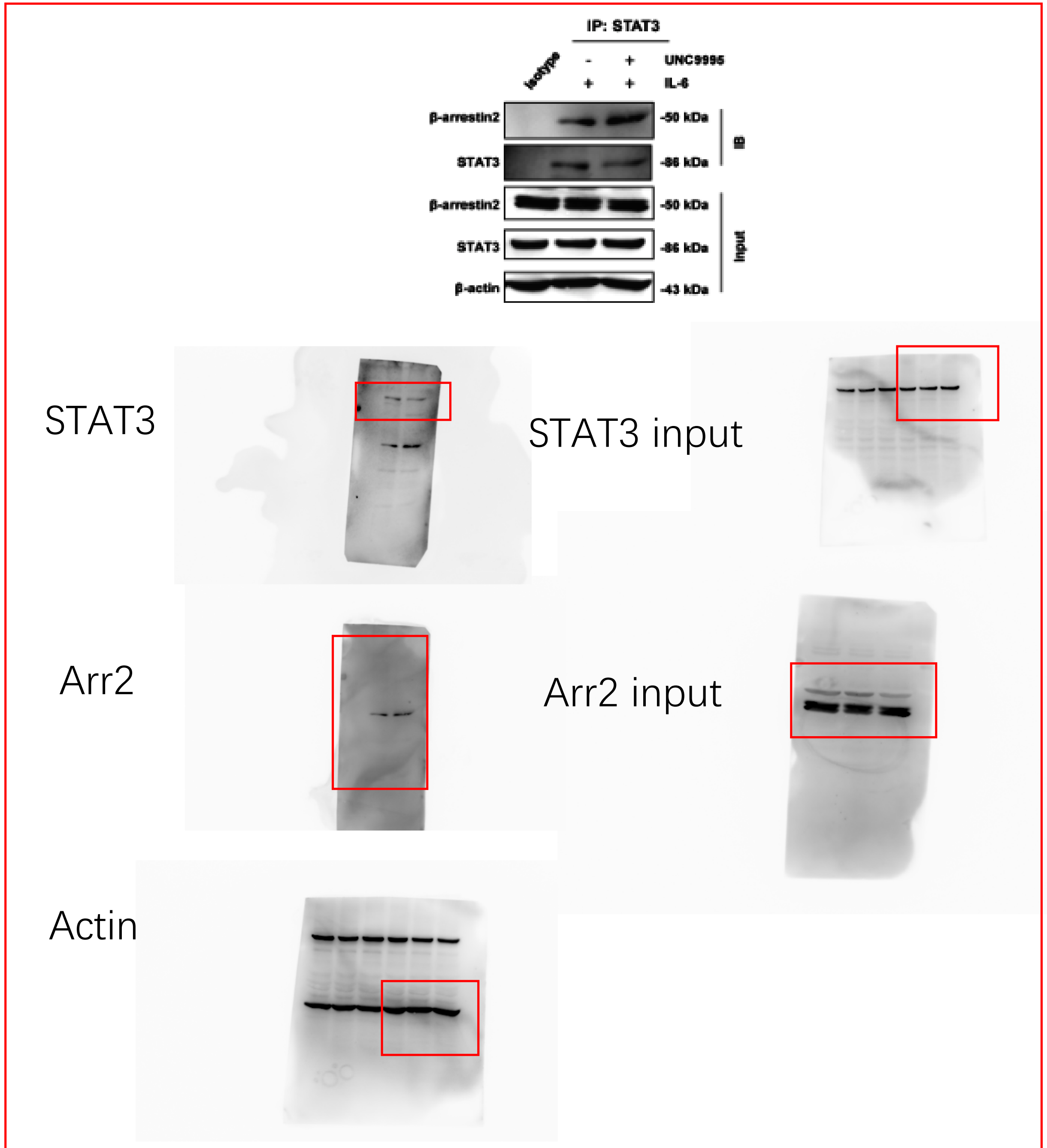
IP: STAT3
Isotype
- + UNC9995
+ + IL-6
β-arrestin2 -50 kDa
STAT3 -86 kDa
IB
β-arrestin2 -50 kDa
STAT3 -86 kDa
β-actin -43 kDa
Input
STAT3
STAT3 input
Arr2
Arr2 input
Actin

Fig.5G

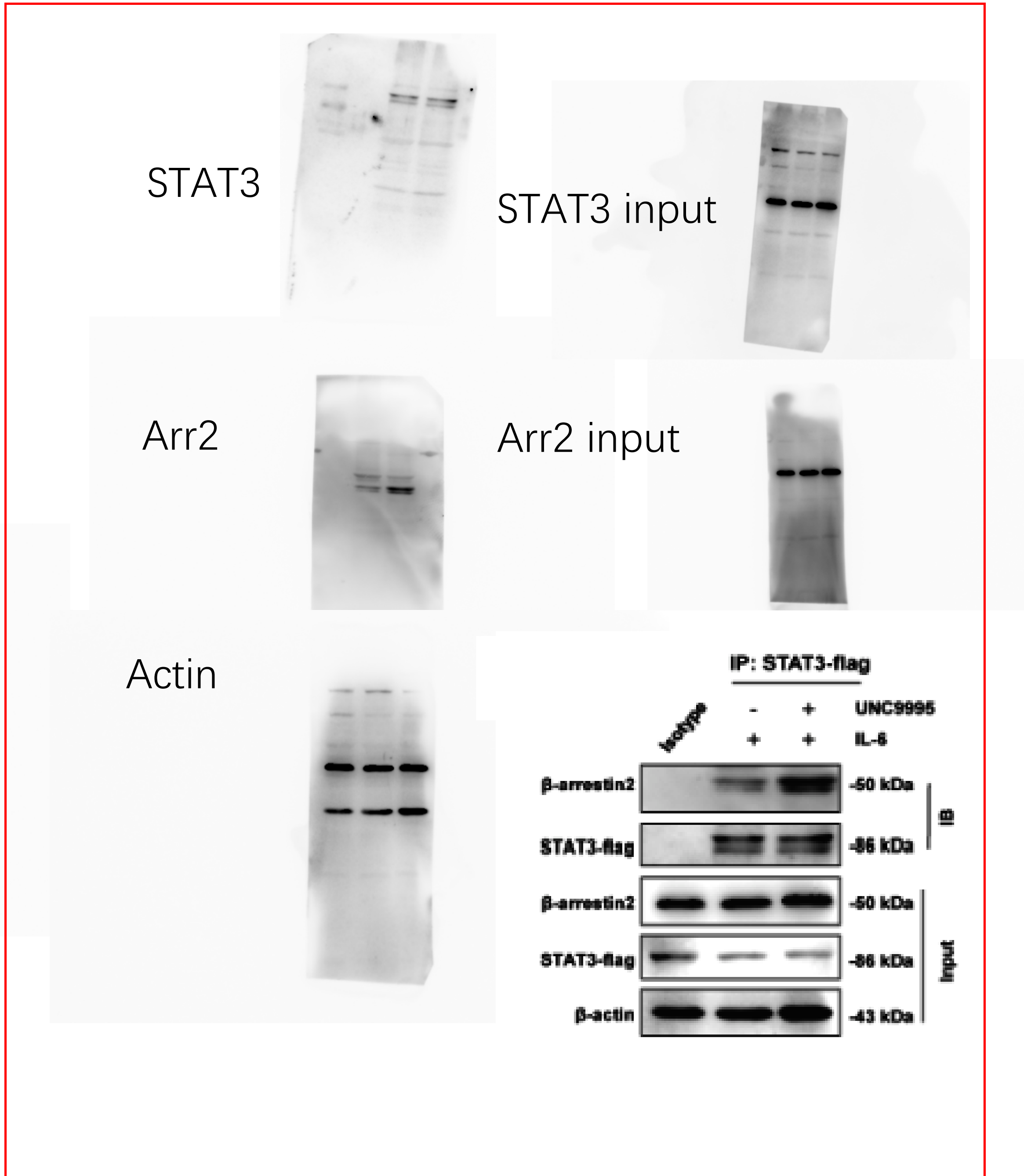
STAT3
STAT3 input
Arr2
Arr2 input
Actin
IP: STAT3-flag
Isotype
- + UNC9995
+ + IL-6
β-arrestin2 -50 kDa
STAT3-flag -86 kDa
IB
β-arrestin2 -50 kDa
STAT3-flag -86 kDa
β-actin -43 kDa
Input

Fig.6E

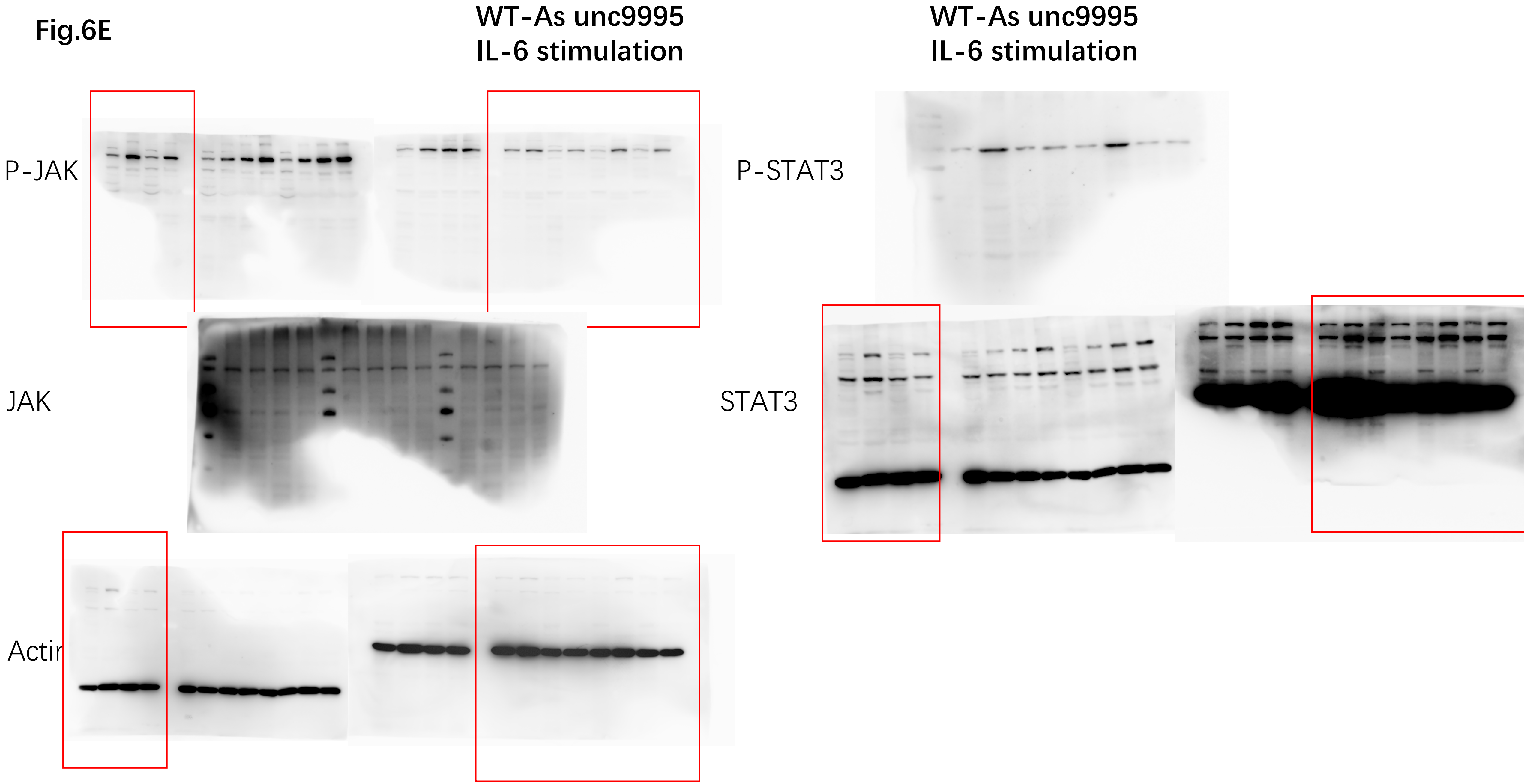

WT-As unc9995
IL-6 stimulation
WT-As unc9995
IL-6 stimulation
P-JAK
P-STAT3
JAK
STAT3
Actin

Fig.6E

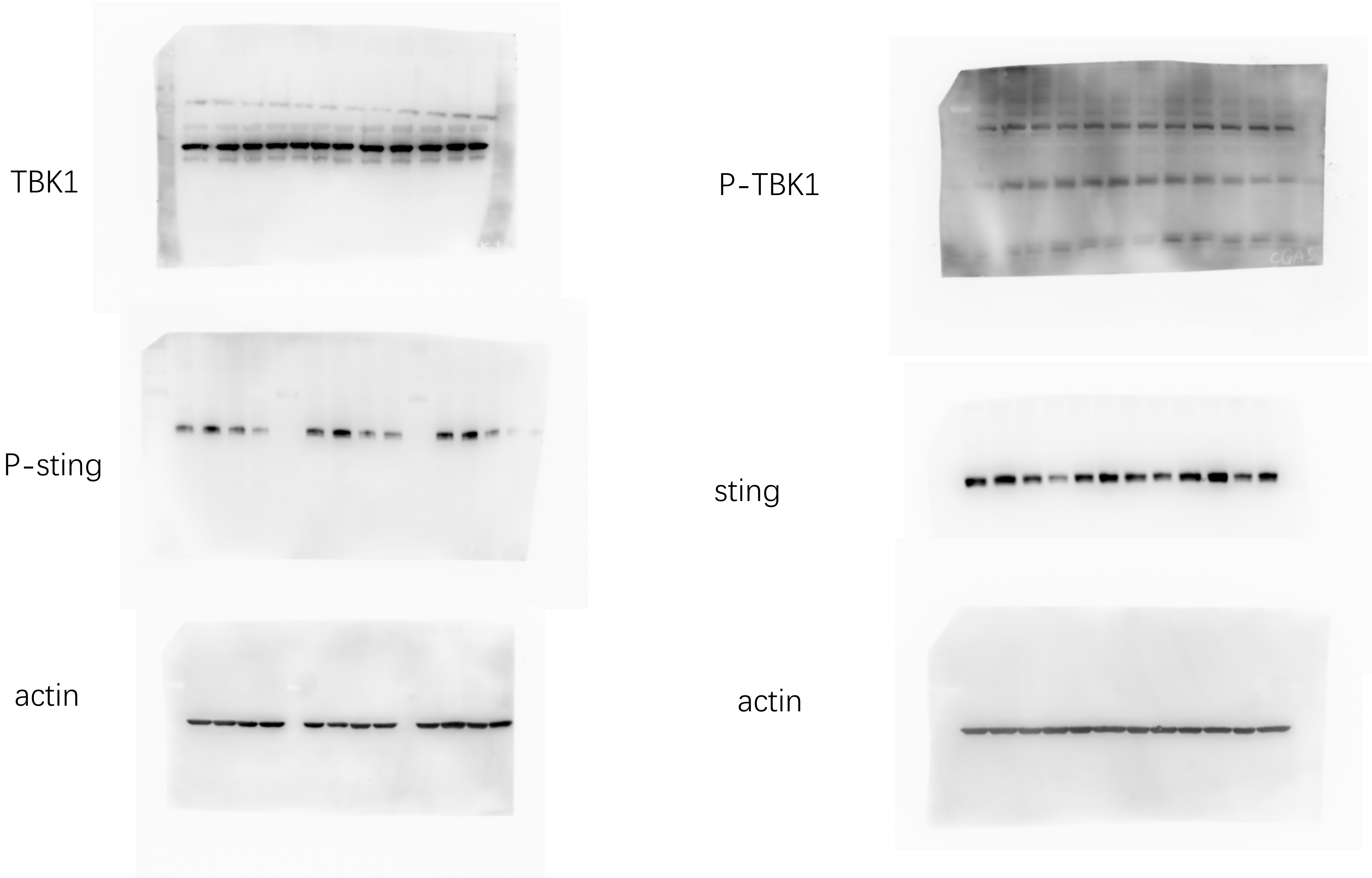

Fig.6F

WT-As unc9995
IL-6 stimulation

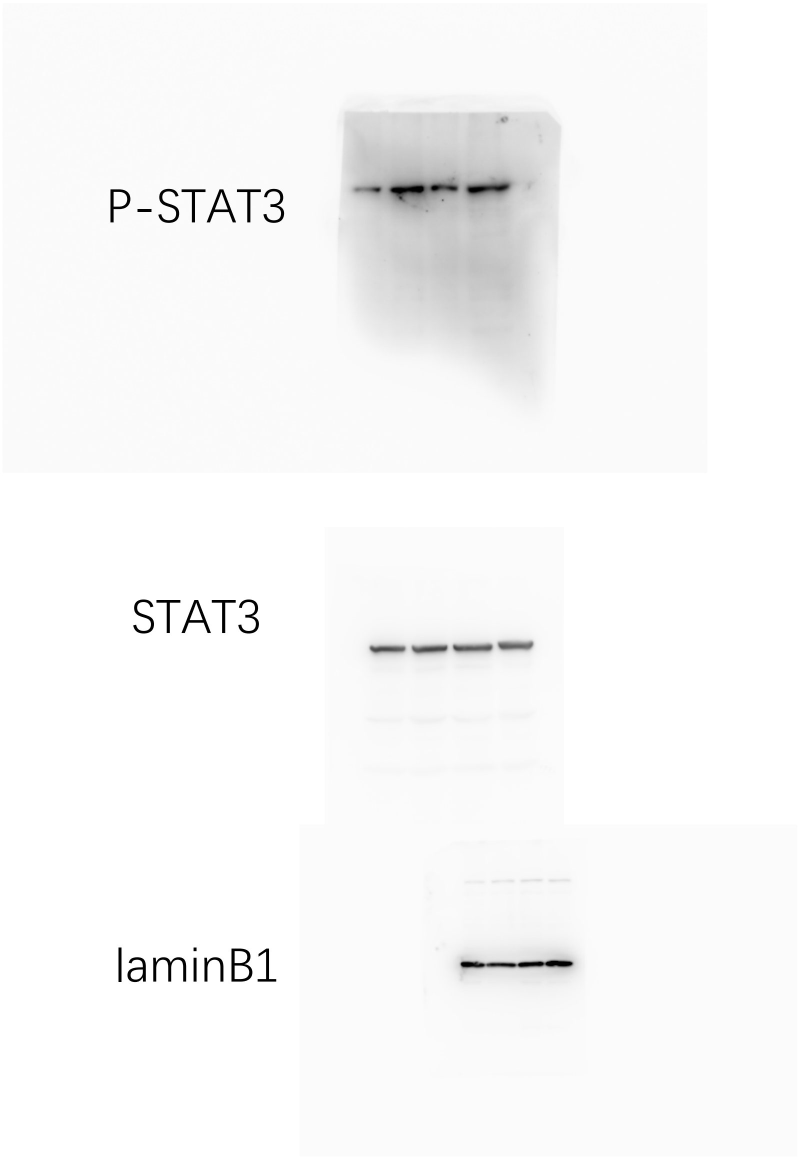

Fig.7G

Unc治疗-gfap

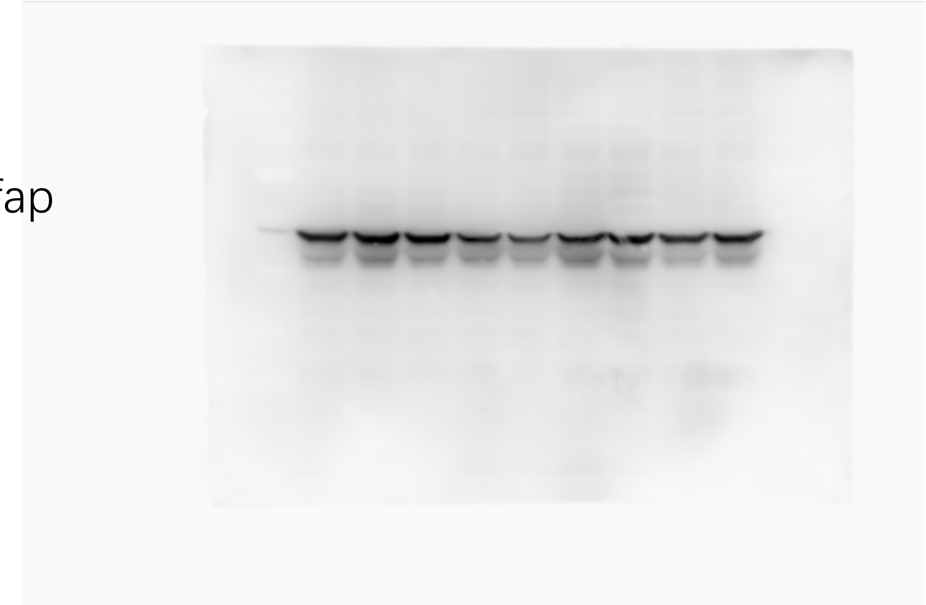

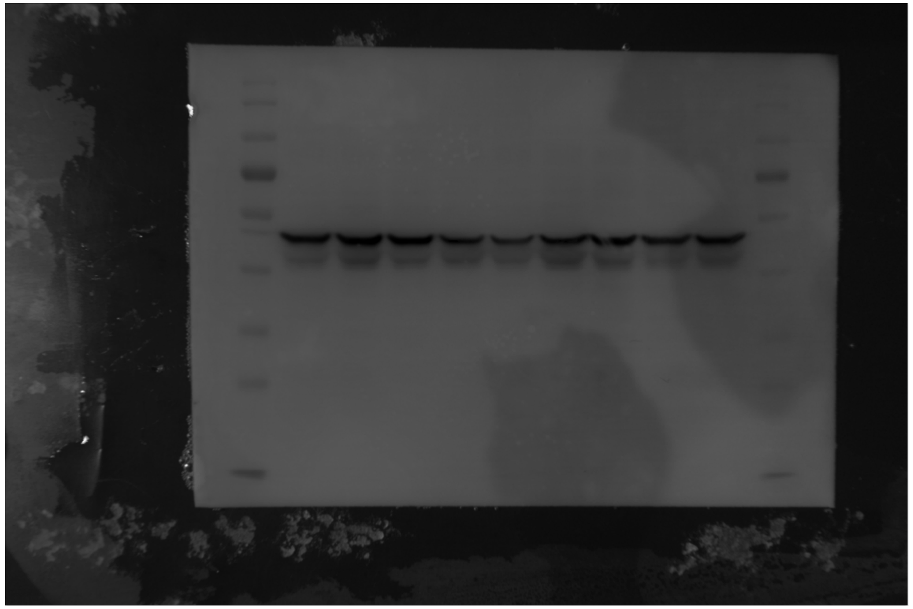

Actin（下面一条）

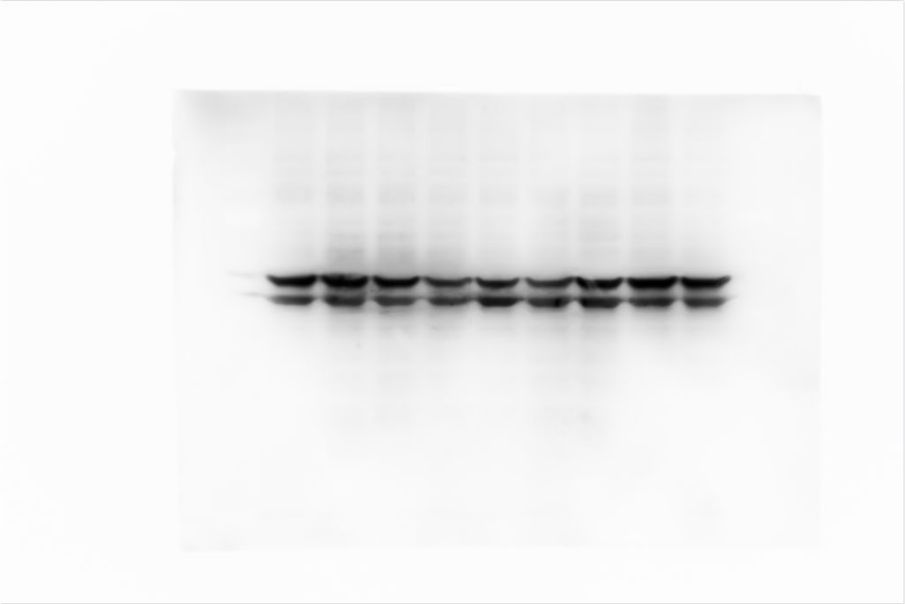

Fig.7G

Unc治疗-pstat3

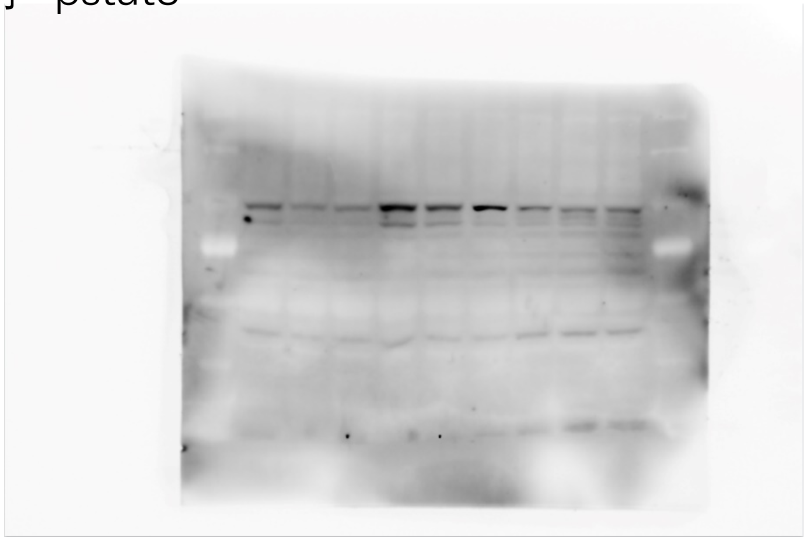

Unc治疗-stat3

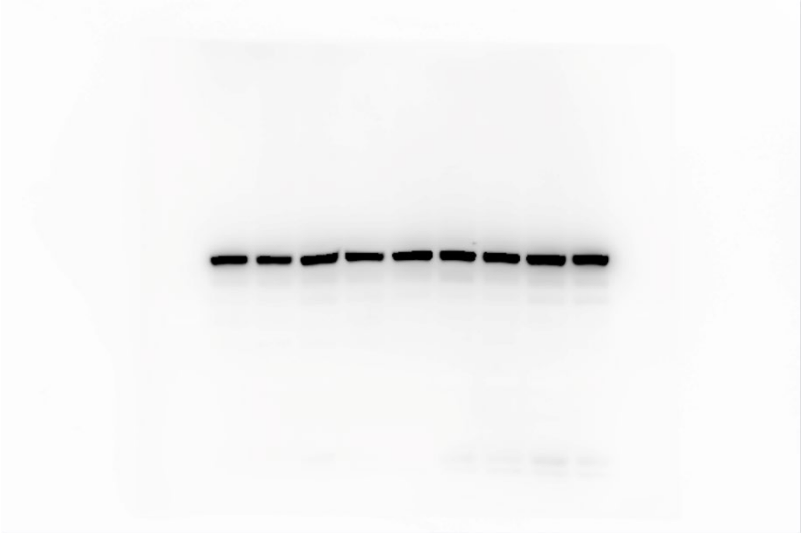

stat3-actin

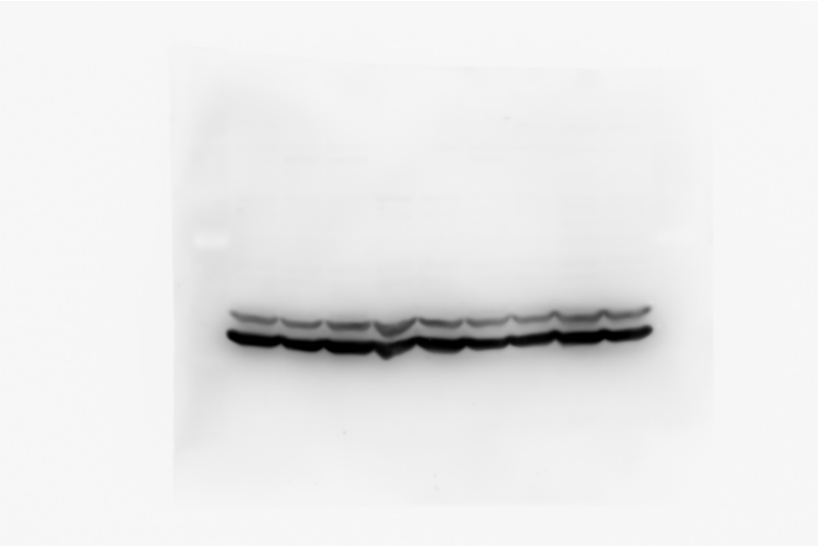

Fig.7G

Unc治疗-p-TBK1

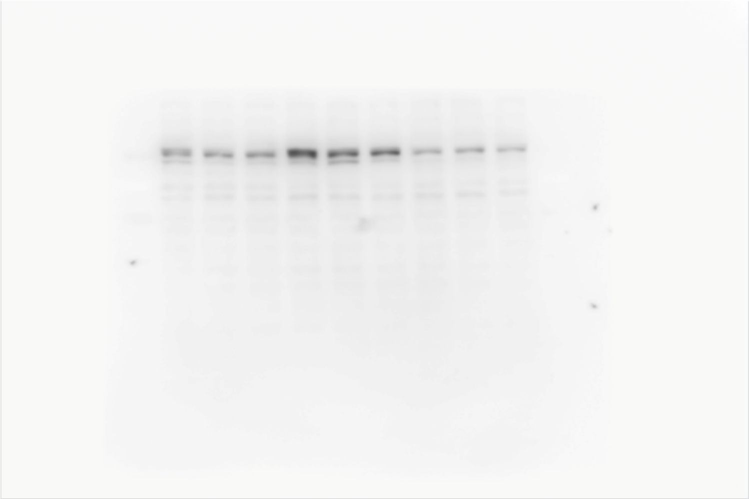

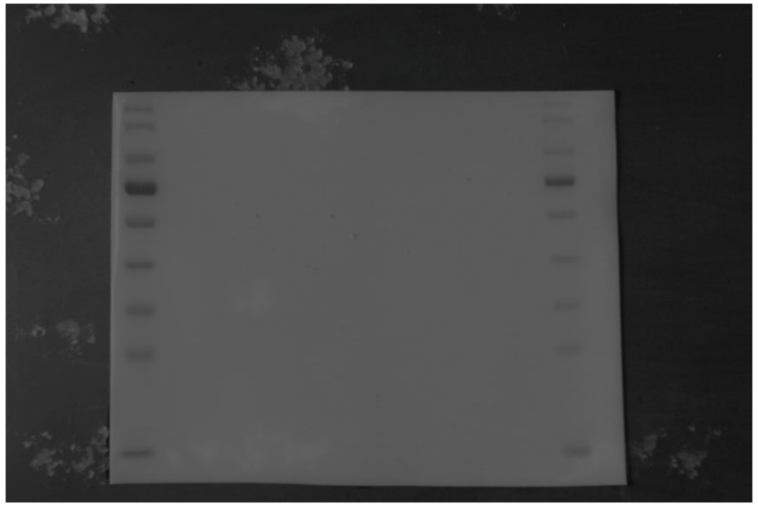

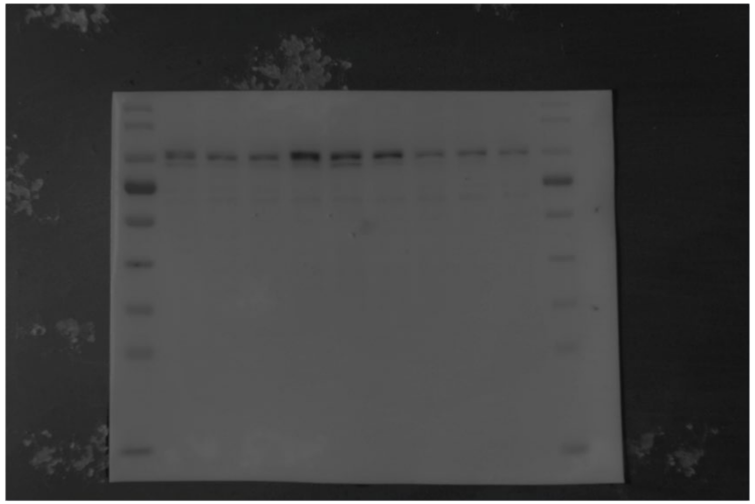

Fig.7G

Unc-cGAS

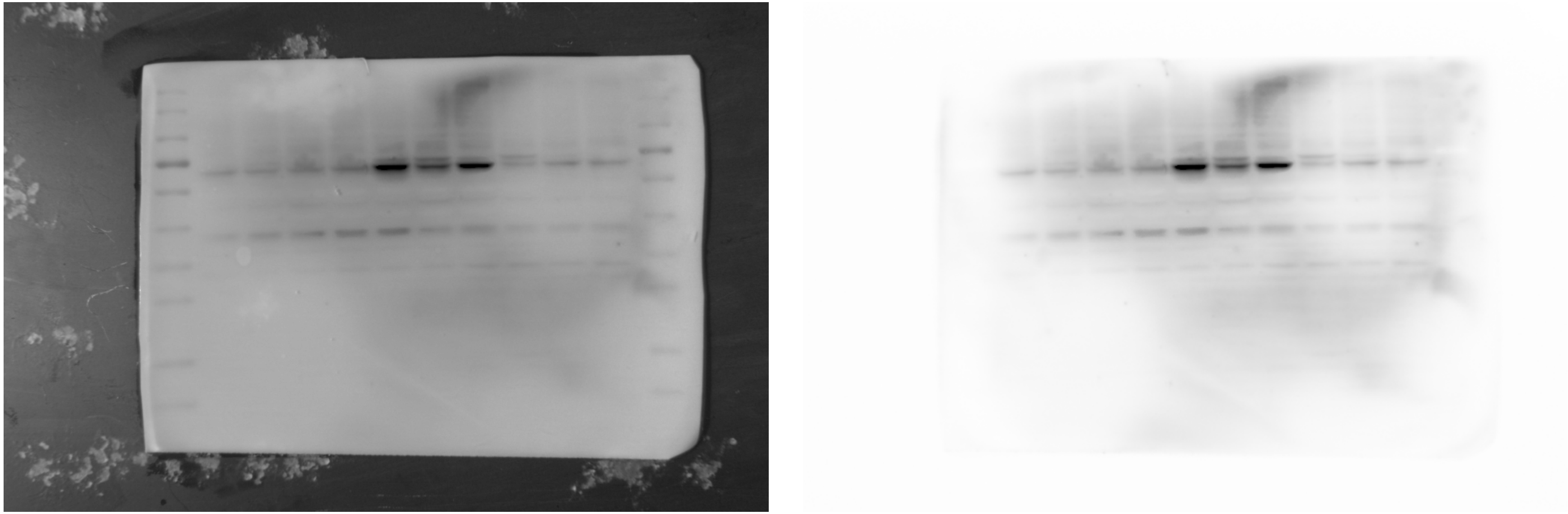

Fig.7G
Unc-STING/actin

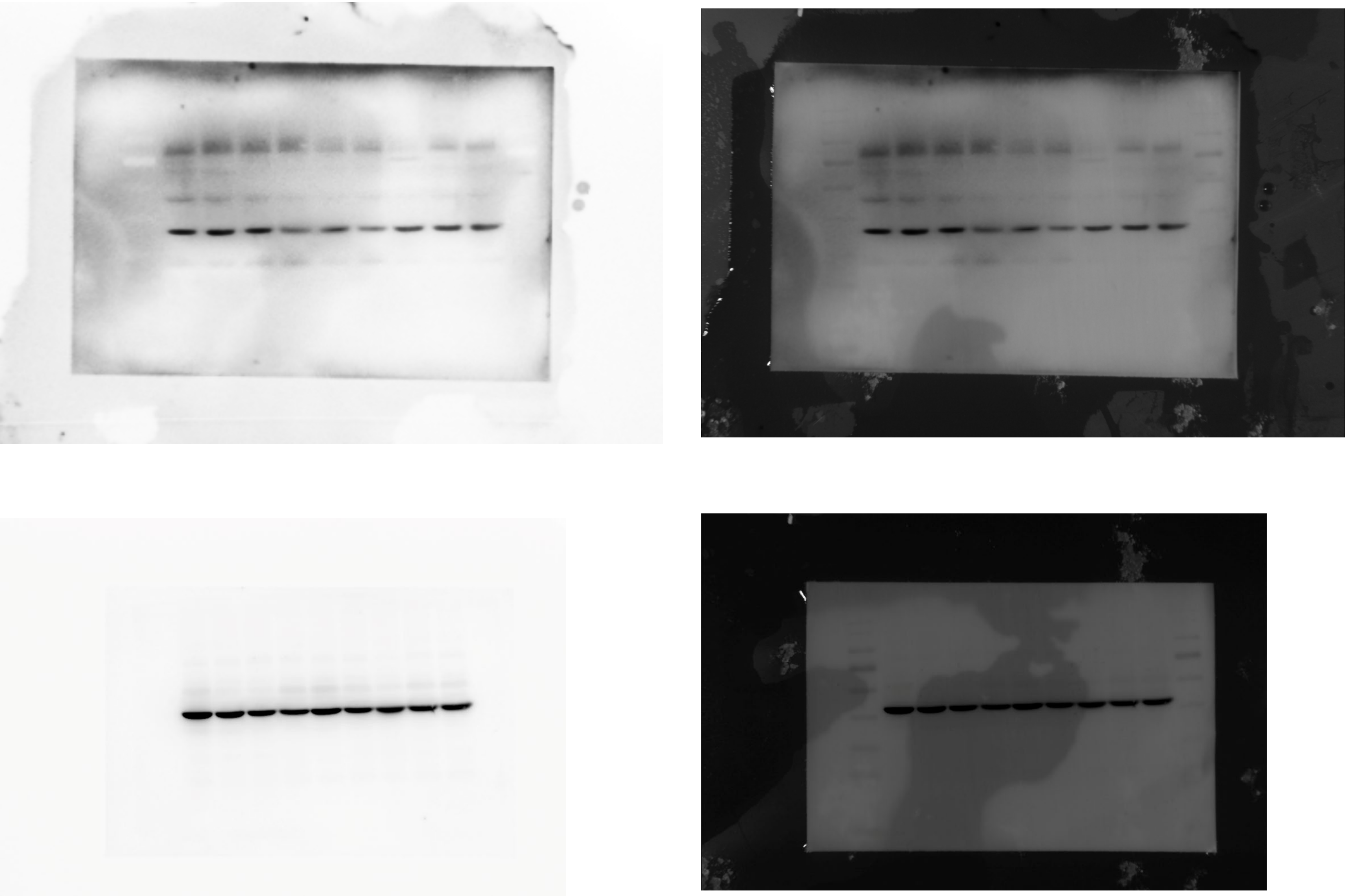

Fig.7G

Unc-TBK1

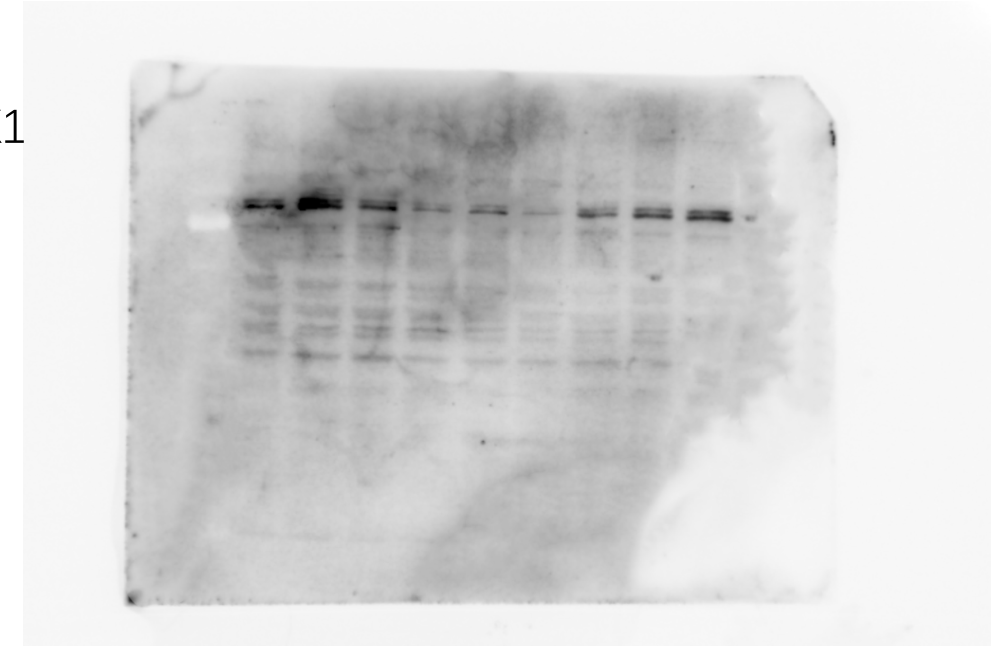

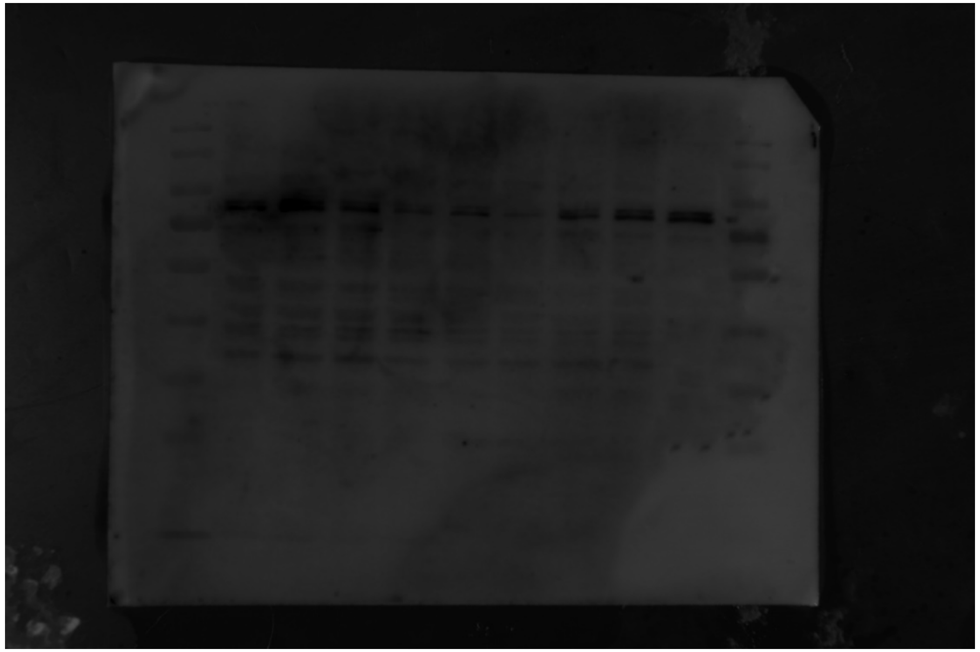

Unc-P-STING

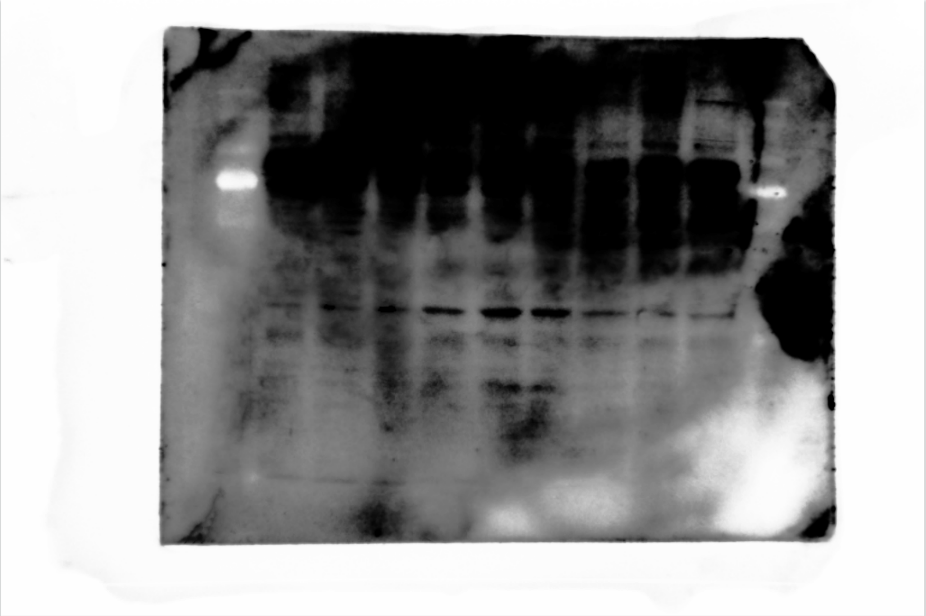

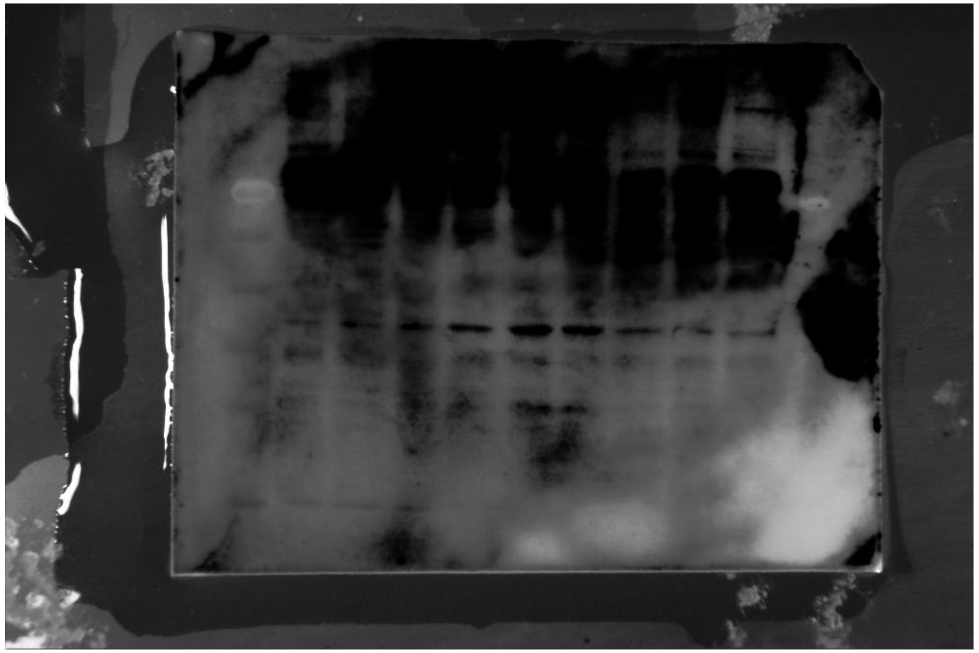

Fig.7G

Unc-ACTIN

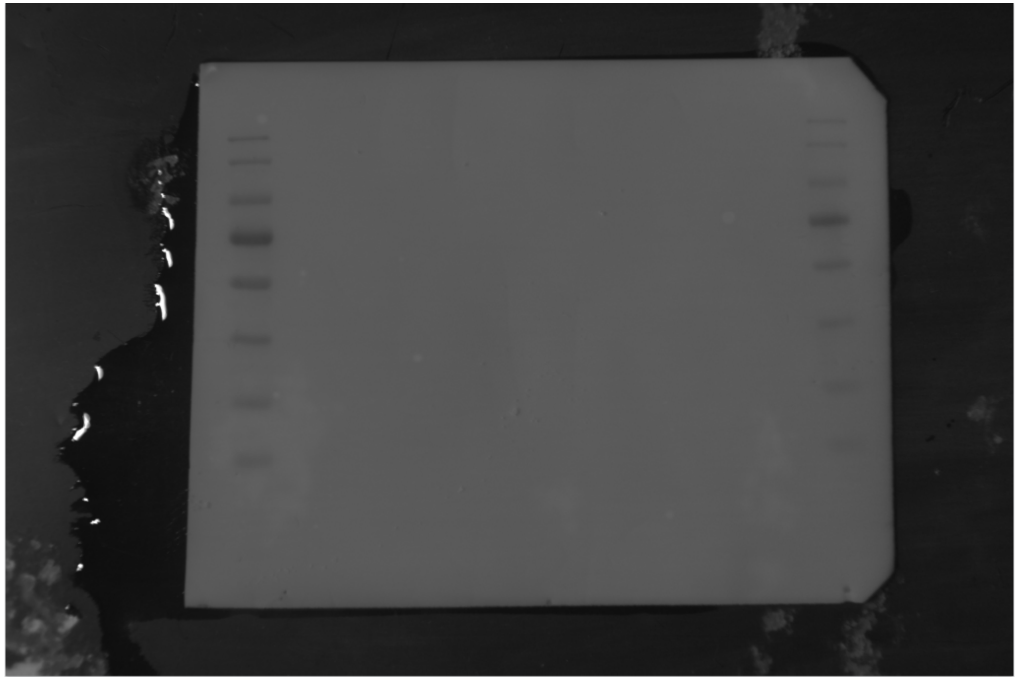

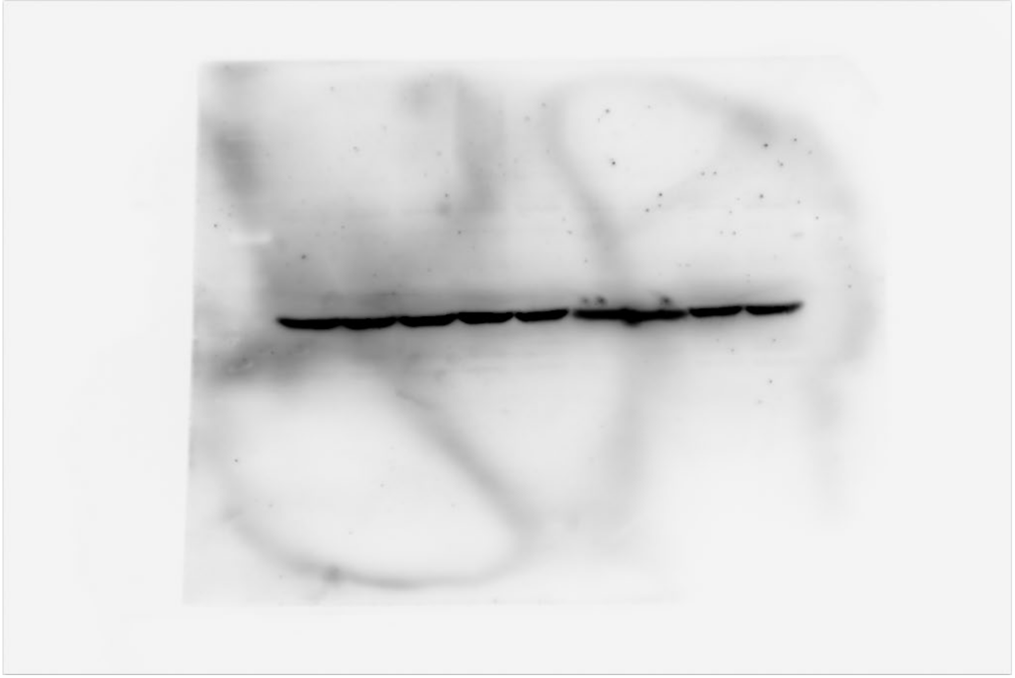

Fig.7G

Unc-irf3/actin

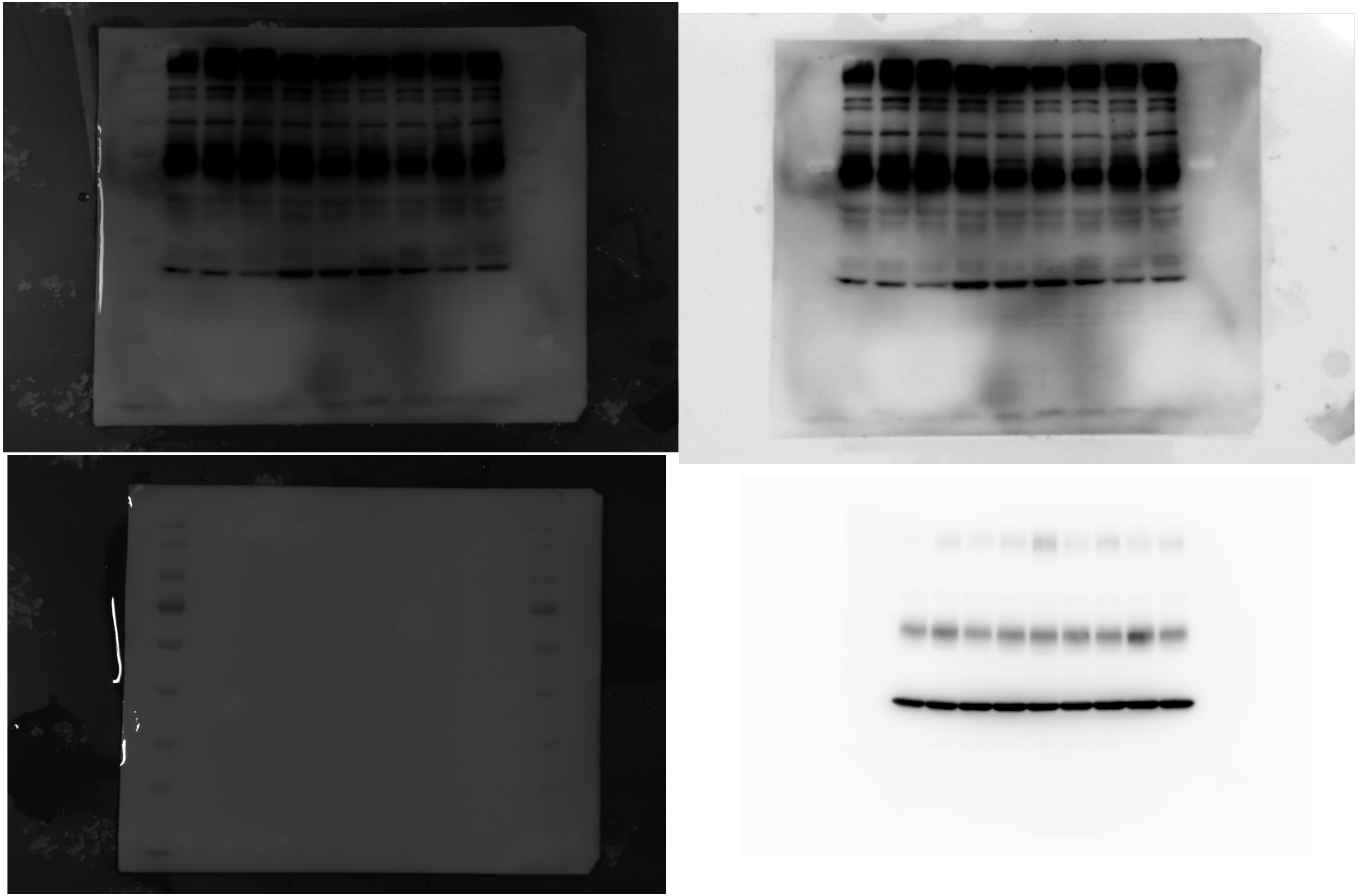

Supplement: Supplementary file 2 — Additional file 2: The original data of Western blot in the study. [file 12974_2022_2597_MOESM2_ESM.pdf]
